# SARS-CoV-2 spike-induced syncytia are senescent and contribute to exacerbated heart failure

**DOI:** 10.1371/journal.ppat.1012291

**Published:** 2024-08-05

**Authors:** Huilong Li, Luming Wan, Muyi Liu, Enhao Ma, Linfei Huang, Yilong Yang, Qihong Li, Yi Fang, Jingfei Li, Bingqing Han, Chang Zhang, Lijuan Sun, Xufeng Hou, Haiyang Li, Mingyu Sun, Sichong Qian, Xuejing Duan, Ruzhou Zhao, Xiaopan Yang, Yi Chen, Shipo Wu, Xuhui Zhang, Yanhong Zhang, Gong Cheng, Gengye Chen, Qi Gao, Junjie Xu, Lihua Hou, Congwen Wei, Hui Zhong

**Affiliations:** 1 Beijing Institute of Biotechnology, Beijing, China; 2 College of Basic Medical Sciences, School of Medicine, Zhejiang University, Hangzhou, China; 3 Tsinghua-Peking Center for Life Sciences, School of Medicine, Tsinghua University, Beijing, China; 4 The Fifth Medical Centre, Chinese PLA General Hospital, Beijing, China; 5 Beijing Yaogen Biotechnology Co.Ltd, Beijing, China; 6 Beijing Anzhen Hospital, Capital Medical University, Beijing, China; 7 Department of Pathology, Fuwai Hospital, Peking Union Medical College, Chinese Academy of Medical Science, Beijing, China; 8 People’s Hospital of Ningxia Hui Autonomous Region, Yinchuan, China; Erasmus Medical Center, NETHERLANDS, KINGDOM OF THE

## Abstract

SARS-CoV-2 spike protein (SARS-2-S) induced cell–cell fusion in uninfected cells may occur in long COVID-19 syndrome, as circulating SARS-2-S or extracellular vesicles containing SARS-2-S (S-EVs) were found to be prevalent in post-acute sequelae of COVID-19 (PASC) for up to 12 months after diagnosis. Although isolated recombinant SARS-2-S protein has been shown to increase the SASP in senescent ACE2-expressing cells, the direct linkage of SARS-2-S syncytia with senescence in the absence of virus infection and the degree to which SARS-2-S syncytia affect pathology in the setting of cardiac dysfunction are unknown. Here, we found that the senescent outcome of SARS-2-S induced syncytia exacerbated heart failure progression. We first demonstrated that syncytium formation in cells expressing SARS-2-S delivered by DNA plasmid or LNP-mRNA exhibits a senescence-like phenotype. Extracellular vesicles containing SARS-2-S (S-EVs) also confer a potent ability to form senescent syncytia without *de novo* synthesis of SARS-2-S. However, it is important to note that currently approved COVID-19 mRNA vaccines do not induce syncytium formation or cellular senescence. Mechanistically, SARS-2-S syncytia provoke the formation of functional MAVS aggregates, which regulate the senescence fate of SARS-2-S syncytia by TNFα. We further demonstrate that senescent SARS-2-S syncytia exhibit shrinked morphology, leading to the activation of WNK1 and impaired cardiac metabolism. In pre-existing heart failure mice, the WNK1 inhibitor WNK463, anti-syncytial drug niclosamide, and senolytic dasatinib protect the heart from exacerbated heart failure triggered by SARS-2-S. Our findings thus suggest a potential mechanism for COVID-19-mediated cardiac pathology and recommend the application of WNK1 inhibitor for therapy especially in individuals with post-acute sequelae of COVID-19.

## Author summary

In this paper, we directly linked SARS-2-S-triggered syncytium formation in the absence of infection with the ensuing induction of cellular senescence and its pathophysiological contribution to heart failure. We propose that both SARS-2-S expression and SARS-2-S protein internalization were sufficient to induce senescence in nonsenescent ACE2-expressing cells. This is important because of the persistent existence of SARS-2-S or extracellular vesicles containing SARS-2-S during the post-acute stages of SARS-CoV-2 infection in human subjects. In searching for the underlying molecular mechanisms determining syncytial fate, the formation of functional MAVS aggregates dependent on RIG-I was observed at an early stage during fusion and regulated the anti-death to senescence fate of SARS-2-S syncytia through the TNFα-TNFR2 axis. We also found impaired cardiac metabolism in SARS-2-S syncytia induced by condensed WNK1. However, syncytium formation or cellular senescence observed with the wild-type fusogenic S protein does not occur with the spike proteins produced by currently approved COVID-19 mRNA vaccines. Importantly, SARS-2-S-exacerbated heart failure could be largely rescued by WNK1 inhibitor, anti-syncytial drug or senolytic agent. Together, we suggest that rescuing metabolism dysfunction in senescent SARS-2-S syncytia should be taken into consideration in individuals with post-acute sequelae of COVID-19 (PASC).

## Introduction

Coronavirus disease 2019 (COVID-19), caused by severe acute respiratory syndrome coronavirus 2 (SARS-CoV-2), has resulted in a global pandemic with millions of deaths [1]. Although symptoms resulting from infection typically resolve within weeks, some individuals experience persistent symptoms following the acute phase of COVID-19, the so-called post-acute sequelae of COVID-19 (PASC) or long COVID [2–4]. SARS-CoV-2 infection predominantly offends the respiratory system. Currently, evidence has suggested cardiac complications as one of the important pathogenic features of COVID-19 [5,6]. More importantly, compared with patients without heart failure, those with diagnosed heart failure experienced a longer length of hospital stay, increased risk of mechanical ventilation and intensive care unit admission, and higher mortality [7,8]. Direct myocardial injury, inflammation and thrombosis, hypoxemia, and ACE2 downregulation are suggested to be involved in COVID-19-mediated cardiac pathology; however, the pathogenic mechanisms underlying the increased vulnerability to heart failure in patients with COVID-19 are still largely unknown [9–11].

SARS-CoV-2 syncytium constitutes a hallmark of COVID-19-associated pathology and may potentially contribute to pathology by facilitating viral dissemination, cytopathy, immune evasion, and the inflammatory response [12]. Syncytium formation is mediated by the SARS-CoV-2 spike protein (SARS-2-S). SARS-2-S interacts with the ACE2 receptor on the host cell and is then proteolytically activated by host proteases, leading to the fusion of the viral membrane with the plasma membrane [13]. In addition to mediate viral-cell fusion, the presence of SARS-2-S on the cell surface is sufficient to mediate cell-cell fusion, a mechanism called fusion from within (FFWI)[14,15]. The fusogenic activity of SARS-2-S is potent, as even undetectable amounts of SARS-2-S can cause cell–cell fusion [14]. In addition to mediating FFWI, virus-like particles or lipid vesicles containing surface SARS-2-S can serve as a bridge between two cells and induce fusion from cells without *de novo* synthesis of SARS-2-S protein (FFWO)[14,15]. Cell-cell fusion in uninfected cells may also occur in long COVID-19 syndrome, as SARS-2-S or circulating extracellular vesicles containing SARS-2-S (S-EVs) were detected predominantly in PASC patients for up to 12 months after diagnosis [16,17].

SARS-CoV-2 elicits the senescence of infected cells, similar to other viruses (virus-induced senescence, VIS) [18,19]. Cellular senescence is a state of stable cell cycle arrest characterized by increased lysosomal activity, exemplified by induction of the lysosomal enzyme senescence-associated β-galactosidase (SA-β-gal) and activation of the senescence-associated secretory phenotype (SASP) [20–22]. SARS-CoV-2 infection triggers paracrine senescence and can lead to a hyperinflammatory environment and the onset of acute respiratory disease syndrome [23]. It is not entirely clear whether virus propagation stress or specific viral molecular components induce VIS. Interestingly, isolated recombinant SARS-2-S protein has been shown to increase the SASP in senescent ACE2-expressing cells [18]. However, the direct linkage of SARS-2-S syncytia with senescence in non-senescence ACE2-expressing cells (for example, in the absence of virus infection but with chronic presence of SARS-2-S protein) and the degree to which SARS-2-S syncytia affect pathology in the setting of cardiac dysfunction are unknown.

Here, we demonstrated that both SARS-2-S expression and SARS-2-S protein internalization were sufficient to induce senescence in non-senescent ACE2-expressing cells. The formation of functional MAVS aggregates dependent on RIG-I was observed at an early stage during fusion and regulated the anti-death to senescence fate of SARS-2-S syncytia through the TNFα-TNFR2 axis. Except for SASP, shrinking of SARS-2-S syncytia triggered WNK1 activation, which led to impaired cardiac metabolism. SARS-2-S exacerbated heart failure progression, a phenotype that could be largely rescued by WNK1 inhibitors, anti-syncytial drugs or senolytic agents. Together, we suggest that rescue metabolism dysfunction of senescent SARS-2-S syncytia should be taken into consideration in individuals with a history of heart failure during the acute or post-acute stage of SARS-CoV-2 infection.

## Results

### SARS-2-S and its variant-expressing syncytia exhibit a senescence-like phenotype

**Fig 1:**
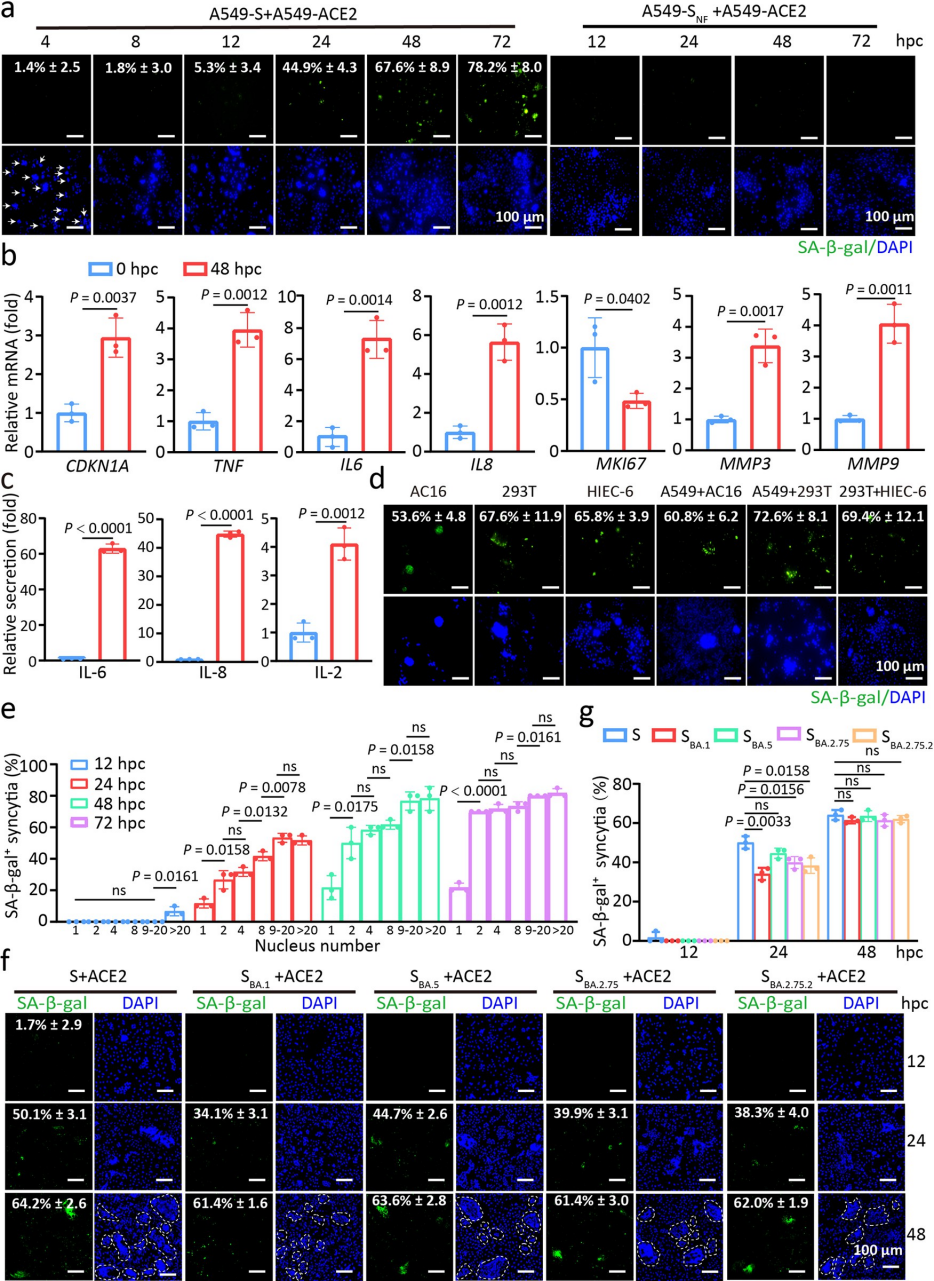
SARS-2-S–expressing syncytia exhibit a senescence-like phenotype. a, SA-β-gal staining of A549-SARS-2-S (SARS-2-S hereafter referred to S) or A549-S_NF_ (R682A, R683A, R685A, K814A, and R815A mutant) cocultured with A549-ACE2 cells for the indicated h (hpc). Green, SA-β-gal staining; blue, nuclear DAPI staining. Scale bars represent 100 μm. b, Normalized expression of *CDKN1A*, *TNF*, *IL6*, *IL8*, *MKI67*, *MMP3* and *MMP9* transcripts in cocultured A549 cells at 48 hpc relative to that at 0 hpc by RT-qPCR. c, IL-6, IL-8, and IL-2 protein levels in conditioned medium from cells in b by ELISA. d, SA-β-gal staining of S–expressing cells cocultured with the same or different cell types. Scale bars represent 100 μm. e, Quantification of SA-β-gal positivity in cocultured A549 cells with different nuclei numbers during fusion for the indicated hpc. f, g, SA-β-gal staining (f) SA-β-gal-positivity quantification (g) of S, Omicron variant BA.1 spike (S_BA.1_), Omicron variant BA.5 spike (S_BA.5_), Omicron variant BA.2.75 spike (S_BA.2.75_), and Omicron variant BA.2.75.2 spike (S_BA.2.75.2_)-transfected A549 and A549-ACE2 cells cocultured for the indicated hpc. Scale bars represent 100 μm. All images are representative of 3 biological replicates. All quantified data are presented as the mean ± SD of n = 3 independent experiments. Statistical significance was determined using two-tailed Student’s t test (b, c) or one-way ANOVA and Bonferroni’s post hoc analysis (e, g).

Recombinant SARS-2-S protein induces senescence in ACE2-expressing cells. To explore whether SARS-2-S expression was sufficient to induce senescence in nonsenescent ACE2-expressing cells, we cocultured SARS-2-S (Wuhan-Hu-1)-expressing A549 cells with ACE2-expressing A549 cells at a 1:1 ratio (S1A and S1B Fig). Syncytial cells rapidly appeared at 4 h post coculture (hpc) and gradually grew in size, as indicated by microscopic analysis (Fig 1A). Notably, SARS-2-S-expressing syncytia (hereafter referred to as SARS-2-S syncytia) with enhanced SA-β-gal staining began to appear at 12 hpc, and the percentage of SA-β-gal-positive syncytia among SARS-2-S syncytia gradually increased during the fusion process (Fig 1A). In contrast to SARS-2-S, a SARS-2-S_NF_ mutant (R682A R683A R685A K814A R815A), which fails to cause fusion because it cannot be cleaved by host protease, lost the ability to induce senescence [24,25] (Figs 1A and S1C). We did not observe multinucleated cells or SA-β-gal–positive staining in A549 cells that infected by empty lentivirus (eLV) or expressed only SARS-2-S or ACE2, when these cells were co-cultured with A549-eLV cells at the time points tested (S1D Fig). Bright field and SA-β-gal staining were also performed to validate the specificity of the results in SARS-2-S syncytia compared to SARS-2-S_NF_ (S1E and S1F Fig). Quantitative real-time PCR (RT-qPCR) revealed significant transcriptional upregulation of senescence-associated genes (Fig 1B). The upregulation was fusion specific, as S_NF_, S, ACE2 and S_NF_+ACE2 cells did not exhibit such upregulation (S1G Fig). Consistently, the conditioned medium of SARS-2-S syncytia at 48 hpc contained significantly more SASP-related cytokines, such as IL-6, IL-8, and IL-2, than that at 0 hpc compared to SARS-2-S_NF_ mutant (Fig 1D). We found that higher increased levels in secreted proteins than mRNA levels of IL-6 and IL-8. This may be due to the continued expression and secretion of the protein after the transcription level was up-regulated, and the secreted protein in the supernatant has a cumulative effect. In a similar manner, we observed SARS-2-S–induced senescence during coculture of the same and different cell types, including AC16 (Human cardiomyocyte cell line), HEK293T (The human embryonic kidney 293 cell line) and HIEC-6 (intestinal epithelial cell line) and (Fig 1D). The quantification of syncytia for different cell types was assessed using α-complementation assay as reported [14]. Herein, A549 cell model was presented as an example (S1H and S1I Fig). In A549 cocultured cells, the appearance of SA-β-gal-positive staining was associated with the syncytial nuclei number. Eventually, over 70% of SARS-2-S syncytia exhibited positive SA-β-gal staining at 72 hpc (Fig 1E). We next compared the senescence potential of four Omicron variants, SARS-2-S_BA.1_, SARS-2-S_BA.5_, SARS-2-S_BA.2.75_, and SARS-2-S_BA.2.75.2_, with that of SARS-2-S (S1C Fig). As reported, the Omicron BA.1 variant showed significantly weaker fusogenic activity than SARS-2-S [26] (5× reduction). The fusogenic capacity of BA.5 (1.2× reduction), BA.2.75 (1.7× reduction), and BA.2.75.2 (1.7× reduction) was modestly lower than that of SARS-2-S [27,28] (S1J Fig). Notably, the percentage of SA-β-gal-positive syncytia was comparable to that of SARS-2-S, although they formed more slowly (Fig 1F and 1G). Furthermore, SARS-2-S syncytia could be selectively killed by senolytic drugs, including the multiple-kinase inhibitor fisetin, the tyrosine kinase inhibitor dasatinib, and the BCL-2–specific venetoclax, but these senolytic drugs could not affect cell viability under conditions where fusion is inhibited by using S_NF_ mutants (S1K Fig). Taken together, the above observations indicate that SARS-2-S elicits a senescence-like phenotype in an FFWI manner.

### SARS-2-S delivery by EVs or mRNA confers the ability to trigger syncytial senescence

**Fig 2:**
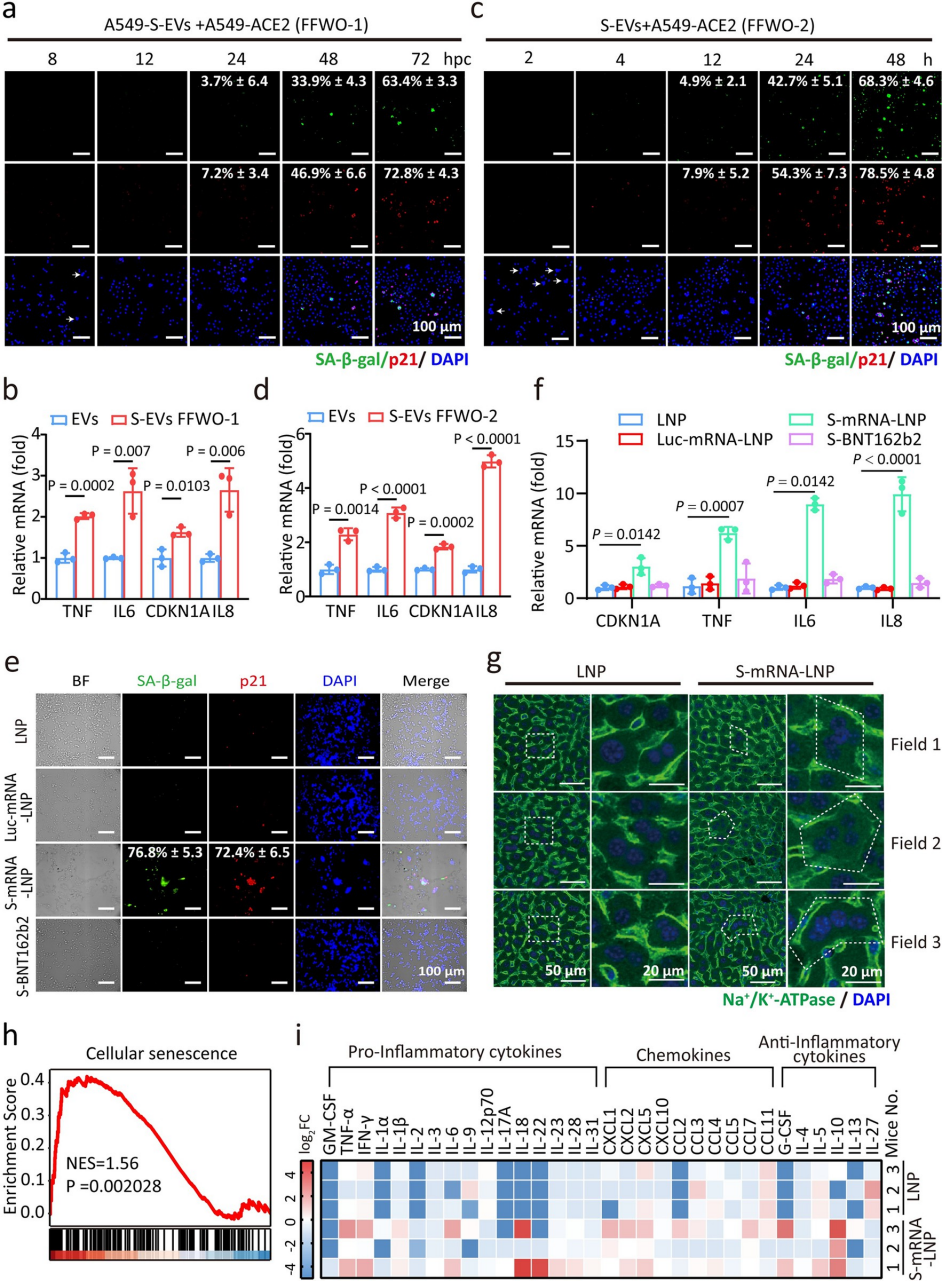
SARS-2-S delivery by EVs or mRNA triggers syncytial senescence. a, c, e, SA-β-gal and p21 staining of A549-S-EVs and A549-ACE2 cocultured cells (a, fusion from without (FFWO-1)) or A549-ACE2 cells treated with S-EVs (c, FFWO-2) or A549-LNP/A549-Luc-mRNA-LNP/A549-S-mRNA-LNP/A549-S-BNT162b2 and A549-ACE2 cocultured cells (e) for the indicated times. Green, SA-β-gal staining; red, p21 staining; blue, DAPI staining. Scale bars represent 100 μm. b, d, f, Normalized expression of *TNF*, *IL6*, *CDKN1A*, *and IL8* in A549 cells at 24 hpc from a (b), c (d), and e (f) by RT-qPCR. g, Immunohistochemical analysis of liver tissues from mice intramuscularly injected with a single dose of S-mRNA-LNP (0.12 mg/kg) or LNP at 24 h after injection. Green, Na^+^/Ka^+^-ATPase staining of the cell membrane; blue, DAPI staining. Scale bars represent 50 μm or 20 μm as indicated. h, Gene set enrichment analysis (GSEA) of genes related to cellular senescence in the livers from mice in g. NES, normalized enrichment score. i, Multiplex bead-based protein analysis in the serum from mice in g. All images are representative of 3 biological replicates. All quantified data are presented as the mean ± SD of n = 3 independent experiments. Statistical significance was determined using two-tailed Student’s *t* test (b, d, f).

Since SARS-2-S was shown to be incorporated into extracellular vesicles circulating in patient blood [29], we then asked if SARS-2-S protein internalization by S-EVs has the ability to trigger syncytium formation and cell senescence in an FFWO-dependent manner. To this end, we first used ultracentrifugation to purify EVs from the supernatant of HeLa- or SARS-2-S-expressing HeLa cells and confirmed their identity by probing for exosome markers and the EVs were normalized by NTA (S2A–S2C Fig). We next labelled these EVs with the fluorescent dye DiO and exposed A549 cells to the EVs, and the expression of SARS-2-S on the surface of the cells that took up the S-EVs was observed (S2D and S2E Fig). These cells fused with cells expressing ACE2 at 8 hpc, as indicated by the appearance of multinucleated cells (Fig 2A). Importantly, the fused cells exhibited enhanced SA-β-gal and p21 staining, with increased transcription of the *TNF*, *IL6*, *IL8* and *CDKN1A* genes (Fig 2B), features that are typical of senescence. We also noted the senescence phenotype in other similarly treated cell types, but not in cells that took up EVs lacking SARS-2-S (S2F and S2G Fig). In addition, A549-ACE2 cells were incubated with S-EVs directly for 2 to 48 h. Notably, S-EVs induced syncytium formation as early as 2 h, a timeframe in which adjacent cells may fuse directly without S-EVs uptake and SARS-2-S presentation at the cell surface. The appearance of senescent syncytia began to occur at 12 h (Fig 2C and 2D). Eventually, approximately 70% of syncytia exhibited positive SA-β-gal staining at 48 h upon S-EVs addition (Fig 2C). As a control, the introduction of EVs and S-EVs into A549 cells lacking ACE2 did not induce fusion and senescence (S2H Fig). The quantification of syncytia for FFWO-1 and FFWO-2 were also assessed using α-complementation assay (S2I and S2J Fig). Therefore, SARS-2-S protein internalization was sufficient to induce senescence in an FFWO manner.

Since COVID-19 mRNA vaccines, including Pfizer and Moderna, express S protein antigen by delivering SARS-2-S mRNA, we next asked whether SARS-2-S_WT_ and S-protein-based vaccine (SARS-2-S_V_) delivered in the form of encoding mRNA would trigger syncytium formation and cell senescence (S3A Fig). Previous study indicated, compared to SARS-2-S_WT_, two prolines were added to stabilize the structure’s prefusion shape of S-protein in COVID-19 vaccine, this mutation greatly reduces cell fusion of COVID-19 vaccine [30,31]. In addition, exogenous mRNA itself could induce the innate immune response, and senescence can be induced by multiple stressors, a negative control of mRNA expression luciferase was included. We packaged the mRNA encoding luciferase, SARS-2-S_WT_ or SARS-2-S_V_ into a lipid nanoparticle formulation. Western blotting confirmed the expression of SARS-2-S treated with S_WT_-mRNA-LNP or S_V_-mRNA-LNP (S3B Fig). Next, we cocultured three types of mRNA-LNP-treated A549 cells with A549-ACE2 cells at a 1:1 ratio. Notably, syncytial cells formed rapidly and began to stain positive for SA-β-gal and p21 at 12 hpc in S_WT_-mRNA-LNP (S3C Fig). Significant upregulation of senescence-associated genes supported the presence of a senescence phenotype in S_WT_ -mRNA-LNP (Fig 2F). We did not observe syncytial cells formation, signs of senescence and significant transcriptional upregulation of senescence-associated genes in LNP-, luciferase-mRNA-LNP- or S(BNT162b2)-mRNA-LNP-treated A549 cells (Figs 2E and S3D). This finding indicates that SARS-2-S has the ability to trigger syncytial senescence regardless of its manner of delivery or expression of a non-fusogenic protein. Consistent with previous studies, since the S sequence of the COVID19 mRNA vaccine contains two prolines’ mutations, which prevents cell fusion formation, and we observed that the S_V_-mRNA-LNP could not induce cell senescence (Fig 2E). Also, the quantification of syncytia for S-mRNA was assessed using α-complementation assay (S3D and S3E Fig).

Finally, we explored whether cell senescence related to SARS-2-S-mediated cell fusion would also occur *in vivo*. To this end, we injected AAV-hACE2 constructs into the tail veins of 8-week-old C57BL/6J mice to drive mainly hepatic hACE2 expression (S3F and S3G Fig). Four weeks later, we intramuscularly administered a single dose of S-mRNA-LNP or LNP to hACE2-expressing mice and examined the induction of senescence. Immunohistochemical analysis revealed robust SARS-2-S expression and the increased appearance of multinucleated cells with at least four nuclei in the livers of S-mRNA-LNP-treated mice compared to the livers of LNP-treated mice (Figs 2G and S3H). RNA sequencing (RNA-seq) of liver samples revealed the upregulated expression of cellular senescence-related genes (Fig 2H). KEGG pathway analysis of differentially expressed genes (DEGs) revealed enrichment cellular senescence pathways (S3I Fig). Finally, multiplex protein analysis of serum samples from these animals revealed that S-mRNA-LNP strongly induced SASP-reminiscent cytokines (Fig 2I). We therefore concluded that SARS-2-S delivery in the form of DNA, EVs, or mRNA can trigger the formation of syncytia with a senescence phenotype.

### SARS-2-S syncytia provoke the formation of functional MAVS aggregates

**Fig 3:**
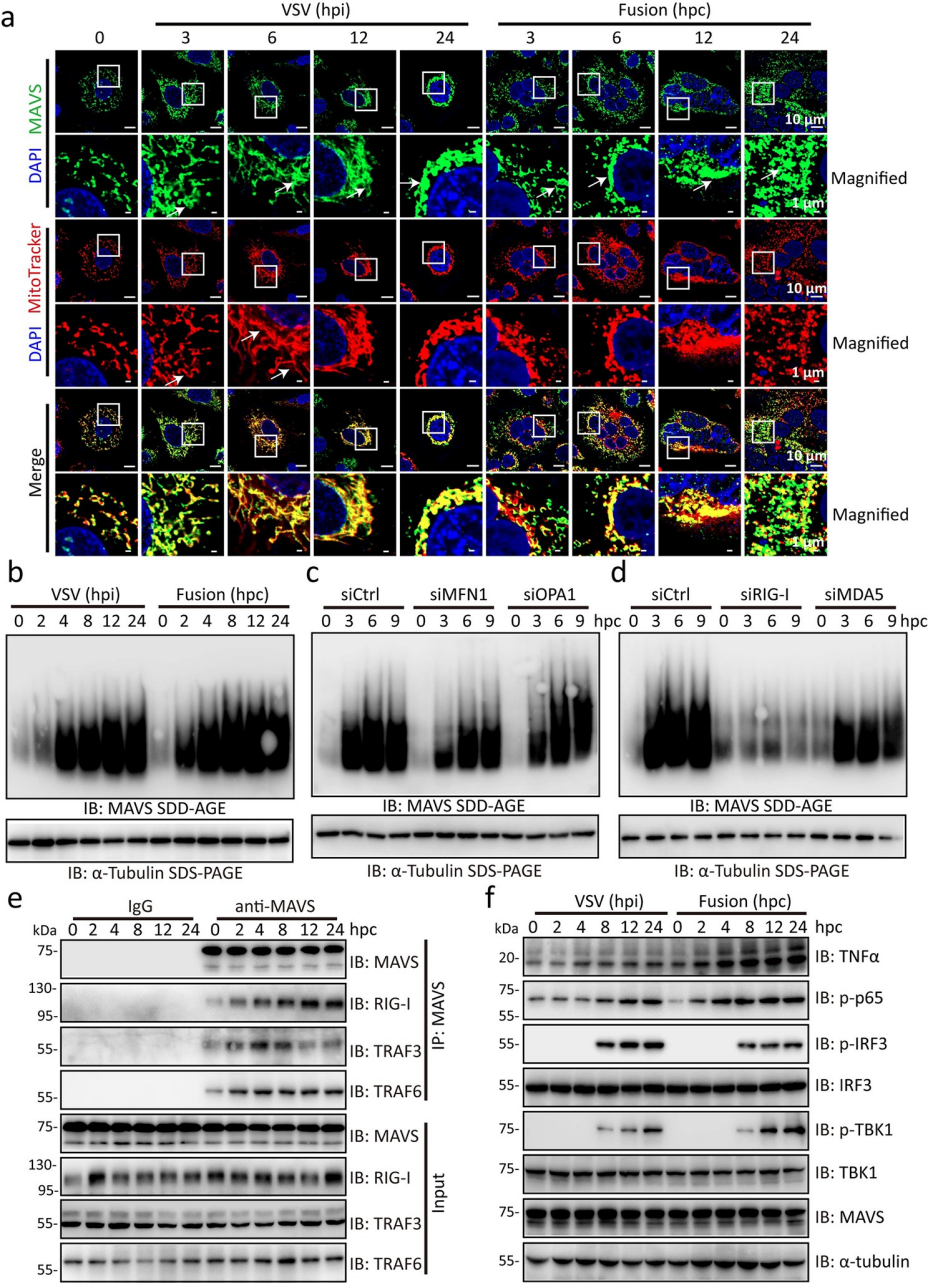
SARS-2-S syncytia induce the RIG-I–dependent formation of functional MAVS aggregates. a, Confocal microscopic images of MAVS and mitochondria in A549 cells infected with VSV or A549 cells cocultured for the indicated times. Red, MitoTracker staining for mitochondria; green, MAVS staining; blue, DAPI staining. A magnified view of the boxed region is also shown. Scale bars represent 10 μm (whole image) and 1 μm (magnified). b, MAVS aggregation in VSV-infected A549 cells or A549 cells cocultured for the indicated times by SDD-AGE with an anti-MAVS antibody. SDS-PAGE immunoblotting of α-tubulin was used as a loading control. c, d, MAVS aggregation in sicontrol (siCtrl), siMFN1, and siOPA1 (c) or siCtrl, siRIG-I, and siMDA5 (d) A549 cells cocultured for the indicated times. All these siRNA oligos were transfected into A549-S and A549-ACE2 cells 24 h before the start of coculture. α-Tubulin was used as the loading control. MAVS aggregates were analysed by SDD-AGE as in b. e, Immunoprecipitation analysis of the MAVS interaction with RIG-I, TRAF3, and TRAF6 in A549 cells cocultured for the indicated times. f, Immunoblotting analysis of proteins of the activated IFN-I signalling pathway in VSV-infected A549 cells or A549 cocultured cells for the indicated times.α-Tubulin was used as the loading control. All images are representative of three independent experiments.

Cell-cell fusion in the absence of SARS-CoV-2 infection activates the cGAS-STING pathway, which has been suggested to promote cellular senescence[32]. However, the occurrence of senescence in SARS-2-S syncytia formed by 293T cells lacking STING expression [33] indicated the existence of other signalling pathways that regulate the senescence fate of SARS-2-S syncytia. We then focused on mitochondrial antiviral signalling adaptor protein (MAVS), a mitochondrial adaptor protein that links the cytoplasmic RNA sensor RIG-I to its downstream signalling molecules by forming well-ordered prion-like aggregates [34]. As reported, the MAVS staining pattern became noticeably speckled in response to in A549 cells infected with vesicular stomatitis virus (VSV) [35] (Fig 3A). Strikingly, MAVS was also redistributed as early as 3 hpc in cocultured SARS-2-S-expressing A549 cells with ACE2-expressing A549 cells in a manner similar to its distribution in mitochondria (Figs 3A and S4A). Furthermore, MAVS aggregation during cell fusion proceeded in a manner similar to that induced by VSV infection, as determined by semidenaturing detergent agarose gel electrophoresis (SDD-AGE) (Fig 3B). While VSV infection began to trigger massive mitochondrial elongation and tubularization at 6 h post infection (hpi), we observed enhanced speckled staining of the mitochondria in SARS-2-S syncytia, with relatively less mitochondrial fusion, during the entire fusion process (Fig 3A). Importantly, MAVS aggregation was reduced by knockdown of mitofusin 1 (MFN1) but not by knockdown of OPA1; both of these proteins are mitochondrial membrane proteins that regulate mitochondrial dynamics and RIG-I-induced antiviral signalling [36] (Figs 3C and S4B). Accordingly, we knocked down the RIG-I gene (S4B Fig). We found that this significantly reduced MAVS aggregation in cocultured cells (Fig 3D). MAVS aggregation in cocultured cells was also reduced by MDA5 silencing, but this effect was much less pronounced than that observed upon RIG-I silencing, although the knockdown efficiency was approximately 70% for both genes (Figs 3D and S4B). These data suggest the involvement of RIG-I, the primary sensor of nonself-derived dsRNA, in functional MAVS aggregation in SARS-2-S syncytia.

To confirm the spatiotemporal events linking RIG-I and MAVS during fusion, we performed a kinetic analysis of MAVS-RIG-I complex formation during cell fusion. Following the immunoprecipitation of endogenous MAVS, we detected increased levels of the RIG-I, TRAF3, and TRAF6 proteins in the immunoprecipitates as early as 2 hpc (Fig 3E). In particular, the fusion process was accompanied by increased phosphorylation levels of p65, IRF3, and TBK1, a measure of IFN-I signalling activity (Fig 3F). Finally, the activity of the NF-κB promoter was dramatically enhanced in cocultured cells (S4C Fig). The above observations indicate the activation of RIG-I-MAVS signalling in SARS-2-S syncytia.

### MAVS-TNFα signalling regulates the senescence fate of SARS-2-S syncytia

**Fig 4:**
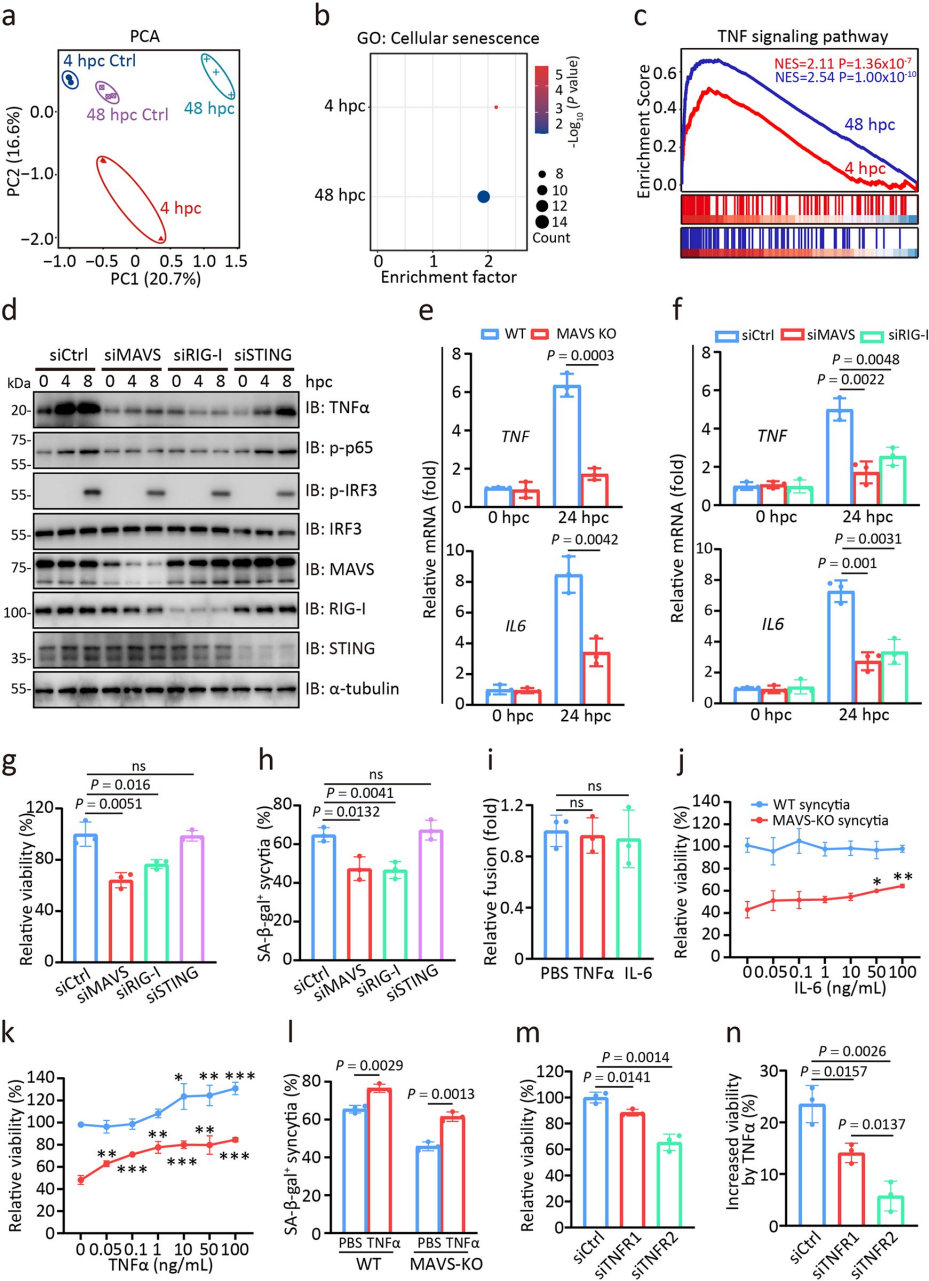
MAVS–TNFα signalling regulates the survival to senescence fate of SARS-2-S syncytia. a, PCA of gene expression patterns in A549 cells cocultured for the indicated times. In all plots, each point represented an independent biological replicate. b, Senescence pathways enriched in the samples analysed in a based-on GO term analysis. c, GSEA of genes related to the TNF signalling pathway among the DEGs in cells from a. d, Immunoblotting analysis of proteins of the activated IFN-I signalling pathway in siCtrl, siMAVS, siRIG-I, or siSTING cells cocultured for the indicated times. All these siRNA oligos were transfected into A549-S and A549-ACE2 cells 24 h before the start of coculture. α-Tubulin was used as the loading control. e, f, Normalized expression of *TNF* and *IL6* in WT and MAVS-KO cocultured 293T cells (e) or siCtrl, siMAVS, and siRIG-I (f) cocultured 293T cells at 24 hpc relative to that at 0 hpc. g, Relative cell viability of siMAVS, siRIG-I, or siSTING cocultured cells at 24 hpc by CCK8 assay. The cell viability of siCtrl cocultured cells at 24 hpc was set to 100%. All these siRNA oligos were transfected into A549-S and A549-ACE2 cells 24 h before coculture. h, SA-β-gal positivity in cocultured cells from g. i, Relative fusion of cocultured A549 cells at 24 hpc in the presence of TNFα (100 ng/mL) or IL-6 (100 ng/mL) by β-galactosidase assay. The fusion level of cocultured A549 cells treated with PBS was set to 1. j, k, Relative cell viability of WT or MAVS-KO cocultured cells at 48 hpc in the presence of IL-6 (j) or TNFα (k). The cell viability of WT cocultured cells at 48 hpc was set to 100%. l, SA-β-gal positivity in WT or MAVS-KO cocultured cells at 48 hpc in the presence of TNFα. m, Relative viability of siTNFR1- or siTNFR2-cocultured A549 cells at 24 hpc. The viability of siCtrl cells at 24 hpc was set to 100%. n, Increased cell viability of siCtrl, siTNFR1, or siTNFR2 cocultured cells at 48 hpc in the presence of TNFα than that in the presence of PBS. All quantified data are shown as the mean ± SD of n = 3 independent experiments. Statistical significance was determined using two-tailed Student’s *t* test (e) or one-way ANOVA and Bonferroni’s post hoc analysis (f, g, h, i, j, k, l, m, n).

We next attempted to identify factors that determine the senescence fate of SARS-2-S syncytia. To this end, we systematically analysed the gene expression of cocultured A549 cells by RNA-seq at 4 and 48 hpc. Principal component analysis (PCA) of the data revealed that samples obtained after coculture for these durations did not overlap and were well separated from their respective controls (Fig 4A). Gene Ontology (GO) enrichment analysis indicated that the coverage of senescence- and SASP-related GO terms increased with increasing coculture time (Fig 4B). This finding supported the notion that fusogenic SARS-2-S gradually triggers a senescence stress response. Importantly, KEGG pathway analysis revealed enrichment in the TNFα and NF-κB signalling pathways throughout the fusion process (Figs 4C, S5A and S5B). Notably, MAVS or RIG-I silencing significantly reduced the activation of NF-κB, IRF3, and TNFα (Fig 4D). Furthermore, the expression of TNFα and IL6 was also largely abolished in MAVS KO-, siRIG-I-, or siMAVS-cocultured 293T cells (Fig 4E and 4F). In A549 cells with intact cGAS-STING signalling, syncytial survival and the senescent syncytial subpopulation were also significantly reduced by MAVS or RIG-I knockdown, but not by STING knockdown (Fig 4G and 4H). These results indicated the essential role of RIG-I-MAVS in regulating the senescence fate of the SARS-2-S syncytium.

To determine whether MAVS-mediated NF-κB signalling induction is required for the survival and senescence of SARS-2-S syncytia, we supplemented WT and MAVS KO cocultured 293T cells with increasing concentrations of recombinant human TNFα and IL-6. While pretreatment with TNFα and IL-6 before coculture did not affect SARS-2-S fusogenic potential (Fig 4I), recombinant IL-6 at concentrations over 50 ng/mL improved the viability of MAVS KO cocultured cells, and recombinant TNFα at concentrations above 0.05 ng/mL improved the viability of MAVS KO cocultured cells (Figs 4J, 4K, S6A and S6B). Notably, the percentage of SA-β-gal-positive syncytia was also significantly increased by TNFα, especially in MAVS KO cells (Fig 4L). TNFα exerts its activity by stimulating its receptors, TNFR1 and TNFR2, which trigger distinct and common signalling pathways that control cell apoptosis and survival [37–39]. We observed that TNFR2 *(TNFRSF1B)* was selectively upregulated in cocultured A549 cells at both the early and late stages of the fusion process (S6C Fig). Indeed, knockdown of TNFR1 and TNFR2 significantly reduced the viability of cocultured cells (Fig 4M). Remarkably, TNFR1 knockdown blocked the increase of cocultured cell viability induced by TNFα, but it was not as effective as TNFR2 knockdown (Fig 4N). We also found that knockdown of TNFR1, TNFR2, MAVS, RIG-I and STING could not affect cell viability under conditions where fusion is inhibited by using S_NF_ mutants (S6D Fig). Furthermore, neither TNFR1 nor TNFR2 deficiency affected SARS-2-S fusogenicity (S6E Fig). Three siRNAs for each gene were designed and the highest efficacy in downregulation was chosen for formal experiment (S6F–S6J Fig). Taken together, these observations suggest that the MAVS-TNFα-TNFR2 axis regulates the anti-death to senescence fate of SARS-2-S syncytia.

### Senescent SARS-2-S syncytia shrinkage triggers WNK1 phase separation

**Fig 5:**
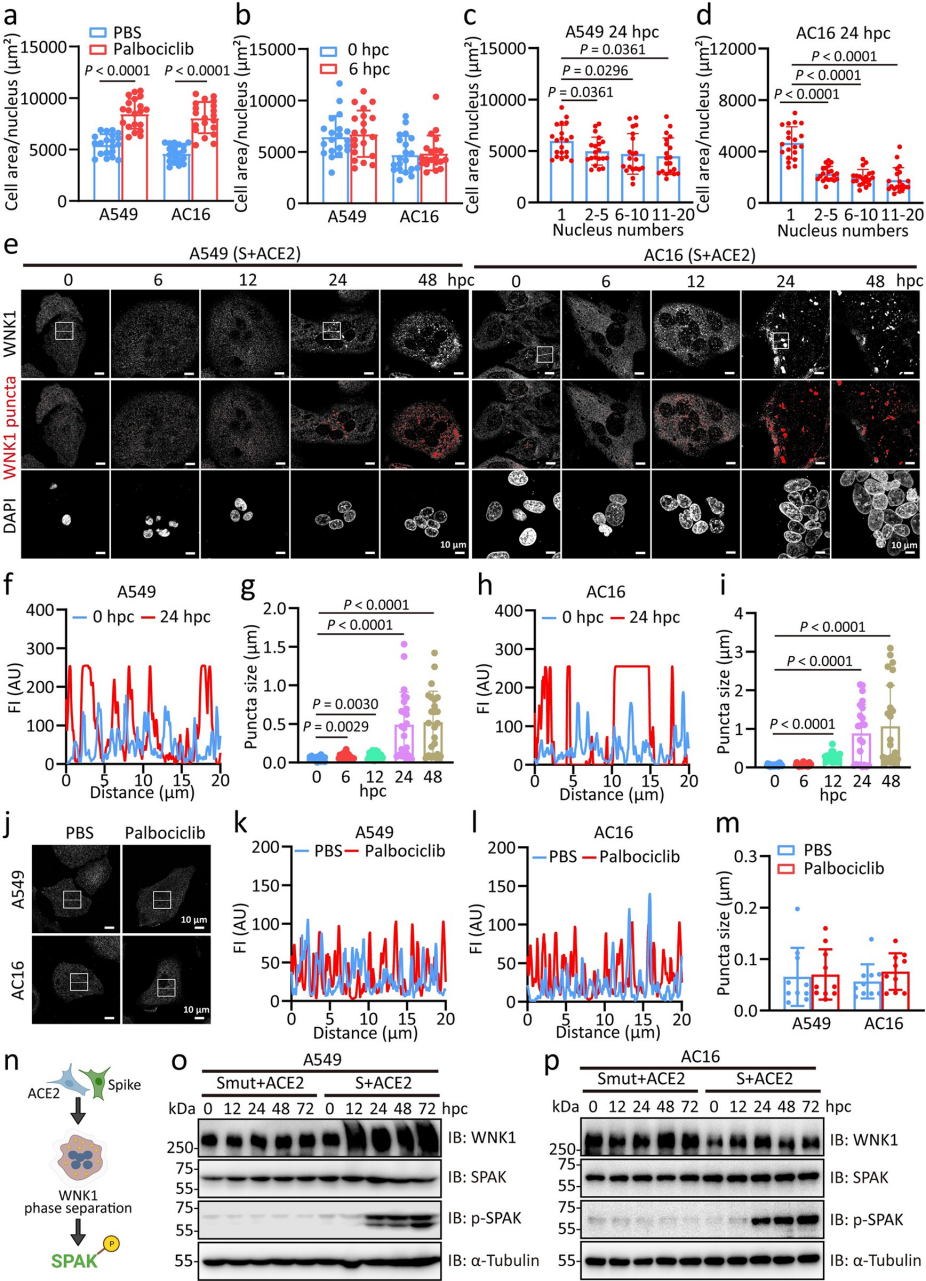
Senescent SARS-2-S syncytia shrinkage triggers WNK1 phase separation. a, Quantification of cell area per nucleus of A549 and AC16 cells treated with palbociclib (1 μM) for 7 days (n = 20 cells). b, Quantification of cell area per nucleus in cocultured A549 or AC16 cells for 6 hpc (n = 20 cells). c-d, Quantification of cell area per nucleus in cocultured A549 cells (c) or AC16 cells (d) with 1, 2–5, 6–10, and 11–20 nuclei numbers at 24 hpc (n = 20 cells). e, Confocal microscopic images of WNK1 in cocultured A549 or AC16 cells for the indicated times. White, WNK1 staining; red, WNK1 puncta. Scale bars represent 10 μm. f-g, Line scans represent fluorescence intensities (FIs) of the inset arrows (f) and quantification of average high-intensity puncta size/confocal field (n = 20 fields) (g) of A549 cells in e. h-i, Line scans represent FIs of the inset arrows (h) and quantification of average high-intensity puncta size/confocal field (n = 20 fields) (i) of AC16 cells in e. The high-intensity threshold signal was kept constant across all conditions analysed. j, Confocal microscopic images of WNK1 in A549 or AC16 cells treated with PBS or palbociclib (1 μM) for 7 days. White, WNK1 staining. k-l, Line scans represent FIs of the inset arrows in j from A549 (k) or AC16 (l) cells. The high-intensity thresholded signal was kept constant across all conditions analysed. m, Quantification of average high-intensity puncta size/confocal field in j (n = 10 fields). n, Scheme of phosphorylation-dependent regulation of the SPAK by WNK1. o-p, Immunoblotting analysis of WNK1, SPAK, and SPAK phosphorylation in S or S_NF_ cocultured A549 (o) or AC16 (p) cells for the indicated times. α-Tubulin was used as the loading control. All images are representative of 3 biological replicates. All quantified data are presented as the mean ± SD of independent experiments. Statistical significance was determined using one-way ANOVA and Bonferroni’s post hoc analysis (a, b, c, d, h, i, m).

A defining feature of cellular senescence is the enlargement and vacuolization of the cell body [40]. Here in our assay, A549 and AC16 cells treated with senescence stressor palbociclib for 7 days, these two types of cells exhibited enlarged morphology compared with cells treated with PBS (Figs 5A and S7A). However, we noticed distinct morphological changes of A549 and AC16 cells during SARS-2-S-triggered fusion progression at 6 hpc (Fig 5B). SARS-2-S syncytia of A549 cells began to exhibit a smaller surface area per nucleus regardless of the nuclei numbers with a smaller size tendency at 24 hpc (Fig 5C). The disproportionate decrease in cell volume per single nucleus was also consistently evident in senescent cardiomyocyte AC16 syncytia (Fig 5D). Importantly, these SA-β-gal-positive syncytia were alive for at least 7 observational days after cocultured SARS-2-S-expressing AC16 cells with ACE2-expressing AC16 cells or cocultured SARS-2-S-expressing AC16 cells with AC16 cells without overexpression of ACE2 (S7B and S7D Fig), with increased transcription of the *TNF*, *IL6*, *IL8* and *CDKN1A* genes at 48hpc (S7C Fig). Previous study indicated that with-no-lysine (WNK) kinase1 is a molecular crowding sensor that undergoes cell shrinkage-dependent phase separation to restore cell volume, the shrinkage of senescent SARS-2-S syncytia made us wonder whether WNK1 was activated [41]. Notably, we observed a significant shift of WNK1 from a diffuse to punctate distribution in senescent SARS-2-S syncytia of A549 and AC16 cells (Fig 5E–5I), but not in those cells treated with palbociclib (Fig 5J–5M). This phase behaviour appeared as early as 12 hpc in AC16 cells (Fig 5E). Accordingly, WNK1-triggered SPAK phosphorylation began to appear at 24 hpc and persisted for 72 hpc (Fig 5N–5P). No signs of SPAK phosphorylation signals were observed in SARS-2-S_NF_ cocultured cells (Fig 5O and 5P). Therefore, senescent SARS-2-S syncytia exhibit shrinked morphology, leading to the activation of WNK1.

### WNK1 activation in senescent SARS-2-S syncytia impairs cardiac metabolism

**Fig 6:**
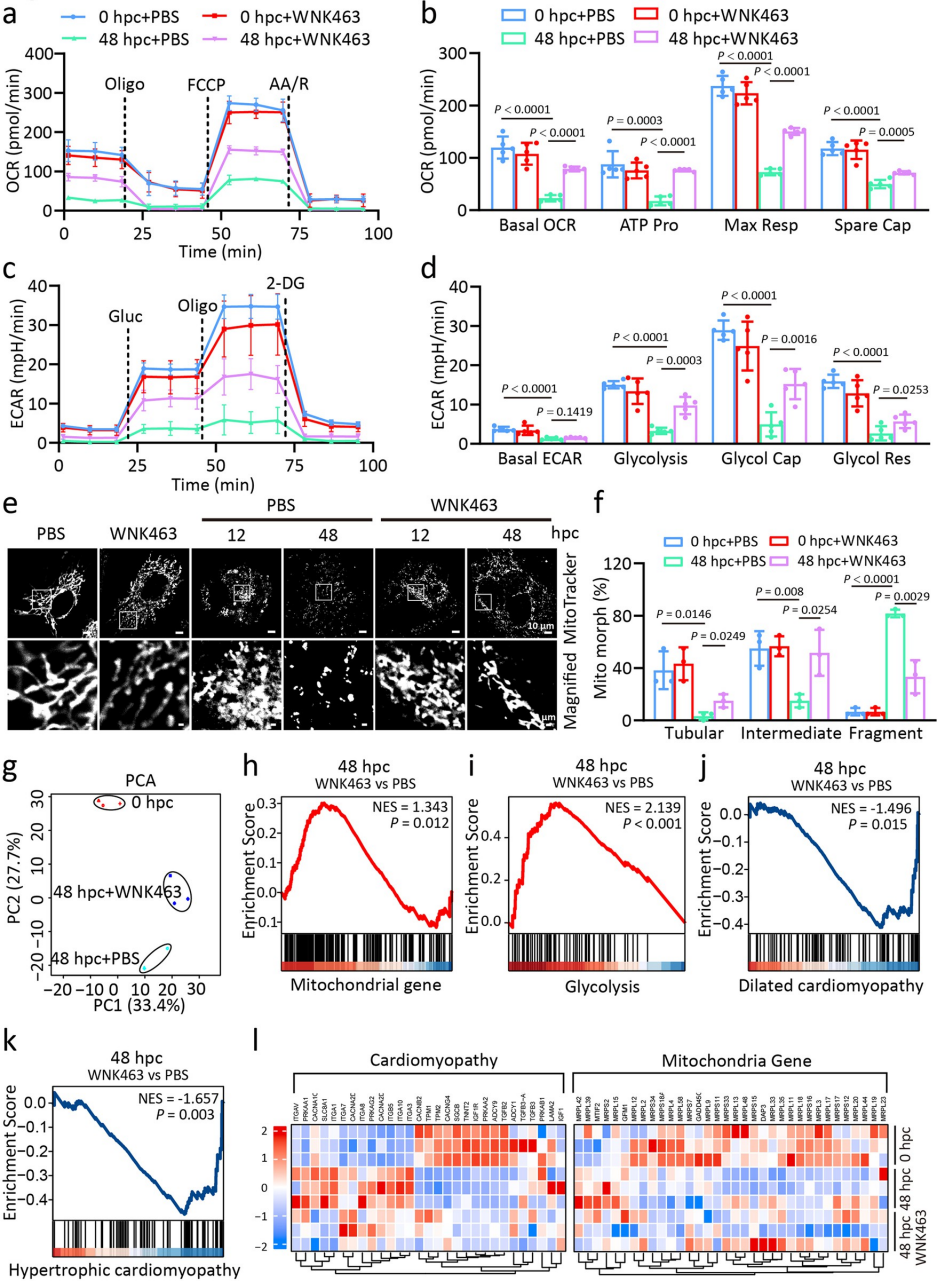
WNK1 activation in senescent SARS-2-S syncytia impairs cardiac metabolism. a-d, Seahorse analysis of OCR and ECAR in AC16 cocultured cells at 48 hpc in the presence or absence of WNK463 (1μM). OCR was measured with sequential addition of oligomycin (Oligo, 1.5μM), FCCP (2μM), and AA/R (1μM) (a) and the mitochondrial bioenergetic parameters including basal OCR, ATP production (ATP Pro), max respiration (Max Res), and spare capability (Spare Cap) were calculated (b). ECAR was measured with serial addition of addition of glucose (Gluc, 10 mM), oligo (1 μM), and 2-deoxyglucose (2-DG, 0.1 mM) (c) and the glycolytic parameters including basal ECAR, glycolysis, glycolysis capacity (Glyco Cap) and glycolytic reserve (Glyco Res) were calculated (d). e,f, Confocal microscopic images (e) and the quantification (f) of the mitochondrial morphology (Mito morph) in cocultured AC16 cells at 48 hpc in the presence or absence of WNK463 (1μM). Red, MitoTracker staining for mitochondria. Tubular: >10 μm length; Intermediate: ≤10 μm; Fragment: spherical (no clear length or width). Scale bars represent 10 μm (whole image) and 1 μm (magnified). g, PCA of gene expression patterns in cocultured AC16 cells at 48 hpc in the presence or absence of WNK463. In all plots, each point represented an independent biological replicate. h-k, GSEA of genes related to the mitochondrial (h), Glycolysis (i), dilated cardiomyopathy (j), and hypertrophic cardiomyopathy (k) signalling pathway among the DEGs in g. l, Heatmap of the critical DEGs in the cardiomyopathy and mitochondrial signalling pathway in cocultured AC16 cells at 48 hpc in the presence or absence of WNK463. All quantified data are shown as the mean ± SD of 3 independent experiments. All images are representative of 3 biological replicates. Statistical significance was determined using one-way ANOVA and Bonferroni’s post hoc analysis (b, d, e). OCR: oxygen consumption rate, ECAR: extracellular acidification rate.

Previous study indicated that hypochloremia could result in WNK1 activation and subsequently modulate RV metabolism via glucose handling and AMPK activation, small molecule inhibition of WNK1 (WNK463) signaling activates AMPK signaling and mitigates membrane enrichment of glucose transporters 1 and 4, which decreases protein O-GlcNAcylation and glycation [42]. Quantitative proteomics of right ventricular (RV) mitochondrial enrichments showed WNK463 prevents down-regulation of several mitochondrial metabolic enzymes [42]. So, we try to study energy metabolism of cardiomyocytes syncytia whether dependent on WNK1 activation [42,43]. We next explored the possibility that senescent SARS-2-S syncytia in cardiomyocytes with WNK1 activation might contribute to impaired metabolism in the setting of heart dysfunction. Compared to non-fusion controls, the mitochondrial oxidative capacity of the cocultured cardiomyocytes was significantly decreased at 48 hpc as indicated by reduced basal respiration, ATP-linked respiration, spare respiratory, and maximal respiration (Fig 6A and 6B). In addition, these senescent cells had more fragmented mitochondria with deteriorated glucose glycolysis capacity (Fig 6C–6F). Remarkably, small molecular inhibition of WNK1 via WNK463 was sufficient to rescue impaired mitochondrial respiration, mitochondrial integrity, and glycolytic metabolism (Fig 6A–6F).

To further elucidate the mechanisms underlying the role of activated WNK1 in cardiac metabolism, we performed RNA-seq to profile the transcriptomes in syncytial cardiomyocytes with or without WNK463 challenge, using nonfusion cells as a control. PCA revealed that WNK463 shifted the transcript signature towards nonfusion controls (Fig 6G). KEGG enrichment analysis showed the enrichment of hypertrophic cardiomyopathy and dilated cardiomyopathy in cocultured cardiomyocytes at 48 hpc (S8 Fig). Remarkably, WNK463 treatment led to a significant enrichment of upregulated genes in mitochondrial integrity and glycolysis (Fig 6H, 6I and 6L), whereas genes in cardiomyopathy were consistently enriched in downregulated DEGs (Fig 6J–6L). Taken together, we demonstrated that WNK1 activation in SARS-2-S syncytia significantly altered the heart transcriptomes and contributes to heart dysfunction by impairing cardiac metabolism.

### Senescent SARS-2-S syncytia exacerbated heart failure

**Fig 7:**
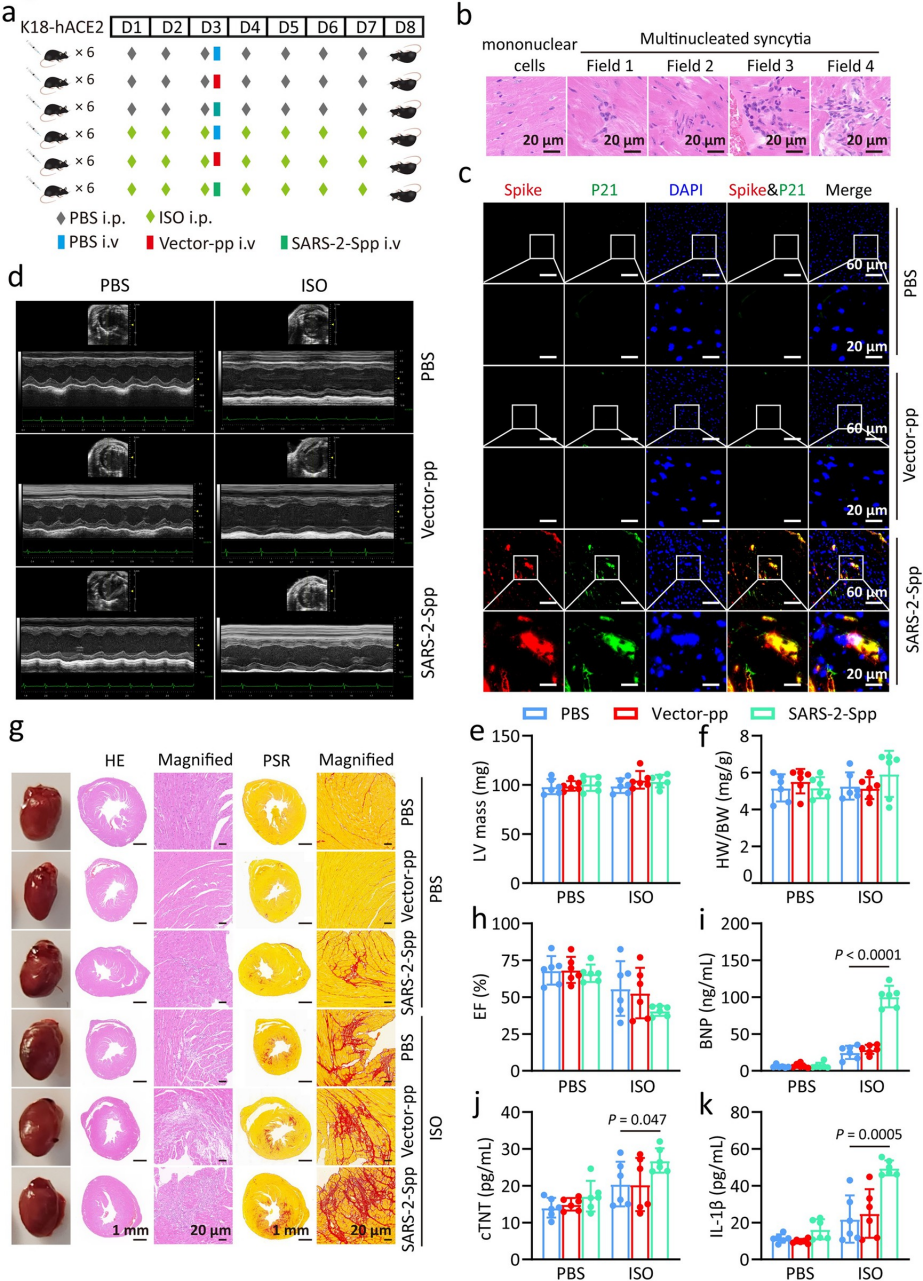
Senescent SARS-2-S syncytia exacerbated heart failure. a, Overview of the SARS-2-Spp-infected mouse model. ISO was subcutaneously injected into the backs of K18-hACE2 mice. Two days after ISO injection, 100 μL PBS, Vector-pp (2.5X10^8^ TU/mL), or SARS-2-Spp (2.5X10^8^ TU/mL) was injected daily by tail vein daily continuing for 5 days. Cardiac function was monitored via echocardiography 5 days after SARS-2-Spp injection. Following the functional assessment, mice were euthanized, and their hearts were collected for histopathological analysis. b, The histological analysis of the hearts of mice injected with SARS-2-Spp revealed the presence of multinucleated syncytia. Scale bars, 20 μm as indicated. c, Immunofluorescence staining of murine cardiac tissue following injection with PBS, Vector-pp, or SARS-2-Spp. Red, Spike staining; green, P21 staining; blue, DAPI staining. d, Representative M-mode echocardiographic images from mice in a. e-f, Left ventricular (LV) mass (e) and HW/BW ratio (f) in mice treated with PBS, Vector-pp, or SARS-2-Spp. g, Representative images showing whole hearts, cross-sections with HE staining and PSR staining. Red, PSR staining for fibrotic areas. Scale bars, 1mm or 20 μm as indicated. h, Ejection fraction (EF) from mice in a. i-k, Serum BNP (i), cTnT (j), and IL-1β (k) levels from mice in a. All quantified data are shown as the mean ± SD of n = 6 independent experiments. All images are representative of 3 biological replicates. Statistical significance was determined using one-way ANOVA and Bonferroni’s post hoc analysis (e, f, h, i, j, k,). BNP: Brain natriuretic peptide. Fig 7A created with Biorender.com.

To assess whether senescent SARS-2-S syncytium stimulates heart dysfunction *in vivo*, K18-hACE2 mice were administered with pseudovirus expressing BA.5 spike (SARS-2-Spp) intravenously (Fig 7A). Mice were euthanized on the fifth day post administered with SARS-2-Spp. Hematoxylin and eosin (HE) staining was performed to detect syncytia formation, and fluorescence staining was performed to detect cardiomyocyte senescence. The results showed that the presence of cardiomyocyte syncytia in the hearts of mice infected with the SARS-2-Spp (Fig 7B). Additionally, fluorescence staining showed that cellular senescence in syncytia expressing the S protein (Fig 7C). On the eighth day post administered, mice were performed heart function tests, then euthanized for cardiac pathology tests (Fig 7D). No significant differences were observed in LV mass or the heart-to-body weight (HW/BW) ratio between the PBS-, vector-pp and SARS-2-Spp-treated groups (Fig 7E and 7F). HE and picrosirius red (PSR) staining of the myocardium showed that the extent of fibrosis was more profound in SARS-2-Spp-treated mice compared to PBS- or vector-pp treated groups (Figs 7G and S9H). Functional analysis of hearts, including ejection fraction (EF), fractional shortening (FS), left ventricular internal dimension at end diastole (LVID;d), left ventricular internal dimension at end-systole (LVID;s), as well as serum brain natriuretic peptide (BNP) levels between the two groups were also comparable (Figs 7H, 7I and S9A–S9H). Thus, SARS-2-S promotes fibrosis without causing baseline cardiac abnormalities during 7 observational days. We then repeated the above experiment with intraperitoneally (i.p.) injected isoproterenol (ISO) to assess the impact of SARS-2-Spp on cardiac function in mice with pre-existing heart failure. As expected, acute overstimulation with ISO significantly reduced the cardiac function and promoted the myocardial fibrosis (Fig 7D and 7G). However, LV mass and HW/BW ratio were not affected (Fig 7E and 7F). Strikingly, SARS-2-Spp treatment in ISO mice led to severe fibrosis and reduced heart function as compared to PBS-treatment in ISO mice (Fig 7D and 7G). Notably, SARS-2-Spp also induced a significant increase in serum levels of BNP, troponin T (cTnT), and IL-1β (Fig 7I–7K). Thus, SARS-2-Spp treatment exacerbated heart failure progression in pre-existing heart failure mice.

### WNK1 inhibition, eliminate senescent cells and inhibit fusion effectively protects the heart from exacerbated heart failure triggered by SARS-2-S

**Fig 8:**
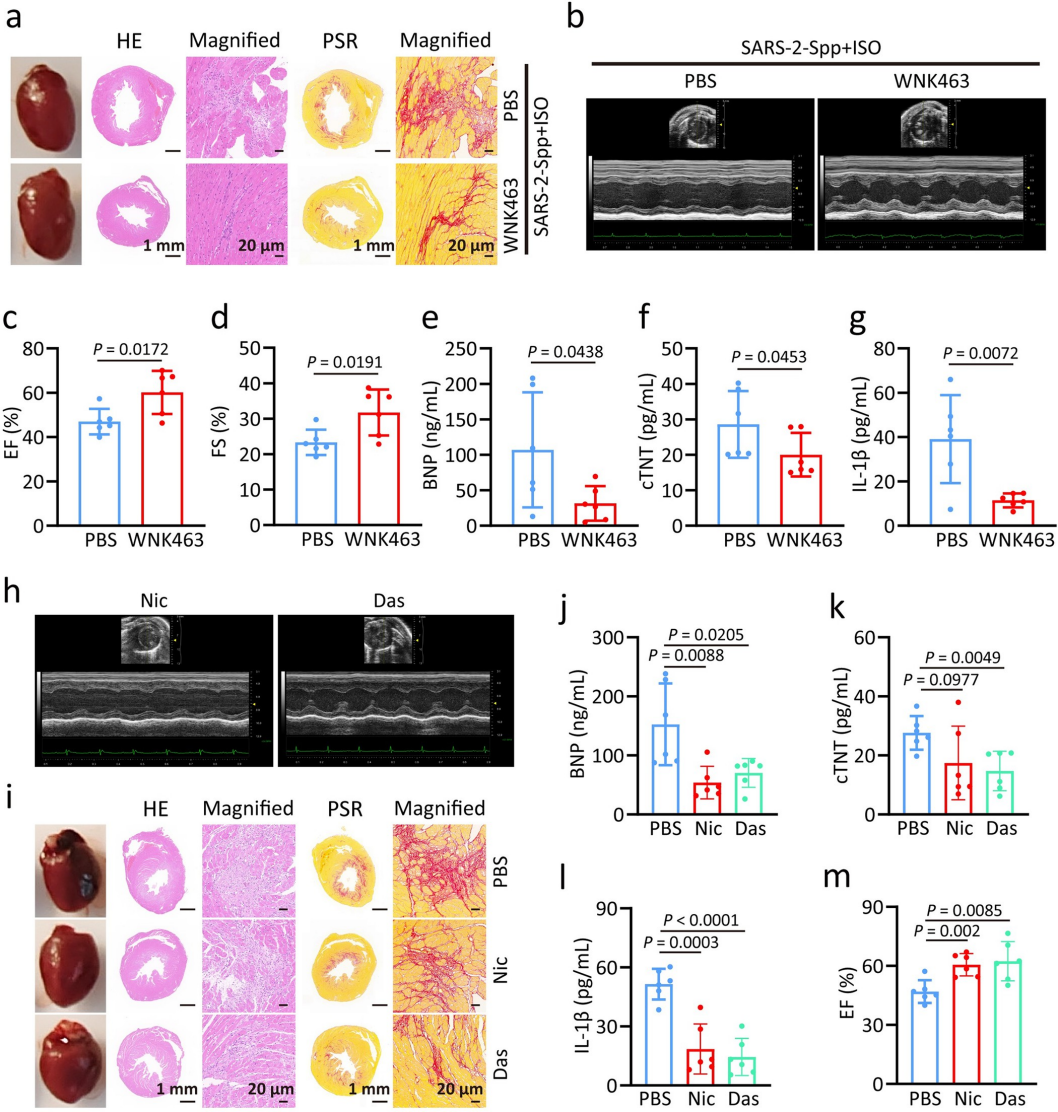
WNK1 inhibition effectively protects the heart from exacerbated heart failure triggered by SARS-2-S a, Representative images showing cross-sections with HE staining and PSR staining from SARS-2-Spp-infected ISO mice treated with PBS or WNK463. b-l, Representative M-mode echocardiographic images (b), EF (c), FS (d), serum BNP (e), cTnT (f), and IL-1β (g) levels from mice in a. h-m, Cardiac function and pathology from SARS-2-Spp-infected ISO mice treated with niclosamide or dasatinib, mice received Niclosamide (100 mg/kg) was given by oral gavage 1 day before SARS-2-Spp injection, dasatinib (5 mg/kg) was given by oral gavage daily 1 day after SARS-2-Spp injection. Representative M-mode echocardiographic images (h) demonstrate cardiac function. Representative images showing whole hearts, cross-sections with HE staining and PSR staining(i), scale bars, 1mm or 20 μm as indicated. Serum BNP (j), cTnT (k), and IL-1β (l) levels are shown as indicators of cardiac injury and inflammation. m, EF from SARS-2-Spp-infected ISO mice treated with PBS, niclosamide (Nic) or dasatinib (Das). All quantified data are shown as the mean ± SD of n = 6 independent experiments. Statistical significance was determined using two-tailed Student’s t test (c, d, e, f, g, j, k, l, m).

We then wanted to test if WNK1 inhibition by WNK463 can alleviate heart failure exacerbated by SARS-2-S through rescuing metabolic dysfunction. Strikingly, mice treated with WNK463 showed improved heart function as determined by enhanced EF, FS, and reduced serum BNP levels (Figs 8A–8E and S9I). WNK463 exposure also led to significantly reduced serum levels of cTNT and IL-1β (Fig 8F and 8G). Therefore, WNK1 intervention can effectively protect the heart from exacerbated heart failure triggered by SARS-2-S. Additional, to further prove that senescence SARS-CoV-2 syncytia act as a central pathogenic principle provoking heart failure, we attempted to eliminate senescent cells or inhibit fusion by using the senolytic drug dasatinib or the anti-syncytial drug niclosamide. Intriguingly, the administration of dasatinib and niclosamide substantially alleviated the heart dysfunction and fibrosis severity in ISO-treated K18-ACE2 BALB/c mice challenged with SARS-2-Spp (Figs 8H, 8I, 8M and S9J–S9Q). We detected significantly lower concentrations of BNP, cTNT, and IL-1β in the serum of mice treated with dasatinib and niclosamide (Fig 8J–8L), further supporting the notion that senescent SARS-2-S syncytia contribute to exacerbated heart failure.

## Discussion

As the main surface-exposed antigen and mediator of cell entry, the SARS-2-S protein mediates fusion between the viral and cellular membranes during particle entry, fusion with uninfected cells and FFWO^14,15^. However, the pathological consequences of this fusion and the regulation of fused cells remain poorly defined. Here, we directly linked SARS-2-S-triggered syncytium formation with the ensuing induction of cellular senescence and its pathophysiological contribution to heart failure progression. We found that both SARS-2-S expression and SARS-2-S protein internalization were sufficient to induce senescence in non-senescent ACE2-expressing cells. In particular, mRNAs-SARS-2-S injection provoked pathways enriched in senescence and SASP phenomenon *in vivo*. This was not only important for acute SARS-CoV-2 infection, but the persistence of circulating SARS-2-S protein or circulating extracellular vesicles containing SARS-2-S (S-EVs) in PASCs increase the likelihood of cell-cell fusion in uninfected cells. Mechanistically, MAVS regulated the anti-death to senescence fate of SARS-2-S syncytia through the TNFα-TNFR2 axis. SARS-2-S senescent syncytia with a smaller size tendency triggered WNK1 activation and led to impaired cardiac metabolism. Senescence induction by SARS-2-S depends on its fusogenic ability as the SARS-2-SRA mutant was unable to induce fusion and failed to induce senescence and WNK1 activation. Notably, pre-existing heart failure was significantly exaggerated by SARS-2-Spp in K18-hACE2 mice. *In vivo* testing of senolytic, WNK1 inhibitor, and anti-syncytia drug implicated senescence syncytia as a central driver in promoting heart failure progression, highlighting the clinical potential of WNK1 inhibitors as a novel interventional therapeutic strategy.

Activation of DNA sensors by DNA chromatin has been illustrated in promoting paracrine senescence and the production of SASP in VIS [44]. Here, we show that RIG-I-MAVS signalling was activated and governed the senescence fate of SARS-2-S syncytia. While we cannot definitively exclude the possible involvement of chromatin DNA and its sensors cGAS and STING in activating the senescence response induced by SARS-2-S syncytia, the *in vitro* knockdown experiment clearly demonstrates the crucial role of RIG-I-MAVS, but not STING, in this process. The existence and sources of endogenous dsRNA warrant further investigation. Nevertheless, we provide evidence of the contribution of RIG-I-MAVS to the regulation of SARS-2-S syncytium fate through TNFα. Using multiplex protein analysis, we identified a burst of SASP-related cytokines, including TNFα, IL-6, IFN-γ, and others, in the hACE2 mouse model injected with SARS-2-S mRNA. Although the exact contribution of each cytokine to SARS-2-S syncytium survival and senescence remains unclear, we provide evidence that the central regulation of SARS-2-S syncytium survival and senescence may be dependent on TNFα. Previous studies reported that TNFR2 interacts with the transmembrane form of TNFα (mTNFα) upon cell-to-cell contact [45–47]. Indeed, TNFR2 siRNA effectively reduced the increased survival of cocultured cells induced by TNFα, highlighting the indispensable role of TNFα/mTNFα in governing the fate of SARS-2-S syncytia mainly by TNFR2.

Our results have also revealed that the S sequence derived from the COVID-19 mRNA vaccine which harbors mutations in two proline residues. These mutations effectively inhibit the formation of syncytia, mitigate cellular senescence, and prevent the release of SASP. This finding corroborates prior research, affirming that the unique design of the mRNA vaccine mitigates syncytia formation, thus enhancing its safety. Moreover, our findings emphasize the necessity of optimizing vaccine sequences to mitigate the risk of syncytia formation in vaccine design.

With the progression of COVID-19, heart failure appears to be a potential hazard due to SARS-CoV-2 infection, chiefly in elderly patients with hypertension, ischaemic heart disease, and diabetes mellitus [48]. Patients with basic heart failure disease showed elevated ACE2 expression [49], whereas patients with PACS exhibited persistent circulating SARS-2-S and EVs harbouring SARS-2-S [16,17]. High ACE2 expression and long SARS-2-S duration may increase the likelihood of syncytium formation. Moreover, the pre-existing inflammatory environment with older age or with chronic disease, especially those with high TNFα levels, may increase the survival and senescence of syncytia. We therefore proposed that factors including pre-existing inflammation, high ACE2 expression, and persistent SARS-2-S could contribute to cardiac or multi-organ damage during different stages of SARS-CoV-2 infection by increasing the burden of senescent syncytia. In addition, vaccines using recombinant SARS-2-S fragments or other derivatives of SARS-2-S that are not fusogenic were reported to have fewer complications than vaccines that express fully or partially fusogenic SARS-2-S. S It remains to be demonstrated whether the senescent syncytia were induced in cell autonomous manner or through paracrine effect and its pathological consequences observed in assay systems are of relevance for clinical heart failure progression.

A defining feature of cellular senescence is characterized by its distinct morphological changes during progression [40]. Here, we found that SARS-2-S senescent syncytia exhibited a smaller surface area per nucleus. As a molecular crowding sensor that undergoes cell shrinkage-dependent phase separation to restore cell volume, WNK1 phase separation was identified in SARS-2-S syncytia. It has also been reported that TNFα activates the WNK1-SPAK-NCC phosphorylation cascade and contributes to salt-sensitive hypertension in several CKD mouse models [50]. Therefore, the effect of TNFα on WNK1 activation should be carefully monitored later. Previous studies have shown that small-molecule inhibition of WNK1 prevents the downregulation of mitochondrial metabolism and improves RV function and survival in pulmonary arterial hypertension [42]. We found that WNK1 inhibition alleviated SARS-2-S-aggravated heart failure and reduced serum cTnT and IL-1β levels. cTnT is released into circulation during injury to cardiac myocytes[51]. Individuals with higher cTnT levels are at an increased risk of developing heart failure and sudden cardiac death [52]. Therefore, through its metabolism-rescue and anti-inflammatory properties, WNK463 may have dual protective effects in preventing severe myocardial lesions and heart failure in “high-risk” patients. As TNFα and anti-syncytial drug acts in the early phase of cell-cell fusion, its narrow time window may limit its therapeutic use. While senescence elimination reduces hypertrophy and improves function, how the heart responds to senolytic treatment in longer-term studies has yet to be investigated. Here, we recommend that rescue of cardiac metabolism dysfunction should be taken into consideration in individuals during the acute or post-acute stage of SARS-CoV-2 infection. It should be noted that SARS-CoV-2 infection also induces new onset of multiple cardiovascular diseases and causes long-term cardiovascular sequelae in PACS patients [53]. Cytokine storm was suggested to be a point of convergence in the pathophysiologic process between COVID-19 and heart failure [54]. Here we found that SARS-2-S led to profound cardiac fibrosis without affecting baseline cardiac function. Further studies examining the chronic pathological role of SARS-2-S senescent syncytia are needed to determine whether WNK inhibitors are beneficial in preventing the new onset of cardiovascular diseases or alleviating long-term cardiovascular sequelae in PACS patients.

In summary, we provide evidence showing direct induction of cell-cell fusion with a senescence-like phenotype in FFWI and FFWO manners by SARS-2-S both *in vitro* and *in vivo*. The susceptibility of patients with heart failure to more pronounced complications during the post-acute stages of SARS-CoV-2 infection could be related to the senescent outcome of SARS-2-S syncytia. WNK1 may be a druggable target in the management of heart failure associated with long COVID-19.

## Materials and methods

### Ethics statement

All work involving SARS-CoV-2 was approved by the Institutional Biosafety Committee and performed in the BSL-3 facility. All animal experiments were carried out at the Animal Laboratory of the Laboratory Animal Center, Academy of Military Medical Sciences, under the ethical approval number IACUC-DWZX-2023-P023.

### Reagents

Dulbecco’s modified Eagle’s medium (DMEM, high glucose, D5796) and protease inhibitor cocktail I (20–201) were purchased from Millipore (Massachusetts, USA). Foetal bovine serum (FBS, A3160901) was purchased from Gibco (New York, USA). Lipofectamine 2000 (11668–027), MitoTracker Deep Red FM and CellEvent Senescence Green detection kits (C10850) were purchased from Invitrogen (Massachusetts, USA). BCA protein concentration determination kit (P0012), DAPI (C1002) and β-galactosidase reporter gene assay kit (RG0036) were purchased from Beyotime (Shanghai, China). NucleoZOL (740404) was purchased from MACHEREY-NAGEL (MN, Düren, Deutschland). PerfectStart Green qPCR SuperMix (AQ601) and TransScript One-Step gDNA Removal and cDNA Synthesis SuperMix (AT311) were purchased from TransGen Biotech (Beijing, China). WNK463 (S8358), fisetin (S2298), dasatinib (S1021) and venetoclax (S8048) were purchased from Selleck Chemicals (Houston, TX). Recombinant human IL-6 (AF-200-06) and human TNFα (300-01A) were purchased from PeproTech (New Jersey, USA). Mouse cTnT/TNNT2 ELISA Kit (E-EL-M1801c), and Mouse BNP ELISA Kit (E-EL-M0204c) were purchased from Elabscience (Wuhan, China). Mouse 1L-1β Precoated ELISA Kit (1210122) was purchased from Dakewe Biotech (Beijing, China). The SARS-CoV-2 spike pseudotyped lentivirus (P231127-1014rfz) for *in vivo* experiments was purchased from VectorBuilder (Guangzhou, China).

### Antibodies

Anti-CD81 (ab109201, 1:1,000 dilution), anti-CD63 (ab134045, 1:1,000 dilution), anti-TSG101 (ab125011, 1:1,000 dilution), anti-CD9 (ab263019, 1:1,000 dilution), anti-ACE2 (ab108252, 1:1,000 dilution), anti-WNK1 (ab174854, 1:2,000 dilution), anti-SPAK (ab128894, 1:2,000 dilution) and anti-IRF3 (ab76409, 1:1000 dilution) were purchased from Abcam (Cambridge, UK). Anti-p-SPAK ser373, anti-α-tubulin (T9026, 1:5,000 dilution) and anti-Flag (A8592, 1:10,000 dilution) were purchased from Sigma-Aldrich (Missouri, USA). Anti-TNFα (60291-1-Ig, 1:5,000 dilution), anti-MAVS (14341-1-AP, 1:100 dilution for immunofluorescence, 1:4,000 dilution for immunoblotting), anti-TRAF3 (18099-1-AP, 1:1,000 dilution), anti-p21 (10355-1-AP, 1:100 dilution), anti-p16 (10883-1-AP, 1:100 dilution), anti-TBK1 (28397-1-AP, 1:2,000 dilution), anti-STING (19851-1-AP, 1:3,000 dilution), CoraLite 488-conjugated TNFα antibody (CL488-60291, 1:200 dilution), anti-TNFR2 (28746-1-AP, 1:200 dilution) and anti-GAPDH (60004-4-I-Ig, 1:5,000 dilution) were purchased from Proteintech (Wuhan, China). Anti-SARS-CoV/SARS-2-S (GTX632604, 1:2,000 dilution) was purchased from GeneTex (CA, USA). Anti-TRAF6 (8028S, 1:1,000 dilution), anti-phospho-p65 (3033T, 1:1,000 dilution), anti-RIG-I (3743T, 1:1,000 dilution), anti-phospho-IRF3 (4947S, 1:1,000 dilution) and anti-phospho-TBK1 (5483T, 1:2,000 dilution) were purchased from Cell Signaling Technology (Massachusetts, USA). Anti-phospho-p65 (A19653, 1:1,000 dilution) was purchased from ABclonal (Wuhan, China). Anti-rabbit HRP-IgG (ZB-2301, 1:5,000 dilution) and anti-mouse HRP-IgG (ZB-2305, 1:5,000 dilution) were purchased from ZSGB-BIO (Beijing, China).

### Plasmids, cell culture and small interfering RNAs (siRNAs)

Mammalian expression vectors encoding SARS-2-S (Wuhan-Hu-1, GenBank: NC_045512.2), SARS-2-S_NF_ (spike R682A, R683A, R685A, K814A and R815A) and SARS-2-S_O_ (Omicron) were codon-optimized and cloned into the pcDNA3.1 expression vector. To produce lentivirus plasmids, SARS-2-S and ACE2 were cloned into the pCDH-CMV-MCS-EF1-Puro vector. NF-κB-Luc, IFN-β-Luc and IRF-3-Luc reporter gene constructs were kept in our laboratory. All plasmids were verified by DNA sequencing.

The A549 (CRL-185), HEK293T (293T, CRL-3216), HeLa (CRL-3216), and HIEC-6 (CRL-3266) cell lines were obtained from the American Type Culture Collection (ATCC, Rockville, MD, USA). The AC16 (SCC109) cell line was obtained from Sigma-Aldrich (Missouri, USA). MAVS-KO 293T cells were kindly provided by Prof. T. Li (National Center of Biomedical Analysis, Beijing, China). All cell lines were mycoplasma free and incubated in DMEM supplemented with 10% FBS, 100 U/mL penicillin, and 0.1 mg/mL streptomycin at 37°C under a humidified atmosphere with 5% CO2. Lipofectamine 2000 was used for transfection following the manufacturer’s protocol. For lentivirus production, pMD2. G, psPAX2 and pCDH-SARS-2-S or pCDH-ACE2 were transfected into 293T cells together, and lentiviral particles were harvested from cell supernatants filtered through a 0.45 μm filter 48 h later. For stable overexpression of ACE2 and SARS-2-S, 293T, A549, AC16, HIEC-6, and HeLa cells were infected with harvested lentiviral particles and selected in the presence of puromycin (2–4 μg/mL). Stably transfected clones were selected under puromycin pressure after two weeks. To knock down specific genes, siRNAs targeting human genes were purchased from GenePharma, and their target sequences are listed in S1 Table.

### Cell-cell fusion assay

Cells were stably transfected with SARS-2-S or ACE2 and cultured to 60% confluence. Then, SARS-2-S-expressing cells and ACE2-expressing cells were either mixed at 1:1 ratios or allowed to continue culture. At the indicated times, the cocultured cells were harvested, and the individually cultured SARS-2-S-expressing cells and ACE2-expressing cells were mixed *in vitro* using the same ratio used as the nonfusion controls.

### Quantification of fusion level based on β-galactosidase reporter gene assay

To quantify the cell-cell fusion level, an α-complementation of the lacZ system based on β-galactosidase was used [14]. Producer cells were constructed with SARS-2-S and lacZ (△11–41 aa), and target cells were constructed with ACE2 and lacZ (1–56 aa). Upon cell-cell fusion, complementation of lacZ (△11–41 aa) with lacZ (1–56 aa) inside the syncytium results in the formation of active β-galactosidase, which is then quantified by a β-galactosidase Reporter Gene Assay Kit according to the manufacturer’s instructions. Briefly, 50 μL of lysate collected from the syncytium at the indicated times was treated with equal amounts of the specific substrate 2-nitrophenyl β-D-galactopyranoside. After 3 h of incubation, the reactions were quenched with 150 μL stop solution, and the absorbance was measured at 420 nm using a multimode microplate reader (Tecan).

### SARS-2-S-mRNA-LNP synthesis

T7 RNA polymerase-mediated transcription from a linearized DNA template from a plasmid that encodes codon-optimized SARS-2-S was used to produce mRNA *in vitro*. LNP formulations were prepared using a method previously described for siRNA with modifications[55]. Briefly, lipids were dissolved in ethanol containing an ionizable lipid, 1,2-distearoyl-sn-glycero3-phosphocholine, cholesterol and PEG-lipid (at molar ratios of 50:10:38.5:1.5). The lipid mixture was combined with 20 mM citrate buffer (pH 4.0) containing mRNA at a ratio of 1:2 through a T-mixer. The formulations were then diafiltrated against a 10 × volume of PBS (pH 7.4) through a tangential-flow filtration membrane with a 100 kDa molecular weight cut-off (Sartorius Stedim Biotech), concentrated to the desired concentrations, and passed through a 0.22 μm filter. All formulations were tested for particle size, distribution, RNA concentration and encapsulation.

### EV purification

For EV production, supernatants of HeLa and SARS-2-S stably transfected HeLa cells cultured in EV-depleted medium were collected after 72 h and centrifuged at 300 × g for 10 min to remove the cells. The cell supernatant was diluted 1:2 with cold PBS before low-speed centrifugation at 3000 × g for 10 min, and the resulting supernatant was transferred to new tubes for ultracentrifugation at 100,000 ×g using a type 100 TI rotor in an XPN-100 instrument (CP100NX; Hitachi, Brea, USA) for 90 min. The resulting EV pellet was resuspended in 250 mL of cold PBS and denoted EVs. EVs concentration and quantity were determined using NTA with a NanoSight NS300 instrument (Malvern Panalytical).

### Flow cytometry

To assess the cellular uptake of S-EVs, the cells were exposed to DiO-labelled EVs at the indicated doses and then incubated at 37°C for the indicated durations. After extensive washing, flow cytometry was performed with an Attune NxT instrument (Thermo Fisher Scientific, Massachusetts, USA), and 30,000 cell acquisitions under a medium flow rate were used for data capture. Data analysis was performed using FlowJo software (V10.0.7r2). A control sample consisting of DiO dye used as the background was prepared following the same procedure indicated above.

For SARS-2-S protein expression detection, cells were treated with S-EVs. Forty hours later, the cells were detached and incubated with SARS-2-S mAb (GTX632604, clone 1A9, 1:1,000 dilution) for 30 min on ice, followed by incubation with PE goat anti-mouse IgG (Biolegend, 405307, 1:100 dilution) for 15 min at room temperature. The cells were then analysed by flow cytometry.

### Multiplex bead-based protein detection (MAGPIX)

Multiplex bead-based protein analyses were conducted according to the manufacturer’s instructions with a custom Procartaplex 36-plex panel (Thermo Fisher Scientific, cat no. EPX360-26092-901). Briefly, samples were diluted, and 50 μL of each prepared sample was incubated with antibody-conjugated magnetic beads for 1 h overnight at 4°C. After washing, detection antibodies were added and incubated at room temperature for 30 min. Following further washing steps, PE-streptavidin was added and incubated for 30 min. The assay data were measured with the Bio-Plex MAGPIX multiplex reader (Bio-Rad) and analysed with a 5PL algorithm provided by Bio-Plex Manager software (version 6.1).

### Semidenaturing detergent agarose gel electrophoresis (SDD-AGE)

Crude mitochondria and cytosolic extracts were separated from cells using differential centrifugation with a cell mitochondria isolation kit (Beyotime, C3601) according to a published protocol [56]. A549 cells were resuspended and lysed by grinding and were centrifuged at 800 × g for 10 min at 4°C. The supernatants were transferred to a new tube and centrifuged at 13,000 × g for 10 min at 4°C to separate the crude mitochondria and cytosolic extracts. The crude mitochondria were resuspended in sample buffer (0.5 × TAE, 10% glycerol, 2% SDS, and 0.0025% bromophenol blue) and loaded onto a 1.5% agarose gel. Then, the samples were electrophoresed at a constant voltage of 80 V at 4°C. The proteins were transferred to PVDF membranes for immunoblotting.

### Luciferase reporter assays

A luciferase expression vector controlled by a promoter containing three repeats of the NF-κB response element was obtained from Beyotime (D2204). The pSV40-Renilla plasmid was purchased from Promega (E2231). To assess the activity of NF-κB, cells were plated in triplicate in a 24-well plate and then transiently transfected with pNF-κB-luc and the internal control plasmid pSV40-Renilla for 12 h. The cells were then cocultured for the indicated durations. The cells were harvested, and firefly luciferase activity and Renilla luciferase activity were detected using the dual-luciferase reporter assay system (Promega, E1910) with a multimode microplate reader (Tecan). The firefly luciferase luminescence data were normalized to the Renilla luciferase luminescence data.

### VSV infection

Cells were plated in 12-well plates and VSV was added to the medium at a concentration of 80 haemagglutinating units per mL and a multiplicity of infection (MOI) of 5 at 60% cell confluence. After 1 h of incubation, the extracellular virus was removed by washing the cells twice with serum-containing medium. Cells were fixed at the indicated times after infection for immunofluorescence assays.

### Syncytial viability assay

A CCK8 assay was used to assess syncytial cell viability. Briefly, cocultured cells were seeded into a 96-well plate at a cell density of 3000 per well and allowed to adhere for 72 h. Then, the medium was washed and replaced with fresh medium, followed by treatment with serially diluted inhibitors. At 24 h after treatment, 10 μL of CCK-8 solution was added to 100 μL of culture medium, and the absorbance of each well was measured at 450 nm using a multimode microplate reader (Tecan) with Tecan Spark Control (v.2.1) software. All experiments were performed in triplicate, and the cell viability was calculated as the ratio of the viability under each experimental condition to the viability of the control.

### Animals

Male C57BL/6J WT mice (8-week-old) were purchased from SPF Biotechnology (Beijing, China). The K18-hACE2 transgenic mice, in which the human keratin 18 (KRT18) promoter was used to direct human ACE2 expression, were purchased from Cyagen. All mice were conventionally group-housed on a 12-h light/dark cycle in an animal facility for 3 days before any procedures.

For S-mRNA-LNP injections, we injected 3.0 x 10^11^ μg AAV-hACE2 constructs into the tail veins of 8-week-old C57BL/6J mice to drive mainly hepatic hACE2 expression. Four weeks later, the mice were administered 3.0 μg of S-mRNA-LNP or LNP via i.m. injection, followed by terminal sacrifice of the mice and the collection of sera and livers at the indicated times.

For ISO-induced heart failure model, 12-week-old K18-hACE2 C57BL/6J male mice was subcutaneously injected with ISO (5 mg/kg) into the backs daily continuing for 7 days. Two days after ISO injection, the mice were injected with 100 μL PBS, Vector-pp (2.5×10^8 TU/mL), or SARS-2-Spp (2.5X10^8^ TU/mL) by tail vein for 5 days. For drug testing, niclosamide (100 mg/kg) was given by oral gavage 1 day before SARS-2-Spp injection. Dasatinib (5 mg/kg) was given by oral gavage daily 1 day after SARS-2-Spp injection. WNK463 (2.5 mg/kg) was given intraperitoneally daily 1 day after SARS-2-Spp injection. Five days after SARS-2-Spp injection, the mice were anaesthetized with isoflurane and cardiac function was analyzed by a Vevo2100 digital high-frequency ultrasound system (FUJIFILM VisualSonics, Inc). Mice were then sacrificed, serum and the organs were collected for future use. The heart images were collected using a Tanon 2500B imaging system (Tanon, Shanghai, China), with the same parameter settings and shooting distance. All animal studies were performed at the Academy of Military Medical Sciences (AMMS) Animal Centre according to standard operating procedures in a specific pathogen-free facility under the approval of the Institutional Animal Care and Use Committee (approved animal protocol number IACUC-DWZX-2023-P023).

### Histological staining and immunofluorescence

Tissue samples were fixed in 4% paraformaldehyde, embedded in paraffin, and sectioned at 5 μm thickness. For histological analysis, sections were deparaffinized in xylene and rehydrated through a graded alcohol series. Hematoxylin and eosin (H&E) staining was performed to assess general tissue morphology. For the detection of collagen fibers, Sirius Red staining was applied. After deparaffinization and hydration, sections were stained with 0.1% Sirius Red in saturated picric acid for one hour, followed by two washes in acidified water to remove unbound dye.

Immunofluorescence was conducted to localize the Spike protein, P21 expression, and nuclear DNA. Following antigen retrieval with citrate buffer (pH 6.0), sections were permeabilized with 0.1% Triton X-100 in PBS and blocked in 5% BSA to prevent non-specific binding. The sections were then incubated with primary antibodies against the SARS-COV-2 Spike protein and P21 overnight at 4°C. After washing with PBS, sections were incubated with appropriate fluorescently labeled secondary antibodies. Nuclear counterstaining was performed using DAPI. Slides were mounted with an antifade mounting medium and visualized using a fluorescence microscope.

### Quantitative real-time PCR (RT-qPCR)

Total mRNA was extracted from cells or tissues using NucleoZOL. cDNA was prepared from total mRNA by using TransScript One-Step gDNA Removal and cDNA Synthesis SuperMix, and the relative amounts of individual mRNAs were calculated after normalization to the corresponding β-actin mRNA. The primer sequences are included in S2 Table.

### Western blotting and immunoprecipitation

Cells were lysed in NP40 cell lysis buffer with fresh protease inhibitors. Supernatants were separated by SDS-PAGE after centrifugation and transferred to PVDF membranes for immunoblot analyses using the indicated antibodies.

Whole-cell lysates for coimmunoprecipitation were prepared by sonication in NP40 buffer (1% NP40, 150 mM NaCl and 40 mM Tris pH 7.5). Clarified lysates were then incubated with the indicated antibodies and with Protein A/G PLUS-agarose. After four washes with PBS, the immunoprecipitates were collected by centrifugation and reserved for immunoblot analyses.

### RNA-sequencing (RNA-seq) analysis

Total RNA was isolated from cells or liver tissues with TRIzol reagent, and the RIN was checked to inspect RNA integrity with an Agilent 2100 Bioanalyzer (Agilent Technologies, Santa Clara, CA, US). The qualified total RNA was further purified with an RNAClean XP kit (cat No. A63987, Beckman Coulter, Inc., Kraemer Boulevard, Brea, CA, USA) and RNase-Free DNase Set (cat# 79254, QIAGEN, GmBH, Germany). cDNA libraries were constructed following Illumina standard protocols and sequenced with an Illumina NovaSeq 6000 by SHBIO (Shanghai, China). The sequencing reads were mapped to the human genome using TopHat (version 1.0.13). Avadis NGS (version 1.3) was used to calculate reads per kilobase per million mapped reads (RPKM) values. The P value and fold change (FC) in the expression of genes between groups were jointly used to identify differentially expressed genes (DEGs). The criteria for statistical significance were P< 0.05 and |Log2(FC)|>1. GO enrichment and KEGG pathway analyses were conducted by SHBIO to investigate the potential functions of the DEGs, and P< 0.05 was considered to indicate a statistically significant difference.

### Immunofluorescence analysis

For immunofluorescence staining, cells or tissue sections were fixed with a 4% paraformaldehyde solution, washed twice with PBS and permeabilized with 0.3% Triton X-100 in PBS. The Triton X-100 solution was replaced with 5% (w/v) BSA, and samples were incubated with primary antibodies for 1 h. After washing three times with PBST (0.05% Tween 20 in PBS), the samples were incubated with fluorophore-conjugated secondary antibodies diluted in PBS for 30 min. After washing three times with PBST, the nuclei were labelled with a DAPI solution. For dsRNA staining assays, J2 was used as the primary antibody, and Alexa Fluor 555-labelled donkey anti-mouse IgG (H+L) secondary antibody was used as the secondary antibody.

For SA-β-gal staining assays, SA-β-gal was detected with the CellEvent Senescence Green Probe under a fluorescence microscope. Fluorescence images were acquired under a Nikon A1 confocal microscope, Zeiss LSM710 confocal microscope or Tecan Spark Cyto Imaging system. The images were processed and analysed using Volocity (v.6.1.1, PerkinElmer), Nikon NIS-Elements AR (v4.00.12) or ImageJ (version 1.53c).

### Seahorse assay

The Seahorse XFe24 Extracellular Flux Analyzer and Seahorse XF Glycolytic Rate Assay Kit from Agilent Technologies were used to measure ECAR and OCR according to the manufacturer’s instructions. In brief, cells were seeded in XFe24 microplates and incubated overnight at 37°C in a CO2 incubator. On the day of the assay, the cells were washed and incubated with Seahorse assay medium. OCR was measured in response to oligomycin (1.5 μM), FCCP (2 μM), and antimycin A /rotenone (AA/R, 1 μM), while ECAR was measured in response to glucose (10 mM), oligomycin (1 μM), and 2-deoxyglucose (2-DG, 0.1 mM). The results were analysed using Wave software (Seahorse/Agilent).

### Statistical analysis

In the present study, GraphPad Prism 8.0 was used for statistical calculations and data plotting. Differences between two independent samples were evaluated by two-tailed Student’s t tests or the Mann-Whitney test, as appropriate. Differences between multiple samples were analysed by one-way ANOVA or two-way ANOVA, followed by Bonferroni’s post hoc analysis, as appropriate. All tests were two-tailed unless otherwise indicated. We considered a P value<0.05 to indicate statistical significance. Significance values were set as follows: ns (not significant), P > 0.05; *, P < 0.05; **, P < 0.01; ***, P < 0.001.

## Supporting information

S1 Fig SARS-2-S syncytia exhibit a senescence-like phenotype. a-b, Immunoblot analysis of SARS-2-S (a) and ACE2 (b) expression in A549, 293T, HeLa and HIEC-6 cells transfected with the indicated plasmids. α-Tubulin was used as a loading control. c, Immunoblot analysis of A549 cells transfected with S, SNF, SBA.1, SBA.5, SBA.2.75 or SBA2.75.2. α-Tubulin was used as a loading control. d, SA-β-gal staining of A549-eLV, A549-S or A549-ACE2 co-cultured with A549-eLV cells for the indicated times. Green, SA-β-gal staining; blue, nuclear DAPI staining. Scale bars represent 100 μm. e-f, SA-β-gal staining (e), fluorescent SA-β-gal staining and bright field imaging (f) of A549-S+A549-ACE2 or A549-SNF+A549-ACE2 cells for the indicated times. Green, SA-β-gal staining; blue, nuclear DAPI staining. Scale bars represent 100 μm. g, Normalized expression of CDKN1A, TNF, IL6 and IL8 transcripts in Ctrl, SNF, S, ACE2, S+ACE2 and SNF+ACE2 cells at 48 hpc by RT-qPCR. h-i, Quantification of the fusion of A549-S+A549-ACE2 (h) and A549-SNF+ A549-ACE2 (i) based on the β-galactosidase α-complementation assay for the indicated times. j, Quantification of the relative fusion ability of four Omicron variants based on the β-galactosidase α-complementation assay. The fusion level of A549-S and A549-ACE2 cocultured cells at 24 hpc was set to 1. k, Cell viability of cocultured A549-S+A549-ACE2 cells and A549-SNF+ A549-ACE2 cells treated with fisetin, dasatinib, or venetoclax for 48 h by CCK8 assay. All images are representative of 3 biological replicates. All quantified data are presented as the mean ± SD of n = 3 independent experiments. Statistical significance was determined with two-tailed Student’s t test (g, h, i, j).
(TIF)

S2 Fig SARS-2-S delivered by EVs triggers syncytial senescence. a, Illustration of EV-mediated FFWO. Cells were treated with S-EVs for 24 h, followed by coculture with ACE2-expressing cells for the indicated times. A mixture of A549-EVs and A549-ACE2 cells was used as a nonfusion control. b, Immunoblot analysis of EV markers (CD9, CD63, CD81, and TSG101) and S expression in EVs purified from the supernatant (SN) of HeLa or HeLa-S cells. c, Nanoparticle Tracking Analysis (NTA) analysis of EVs and S-EVs. d, Analysis of DiO-labelled S-EVs or EVs uptake by A549 cells. Green, DiO-labelled EVs; red, S staining; blue, nuclear DAPI staining. e, Flow cytometry analysis of S expression on the cell surface of A549-S-EVs cells. f-g, SA-β-gal staining of A549-S-EVs cocultured with 293T-ACE2 cells (f) or EVs-treated A549-ACE2 cells (g) for the indicated times. Green, SA-β-gal staining; blue, nuclei DAPI staining. Scale bars represent 100 μm. h, SA-β-gal staining of EVs- or S-EVs-treated A549 cells for the indicated times. Green, SA-β-gal staining; blue, nuclei DAPI staining. Scale bars represent 100 μm. i-j, Quantification of the fusion degree of FFWO-1 (i) and FFWO-2 (j) based on the β-galactosidase α-complementation assay for the indicated times. All the images are representative of three independent experiments. All quantified data are presented as the mean ± SD of n = 3 independent experiments. Statistical significance was determined with two-tailed Student’s t test (i, j). S2A Fig created with Biorender.com.
(TIF)

S3 Fig SARS-2-S delivered by mRNAs triggers syncytial senescence. a, Illustration of the cell coculture system mediated by S-mRNAs and S-BNT162b2. Cells were treated with S-mRNA-LNP or S-BNT162b2 for 24 h, followed by coculture with ACE2-expressing cells for the indicated times. A mixture of A549-LNP+A549-ACE2 and A549-Luc-mRNA-LNP+ A549-ACE2 cells were used as a nonfusion control. b, Immunoblot analysis of S expression in A549 cells treated with LNP, Luc-mRNA-LNP, S-mRNA-LNP and S-BNT162b2. α-Tubulin was used as a loading control. c, SA-β-gal and p21 staining of A549-S-EVs and A549-ACE2 cocultured cells for the indicated times. Green, SA-β-gal staining; red, p21 staining; blue, DAPI staining. Scale bars represent 100 μm. d, Quantification of LNP, Luc-mRNA-LNP, S-mRNA-LNP and S-BNT162b2 induced fusion degree based on the β-galactosidase α-complementation assay at 24h. e, Quantification of S-mRNA-LNP induced fusion degree based on the β-galactosidase α-complementation assay for the indicated times. f-h, ACE2 expression or S expression (h) in livers from mice injected with S-mRNA-LNPs or LNPs by immunoblotting (f) or immunohistochemical staining (g). i, Pathways enriched in DEGs identified in the livers of mice injected with LNPs or S-mRNA-LNPs according to KEGG functional pathways at level 2. The bar plot shows significantly dysregulated pathways (P < 0.05), with Fisher’s exact test P values shown on the x-axis. All images are representative of three independent experiments. All quantified data are presented as the mean ± SD of n = 3 independent experiments. Statistical significance was determined with two-tailed Student’s t test (d, e). S3A Fig created with Biorender.com.
(TIF)

S4 Fig SARS-2-S syncytia induce the formation of functional MAVS aggregates dependent on RIG-I. a, Confocal microscopic images of MAVS and mitochondria in cocultured A549 cells at 4 hpc. Red, MitoTracker staining for mitochondria; green, MAVS staining; blue, DAPI staining. A magnified view of the boxed region is shown in the middle panel, and a 3D reconstruction of the Z stacks is shown in the right panel. Scale bars represent 10 μm (whole image) and 1 μm (magnified). b, Knockdown efficiency of siMAVS, siRIG-I, siMFN1, or siOPA1 compared to siCtrl by RT-qPCR. c, Relative luciferase activity of NF-κB in cocultured A549 cells for the indicated times. Luciferase activity was normalized to the values at 0 hpc. All quantified data in this figure are shown as the means ± SDs of n = 3 independent experiments. Statistical significance was determined with two-tailed Student’s t test (b), one-way ANOVA and Bonferroni’s post hoc analysis (c).
(TIF)

S5 Fig MAVS-TNFα regulates the survival and senescence of SARS-2-S syncytia. a, b, Twenty most significantly enriched pathways in cocultured A549 cells at 4 hpc (a) and 48 hpc (b) according to KEGG pathway analysis. The enriched terms are shown on the y-axis, and the P values (log transformed) assessing significant enrichment are shown on the x-axis with Fisher’s exact test. The enrichment degree of KEGG was measured by enrichment factors (rich factor), P value and the number of genes enriched in this pathway.
(TIF)

S6 Fig MAVS-TNFα regulates the survival of SARS-2-S syncytia. a, b, Relative viability of WT-S, WT-ACE2, MAVS-KO-S, and MAVS-KO-ACE2 cells treated with IL-6 (a) or TNFα (b) at the indicated concentrations. The cell viability of cells treated with PBS was set to 100%. c, Heatmap of the critical DEGs of the TNF signalling pathway in cocultured A549 cells at 4 hpc and 48 hpc. d, Relative viability of A549-SNF and A549-ACE2 cells co-cultured for 24 hours following siRNA-mediated knockdown of TNFRSF1A(TNFR1), TNFRSF1B(TNFR2), MAVS, RIG-I, and STING. The cell viability of cells treated with siCtrl was set to 100%. e, Relative fusion of siTNFR1 and siTNFR2 cocultured A549 cells at 24 hpc by β-galactosidase assay. The fusion level of siCtrl cocultured cells at 24 hpc was set to 1. f-j, Knockdown efficiency of three siRNA sequences targeting MAVS(f), RIG-I(g), STING(h), TNFRSF1A (i), TNFRSF1B (j), as compared to siRNA control (siCtrl) assessed by Western Blot (WB) analysis. All quantified data are shown as the means ± SDs of n = 3 independent experiments. Statistical significance was determined with two-tailed Student’s t test (d), one-way ANOVA and Bonferroni’s post hoc analysis (a, b, d, e).
(TIF)

S7 Fig Senescent SARS-2-S syncytia shrinkage triggers WNK1 phase-separation. a, SA-β-gal staining of A549 and AC16 cells following treatment with PBS and Palbociclib for 7 days. Scale bars represent 100 μm. b, SA-β-gal staining of AC16-S and AC16-ACE2 cells cocultured for the indicated days (dpc). Scale bars represent 100 μm. Green, SA-β-gal staining; blue, nuclear DAPI staining. c, Normalized expression of TNF, IL6, IL8, and CDKN1A in AC16 cells at 48 hpc from a relative to that at 0 hpc by RT-qPCR. d, SA-β-gal staining of AC16-S and AC16 cells cocultured for the indicated days (dpc). Scale bars represent 100 μm. Green, SA-β-gal staining; blue, nuclear DAPI staining. All quantified data are shown as the mean ± SD of n = 3 independent experiments. Statistical significance was determined using two-tailed Student’s t test (c).
(TIF)

S8 Fig WNK1 activation in senescent SARS-2-S syncytia impairs cardiac metabolism. Twenty most significantly enriched pathways in cocultured AC16 cells at 48 hpc versus 0 hpc according to KEGG pathway analysis. The enriched terms are shown on the y-axis, and the P values (log transformed) assessing significant enrichment are shown on the x-axis with Fisher’s exact test. The enrichment degree of KEGG was measured by enrichment factors (rich factor), P value and the number of genes enriched in this pathway.
(TIF)

S9 Fig Senescent SARS-2-S syncytia exacerbated heart failure. a-g, Fractional shortening (FS) (a), LV end-systolic volumes (LV Vol;d) (b), end-diastolic volumes (LV Vol; s) (c), left ventricular end-diastolic anterior wall thickness (LVAW;d) (d), left ventricular posterior wall thickness (LVPW;d) (e), left ventricular end-diastolic diameter (LVID;d) (f), and left ventricular end-systolic diameter (LVID;s) (g) of PBS, Vector-pp or SARS-2-Spp-infected mice with or without ISO-injection. h, Quantification of the percentage red area in PSR stained cardiac sections from PBS, Vector-pp, or SARS-2-Spp-infected mice, with or without ISO injection, indicating the extent of fibrosis. i, Quantification of the percentage red area in PSR stained cardiac sections from SARS-2-Spp-infected ISO mice treated with PBS or WNK463. j-p, FS (j), LV Vol;d (k), LV Vol; s (l), LVAW;d (m), LVPW;d (n), LVID;d (o), and LVID;s (p) of SARS-2-Spp-infected ISO-mice treated with PBS, niclosamide (Nic), or dasatinib (Das). q, Quantification of the percentage red area in PSR stained cardiac sections from SARS-2-Spp-infected ISO-mice treated with PBS, niclosamide (Nic), or dasatinib (Das). All quantified data are presented as the mean ± SD of n = 6 independent experiments. Statistical significance was determined with one-way ANOVA and Bonferroni’s post hoc analysis (a, b, c, d, e, f, g, h, i, j, k, l, m, n, o, p, q).
(TIF)

S10 Fig SARS-CoV-2 spike-induced syncytia are senescent and contribute to exacerbated heart failure. SARS-2-S induced syncytia via FFWI and FFWO manner exhibits a senescence-like phenotype regardless of the cell type or syncytium nucleus number. Mechanistically, RIG-I-MAVS drives the TNFα-dependent survival and senescence fate of SARS-2-S syncytia. The susceptibility of patients with heart failure to more pronounced complications during the acute and post-acute stages of SARS-CoV-2 infection could be related to the senescent outcome of SARS-2-S syncytia, and WNK1 inhibitor may be a druggable target in the management of heart failure associated with SARS-CoV-2 infection. S10 Fig created with Biorender.com.
(TIF)

S1 Table List of siRNA sequences. (PDF)

S2 Table List of Primer sequences. (PDF)

S1 Data List of uncropped images. (PDF)

## References

[1] Paci E, Bisanti L. [The COVID-19 epidemic and total mortality: missed data and the incoming epidemiology]. Epidemiol Prev. 2023;47(1–2):67–72. Epub 2023/04/20. doi: 10.19191/EP23.1-2.A571.023 . 37078199

[2] Sherif ZA, Gomez CR, Connors TJ, Henrich TJ, Reeves WB, Force RMPT. Pathogenic mechanisms of post-acute sequelae of SARS-CoV-2 infection (PASC). Elife. 2023;12. Epub 2023/03/23. doi: 10.7554/eLife.86002 ; PubMed Central PMCID: PMC10032659. 36947108 PMC10032659

[3] Munipalli B, Seim L, Dawson NL, Knight D, Dabrh AMA. Post-acute sequelae of COVID-19 (PASC): a meta-narrative review of pathophysiology, prevalence, and management. SN Compr Clin Med. 2022;4(1):90. Epub 2022/04/12. doi: 10.1007/s42399-022-01167-4 ; PubMed Central PMCID: PMC8977184. 35402784 PMC8977184

[4] Proal AD, VanElzakker MB. Long COVID or Post-acute Sequelae of COVID-19 (PASC): An Overview of Biological Factors That May Contribute to Persistent Symptoms. Front Microbiol. 2021;12:698169. Epub 2021/07/13. doi: 10.3389/fmicb.2021.698169 ; PubMed Central PMCID: PMC8260991. 34248921 PMC8260991

[5] Xanthopoulos A, Bourazana A, Giamouzis G, Skoularigki E, Dimos A, Zagouras A, et al. COVID-19 and the heart. World J Clin Cases. 2022;10(28):9970–84. Epub 2022/10/18. doi: 10.12998/wjcc.v10.i28.9970 ; PubMed Central PMCID: PMC9561576. 36246800 PMC9561576

[6] Elseidy SA, Awad AK, Vorla M, Fatima A, Elbadawy MA, Mandal D, et al. Cardiovascular complications in the Post-Acute COVID-19 syndrome (PACS). Int J Cardiol Heart Vasc. 2022;40:101012. Epub 2022/04/01. doi: 10.1016/j.ijcha.2022.101012 ; PubMed Central PMCID: PMC8958273. 35355927 PMC8958273

[7] John KJ, Mishra AK, Ramasamy C, George AA, Selvaraj V, Lal A. Heart failure in COVID-19 patients: Critical care experience. World J Virol. 2022;11(1):1–19. Epub 2022/02/05. doi: 10.5501/wjv.v11.i1.1 ; PubMed Central PMCID: PMC8788216. 35117968 PMC8788216

[8] Hariri E, Patel NG, Bassil E, Matta M, Yu PC, Pack QR, et al. Acute but not chronic heart failure is associated with higher mortality among patients hospitalized with pneumonia: An analysis of a nationwide database(☆). Am J Med Open. 2022;7:100013. Epub 2022/06/24. doi: 10.1016/j.ajmo.2022.100013 ; PubMed Central PMCID: PMC9211036. 35734378 PMC9211036

[9] Sattar Y, Ullah W, Rauf H, Virk HUH, Yadav S, Chowdhury M, et al. COVID-19 cardiovascular epidemiology, cellular pathogenesis, clinical manifestations and management. Int J Cardiol Heart Vasc. 2020;29:100589. Epub 2020/07/30. doi: 10.1016/j.ijcha.2020.100589 ; PubMed Central PMCID: PMC7359794. 32724831 PMC7359794

[10] Viveiros A, Noyce RS, Gheblawi M, Colombo D, Bilawchuk LM, Clemente-Casares X, et al. SARS-CoV-2 infection downregulates myocardial ACE2 and potentiates cardiac inflammation in humans and hamsters. Am J Physiol Heart Circ Physiol. 2022;323(6):H1262–H9. Epub 2022/11/12. doi: 10.1152/ajpheart.00578.2022 ; PubMed Central PMCID: PMC9705018. 36367689 PMC9705018

[11] Yang Y, Wei Z, Xiong C, Qian H. Direct mechanisms of SARS-CoV-2-induced cardiomyocyte damage: an update. Virol J. 2022;19(1):108. Epub 2022/06/26. doi: 10.1186/s12985-022-01833-y ; PubMed Central PMCID: PMC9233758. 35752810 PMC9233758

[12] Rajah MM, Bernier A, Buchrieser J, Schwartz O. The Mechanism and Consequences of SARS-CoV-2 Spike-Mediated Fusion and Syncytia Formation. J Mol Biol. 2022;434(6):167280. Epub 2021/10/05. doi: 10.1016/j.jmb.2021.167280 ; PubMed Central PMCID: PMC8485708. 34606831 PMC8485708

[13] Buchrieser J, Dufloo J, Hubert M, Monel B, Planas D, Rajah MM, et al. Syncytia formation by SARS-CoV-2-infected cells. EMBO J. 2020;39(23):e106267. Epub 2020/10/15. doi: 10.15252/embj.2020106267 ; PubMed Central PMCID: PMC7646020. 33051876 PMC7646020

[14] Theuerkauf SA, Michels A, Riechert V, Maier TJ, Flory E, Cichutek K, et al. Quantitative assays reveal cell fusion at minimal levels of SARS-CoV-2 spike protein and fusion from without. iScience. 2021;24(3):102170. Epub 2021/02/16. doi: 10.1016/j.isci.2021.102170 ; PubMed Central PMCID: PMC7871100. 33585805 PMC7871100

[15] Bratt MA, Gallaher WR. Comparison of fusion from within and fusion from without by Newcastle disease virus. In Vitro. 1970;6(1):3–14. Epub 1970/07/01. doi: 10.1007/BF02616129 . 5535595

[16] Troyer Z, Alhusaini N, Tabler CO, Sweet T, de Carvalho KIL, Schlatzer DM, et al. Extracellular vesicles carry SARS-CoV-2 spike protein and serve as decoys for neutralizing antibodies. J Extracell Vesicles. 2021;10(8):e12112. Epub 2021/07/01. doi: 10.1002/jev2.12112 ; PubMed Central PMCID: PMC8213968. 34188786 PMC8213968

[17] Craddock V, Mahajan A, Spikes L, Krishnamachary B, Ram AK, Kumar A, et al. Persistent circulation of soluble and extracellular vesicle-linked Spike protein in individuals with postacute sequelae of COVID-19. J Med Virol. 2023;95(2):e28568. Epub 2023/02/10. doi: 10.1002/jmv.28568 ; PubMed Central PMCID: PMC10048846. 36756925 PMC10048846

[18] Schmitt CA, Tchkonia T, Niedernhofer LJ, Robbins PD, Kirkland JL, Lee S. COVID-19 and cellular senescence. Nat Rev Immunol. 2023;23(4):251–63. Epub 2022/10/06. doi: 10.1038/s41577-022-00785-2 ; PubMed Central PMCID: PMC9533263. 36198912 PMC9533263

[19] Lee S, Yu Y, Trimpert J, Benthani F, Mairhofer M, Richter-Pechanska P, et al. Virus-induced senescence is a driver and therapeutic target in COVID-19. Nature. 2021;599(7884):283–9. Epub 2021/09/14. doi: 10.1038/s41586-021-03995-1 . 34517409

[20] Campisi J, d’Adda di Fagagna F. Cellular senescence: when bad things happen to good cells. Nat Rev Mol Cell Biol. 2007;8(9):729–40. Epub 2007/08/02. doi: 10.1038/nrm2233 . 17667954

[21] Di Micco R, Krizhanovsky V, Baker D, d’Adda di Fagagna F. Cellular senescence in ageing: from mechanisms to therapeutic opportunities. Nat Rev Mol Cell Biol. 2021;22(2):75–95. Epub 2020/12/18. doi: 10.1038/s41580-020-00314-w ; PubMed Central PMCID: PMC8344376. 33328614 PMC8344376

[22] Kumari R, Jat P. Mechanisms of Cellular Senescence: Cell Cycle Arrest and Senescence Associated Secretory Phenotype. Front Cell Dev Biol. 2021;9:645593. Epub 2021/04/16. doi: 10.3389/fcell.2021.645593 ; PubMed Central PMCID: PMC8039141. 33855023 PMC8039141

[23] Tsuji S, Minami S, Hashimoto R, Konishi Y, Suzuki T, Kondo T, et al. SARS-CoV-2 infection triggers paracrine senescence and leads to a sustained senescence-associated inflammatory response. Nature Aging. 2022;2(2):115–24. doi: 10.1038/s43587-022-00170-7 37117754 PMC10154207

[24] Hoffmann M, Kleine-Weber H, Pohlmann S. A Multibasic Cleavage Site in the Spike Protein of SARS-CoV-2 Is Essential for Infection of Human Lung Cells. Mol Cell. 2020;78(4):779–84 e5. Epub 2020/05/05. doi: 10.1016/j.molcel.2020.04.022 ; PubMed Central PMCID: PMC7194065. 32362314 PMC7194065

[25] Hornich BF, Grosskopf AK, Schlagowski S, Tenbusch M, Kleine-Weber H, Neipel F, et al. SARS-CoV-2 and SARS-CoV Spike-Mediated Cell-Cell Fusion Differ in Their Requirements for Receptor Expression and Proteolytic Activation. J Virol. 2021;95(9). Epub 2021/02/21. doi: 10.1128/JVI.00002-21 ; PubMed Central PMCID: PMC8104116. 33608407 PMC8104116

[26] Suzuki R, Yamasoba D, Kimura I, Wang L, Kishimoto M, Ito J, et al. Attenuated fusogenicity and pathogenicity of SARS-CoV-2 Omicron variant. Nature. 2022;603(7902):700–5. Epub 2022/02/02. doi: 10.1038/s41586-022-04462-1 ; PubMed Central PMCID: PMC8942852. 35104835 PMC8942852

[27] Arora P, Nehlmeier I, Kempf A, Cossmann A, Schulz SR, Dopfer-Jablonka A, et al. Lung cell entry, cell-cell fusion capacity, and neutralisation sensitivity of omicron sublineage BA.2.75. Lancet Infect Dis. 2022;22(11):1537–8. Epub 2022/09/19. doi: 10.1016/S1473-3099(22)00591-6 ; PubMed Central PMCID: PMC9477470. 36116462 PMC9477470

[28] Saito A, Tamura T, Zahradnik J, Deguchi S, Tabata K, Anraku Y, et al. Virological characteristics of the SARS-CoV-2 Omicron BA.2.75 variant. Cell Host Microbe. 2022;30(11):1540–55 e15. Epub 2022/10/23. doi: 10.1016/j.chom.2022.10.003 ; PubMed Central PMCID: PMC9578327. 36272413 PMC9578327

[29] Nunez Lopez YO, Casu A, Pratley RE. Investigation of Extracellular Vesicles From SARS-CoV-2 Infected Specimens: A Safety Perspective. Front Immunol. 2021;12:617042. Epub 2021/05/11. doi: 10.3389/fimmu.2021.617042 ; PubMed Central PMCID: PMC8100059. 33968019 PMC8100059

[30] Pallesen J, Wang N, Corbett KS, Wrapp D, Kirchdoerfer RN, Turner HL, et al. Immunogenicity and structures of a rationally designed prefusion MERS-CoV spike antigen. Proc Natl Acad Sci U S A. 2017;114(35):E7348–E57. Epub 2017/08/16. doi: 10.1073/pnas.1707304114 ; PubMed Central PMCID: PMC5584442. 28807998 PMC5584442

[31] Hsieh CL, Goldsmith JA, Schaub JM, DiVenere AM, Kuo HC, Javanmardi K, et al. Structure-based design of prefusion-stabilized SARS-CoV-2 spikes. Science. 2020;369(6510):1501–5. Epub 2020/07/25. doi: 10.1126/science.abd0826 ; PubMed Central PMCID: PMC7402631. 32703906 PMC7402631

[32] Liu X, Wei L, Xu F, Zhao F, Huang Y, Fan Z, et al. SARS-CoV-2 spike protein-induced cell fusion activates the cGAS-STING pathway and the interferon response. Sci Signal. 2022;15(729):eabg8744. Epub 2022/04/13. doi: 10.1126/scisignal.abg8744 . 35412852

[33] Zhang Y, Yeruva L, Marinov A, Prantner D, Wyrick PB, Lupashin V, et al. The DNA sensor, cyclic GMP-AMP synthase, is essential for induction of IFN-beta during Chlamydia trachomatis infection. J Immunol. 2014;193(5):2394–404. Epub 2014/07/30. doi: 10.4049/jimmunol.1302718 ; PubMed Central PMCID: PMC4212656. 25070851 PMC4212656

[34] Rehwinkel J, Gack MU. RIG-I-like receptors: their regulation and roles in RNA sensing. Nat Rev Immunol. 2020;20(9):537–51. Epub 2020/03/24. doi: 10.1038/s41577-020-0288-3 ; PubMed Central PMCID: PMC7094958. 32203325 PMC7094958

[35] Jia Y, Song T, Wei C, Ni C, Zheng Z, Xu Q, et al. Negative regulation of MAVS-mediated innate immune response by PSMA7. J Immunol. 2009;183(7):4241–8. Epub 2009/09/08. doi: 10.4049/jimmunol.0901646 . 19734229

[36] Li D, Yang S, Xing Y, Pan L, Zhao R, Zhao Y, et al. Novel Insights and Current Evidence for Mechanisms of Atherosclerosis: Mitochondrial Dynamics as a Potential Therapeutic Target. Front Cell Dev Biol. 2021;9:673839. Epub 2021/07/27. doi: 10.3389/fcell.2021.673839 ; PubMed Central PMCID: PMC8293691. 34307357 PMC8293691

[37] Wajant H, Siegmund D. TNFR1 and TNFR2 in the Control of the Life and Death Balance of Macrophages. Front Cell Dev Biol. 2019;7:91. Epub 2019/06/14. doi: 10.3389/fcell.2019.00091 ; PubMed Central PMCID: PMC6548990. 31192209 PMC6548990

[38] Naude PJ, den Boer JA, Luiten PG, Eisel UL. Tumor necrosis factor receptor cross-talk. FEBS J. 2011;278(6):888–98. Epub 2011/01/15. doi: 10.1111/j.1742-4658.2011.08017.x . 21232019

[39] Holbrook J, Lara-Reyna S, Jarosz-Griffiths H, McDermott M. Tumour necrosis factor signalling in health and disease. F1000Res. 2019;8. Epub 2019/02/14. doi: 10.12688/f1000research.17023.1 ; PubMed Central PMCID: PMC6352924. 30755793 PMC6352924

[40] Herranz N, Gil J. Mechanisms and functions of cellular senescence. J Clin Invest. 2018;128(4):1238–46. Epub 2018/04/03. doi: 10.1172/JCI95148 ; PubMed Central PMCID: PMC5873888. 29608137 PMC5873888

[41] Boyd-Shiwarski CR, Shiwarski DJ, Griffiths SE, Beacham RT, Norrell L, Morrison DE, et al. WNK kinases sense molecular crowding and rescue cell volume via phase separation. Cell. 2022;185(24):4488–506 e20. Epub 2022/11/02. doi: 10.1016/j.cell.2022.09.042 ; PubMed Central PMCID: PMC9699283. 36318922 PMC9699283

[42] Prisco SZ, Eklund M, Raveendran R, Thenappan T, Prins KW. With No Lysine Kinase 1 Promotes Metabolic Derangements and RV Dysfunction in Pulmonary Arterial Hypertension. JACC Basic Transl Sci. 2021;6(11):834–50. Epub 2021/12/07. doi: 10.1016/j.jacbts.2021.09.004 ; PubMed Central PMCID: PMC8617575. 34869947 PMC8617575

[43] Bisaccia G, Ricci F, Gallina S, Di Baldassarre A, Ghinassi B. Mitochondrial Dysfunction and Heart Disease: Critical Appraisal of an Overlooked Association. Int J Mol Sci. 2021;22(2). Epub 2021/01/14. doi: 10.3390/ijms22020614 ; PubMed Central PMCID: PMC7827742. 33435429 PMC7827742

[44] Kohli J, Veenstra I, Demaria M. The struggle of a good friend getting old: cellular senescence in viral responses and therapy. EMBO Rep. 2021;22(4):e52243. Epub 2021/03/19. doi: 10.15252/embr.202052243 ; PubMed Central PMCID: PMC8024996. 33734564 PMC8024996

[45] Qu Y, Zhao G, Li H. Forward and Reverse Signaling Mediated by Transmembrane Tumor Necrosis Factor-Alpha and TNF Receptor 2: Potential Roles in an Immunosuppressive Tumor Microenvironment. Front Immunol. 2017;8:1675. Epub 2017/12/14. doi: 10.3389/fimmu.2017.01675 ; PubMed Central PMCID: PMC5712345. 29234328 PMC5712345

[46] Lind P, Langsteger W, Koltringer P, Eber B, Kammerhuber F, Smolle-Juttner F, et al. Localization of mediastinal parathyroid adenoma by T1-201 scintiscan and SPECT. Klin Wochenschr. 1990;68(9):472–5. Epub 1990/05/04. doi: 10.1007/BF01648902 . 2355726

[47] Horiuchi T, Mitoma H, Harashima S, Tsukamoto H, Shimoda T. Transmembrane TNF-alpha: structure, function and interaction with anti-TNF agents. Rheumatology (Oxford). 2010;49(7):1215–28. Epub 2010/03/03. doi: 10.1093/rheumatology/keq031 ; PubMed Central PMCID: PMC2886310. 20194223 PMC2886310

[48] Onohuean H, Al-Kuraishy HM, Al-Gareeb AI, Qusti S, Alshammari EM, Batiha GE. Covid-19 and development of heart failure: mystery and truth. Naunyn Schmiedebergs Arch Pharmacol. 2021;394(10):2013–21. Epub 2021/09/05. doi: 10.1007/s00210-021-02147-6 ; PubMed Central PMCID: PMC8417660. 34480616 PMC8417660

[49] Chen L, Li X, Chen M, Feng Y, Xiong C. The ACE2 expression in human heart indicates new potential mechanism of heart injury among patients infected with SARS-CoV-2. Cardiovasc Res. 2020;116(6):1097–100. Epub 2020/04/01. doi: 10.1093/cvr/cvaa078 ; PubMed Central PMCID: PMC7184507. 32227090 PMC7184507

[50] Furusho T, Sohara E, Mandai S, Kikuchi H, Takahashi N, Fujimaru T, et al. Renal TNFalpha activates the WNK phosphorylation cascade and contributes to salt-sensitive hypertension in chronic kidney disease. Kidney Int. 2020;97(4):713–27. Epub 2020/02/16. doi: 10.1016/j.kint.2019.11.021 . 32059997

[51] Mair J, Lindahl B, Hammarsten O, Muller C, Giannitsis E, Huber K, et al. How is cardiac troponin released from injured myocardium? Eur Heart J Acute Cardiovasc Care. 2018;7(6):553–60. Epub 2017/12/28. doi: 10.1177/2048872617748553 . 29278915

[52] Wettersten N, Maisel A. Role of Cardiac Troponin Levels in Acute Heart Failure. Card Fail Rev. 2015;1(2):102–6. Epub 2015/10/01. doi: 10.15420/cfr.2015.1.2.102 ; PubMed Central PMCID: PMC5491037. 28785441 PMC5491037

[53] Liuzzo G, Volpe M. SARS-CoV-2 infection markedly increases long-term cardiovascular risk. Eur Heart J. 2022;43(20):1899–900. Epub 2022/04/02. doi: 10.1093/eurheartj/ehac168 ; PubMed Central PMCID: PMC9383629. 35362024 PMC9383629

[54] Peng X, Wang Y, Xi X, Jia Y, Tian J, Yu B, et al. Promising Therapy for Heart Failure in Patients with Severe COVID-19: Calming the Cytokine Storm. Cardiovasc Drugs Ther. 2021;35(2):231–47. Epub 2021/01/07. doi: 10.1007/s10557-020-07120-8 ; PubMed Central PMCID: PMC7786163. 33404925 PMC7786163

[55] Ickenstein LM, Garidel P. Lipid-based nanoparticle formulations for small molecules and RNA drugs. Expert Opin Drug Deliv. 2019;16(11):1205–26. Epub 2019/09/19. doi: 10.1080/17425247.2019.1669558 . 31530041

[56] Xiong Y, Fang JH, Yun JP, Yang J, Zhang Y, Jia WH, et al. Effects of microRNA-29 on apoptosis, tumorigenicity, and prognosis of hepatocellular carcinoma. Hepatology. 2010;51(3):836–45. Epub 2009/12/31. doi: 10.1002/hep.23380 . 20041405

