# Supplementary figures and images for: SARS-CoV-2 spike-induced syncytia are senescent and contribute to exacerbated heart failure
Source: PLoS Pathog. 2024 Aug 5;20(8):e1012291. doi: 10.1371/journal.ppat.1012291 (PMC11326701; doi:10.1371/journal.ppat.1012291)

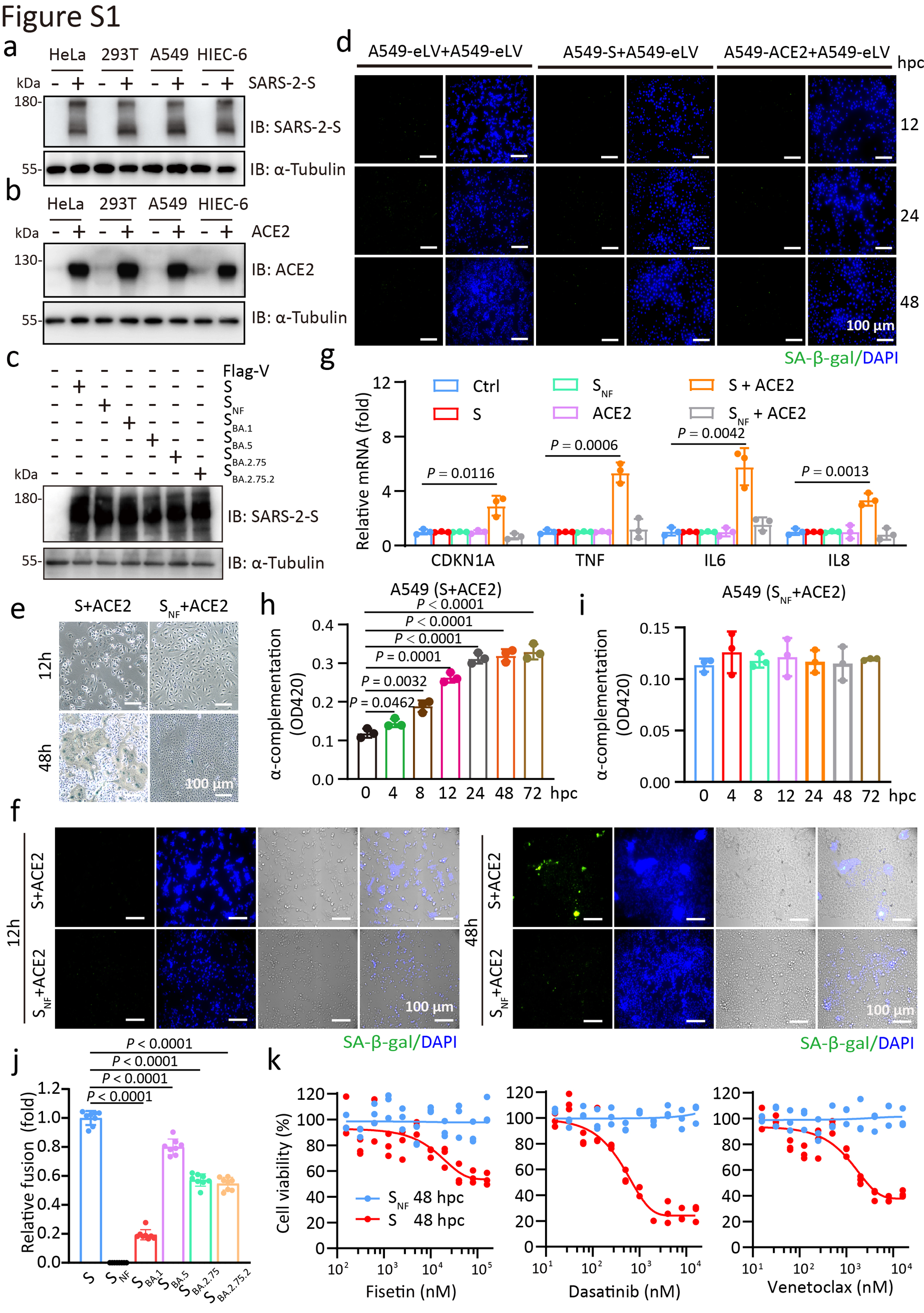

Supplement: S1 Fig — a-b, Immunoblot analysis of SARS-2-S (a) and ACE2 (b) expression in A549, 293T, HeLa and HIEC-6 cells transfected with the indicated plasmids. α-Tubulin was used as a loading control. c, Immunoblot analysis of A549 cells transfected with S, SNF, SBA.1, SBA.5, SBA.2.75 or SBA2.75.2. α-Tubulin was used as a loading control. d, SA-β-gal staining of A549-eLV, A549-S or A549-ACE2 co-cultured with A549-eLV cells for the indicated times. Green, SA-β-gal staining; blue, nuclear DAPI staining. Scale bars represent 100 μm. e-f, SA-β-gal staining (e), fluorescent SA-β-gal staining and bright field imaging (f) of A549-S+A549-ACE2 or A549-SNF+A549-ACE2 cells for the indicated times. Green, SA-β-gal staining; blue, nuclear DAPI staining. Scale bars represent 100 μm. g, Normalized expression of CDKN1A, TNF, IL6 and IL8 transcripts in Ctrl, SNF, S, ACE2, S+ACE2 and SNF+ACE2 cells at 48 hpc by RT-qPCR. h-i, Quantification of the fusion of A549-S+A549-ACE2 (h) and A549-SNF+ A549-ACE2 (i) based on the β-galactosidase α-complementation assay for the indicated times. j, Quantification of the relative fusion ability of four Omicron variants based on the β-galactosidase α-complementation assay. The fusion level of A549-S and A549-ACE2 cocultured cells at 24 hpc was set to 1. k, Cell viability of cocultured A549-S+A549-ACE2 cells and A549-SNF+ A549-ACE2 cells treated with fisetin, dasatinib, or venetoclax for 48 h by CCK8 assay. All images are representative of 3 biological replicates. All quantified data are presented as the mean ± SD of n = 3 independent experiments. Statistical significance was determined with two-tailed Student’s t test (g, h, i, j). (TIF) [file ppat.1012291.s001.tif]

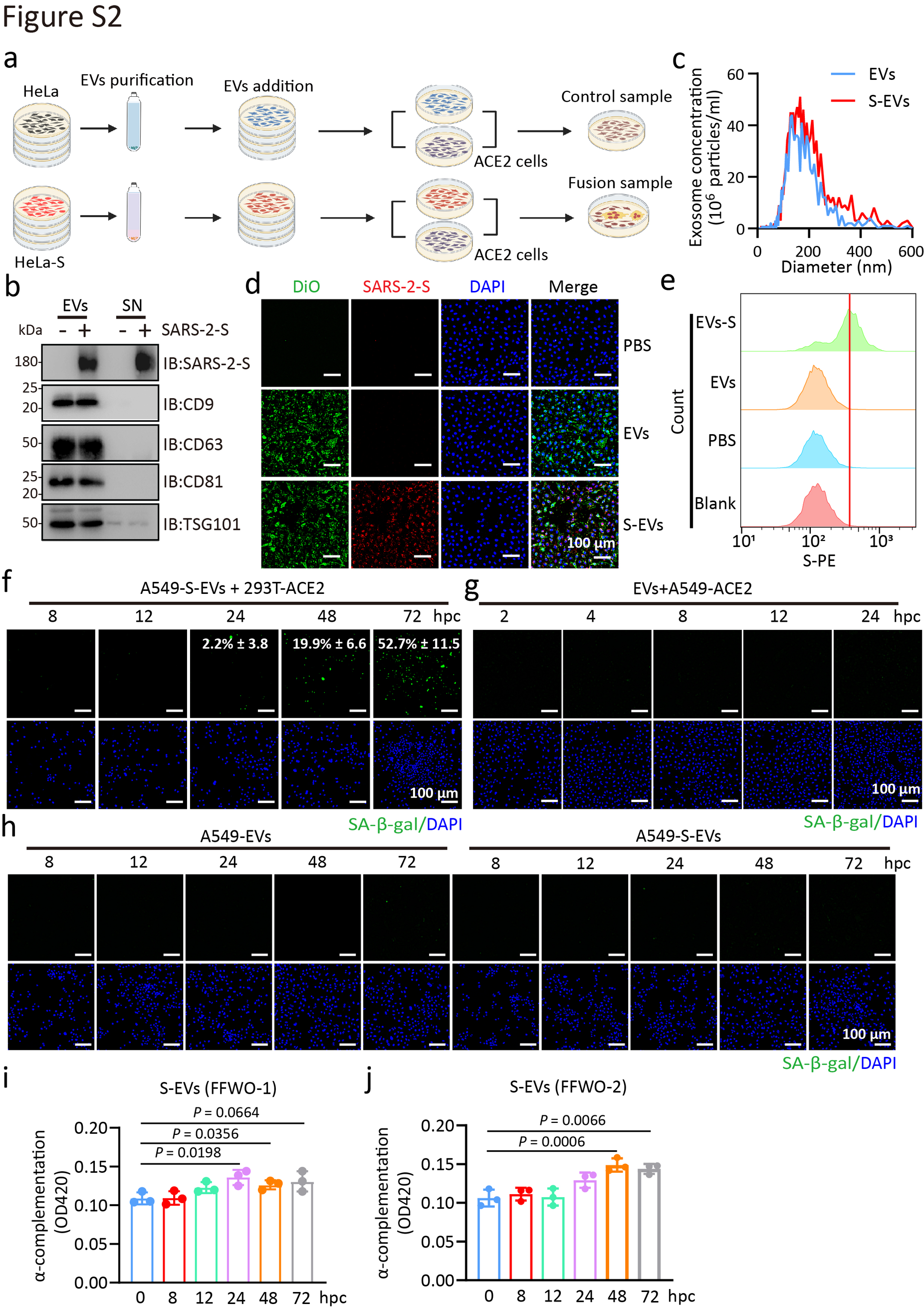

Supplement: S2 Fig — a, Illustration of EV-mediated FFWO. Cells were treated with S-EVs for 24 h, followed by coculture with ACE2-expressing cells for the indicated times. A mixture of A549-EVs and A549-ACE2 cells was used as a nonfusion control. b, Immunoblot analysis of EV markers (CD9, CD63, CD81, and TSG101) and S expression in EVs purified from the supernatant (SN) of HeLa or HeLa-S cells. c, Nanoparticle Tracking Analysis (NTA) analysis of EVs and S-EVs. d, Analysis of DiO-labelled S-EVs or EVs uptake by A549 cells. Green, DiO-labelled EVs; red, S staining; blue, nuclear DAPI staining. e, Flow cytometry analysis of S expression on the cell surface of A549-S-EVs cells. f-g, SA-β-gal staining of A549-S-EVs cocultured with 293T-ACE2 cells (f) or EVs-treated A549-ACE2 cells (g) for the indicated times. Green, SA-β-gal staining; blue, nuclei DAPI staining. Scale bars represent 100 μm. h, SA-β-gal staining of EVs- or S-EVs-treated A549 cells for the indicated times. Green, SA-β-gal staining; blue, nuclei DAPI staining. Scale bars represent 100 μm. i-j, Quantification of the fusion degree of FFWO-1 (i) and FFWO-2 (j) based on the β-galactosidase α-complementation assay for the indicated times. All the images are representative of three independent experiments. All quantified data are presented as the mean ± SD of n = 3 independent experiments. Statistical significance was determined with two-tailed Student’s t test (i, j). S2A Fig created with Biorender.com. (TIF) [file ppat.1012291.s002.tif]

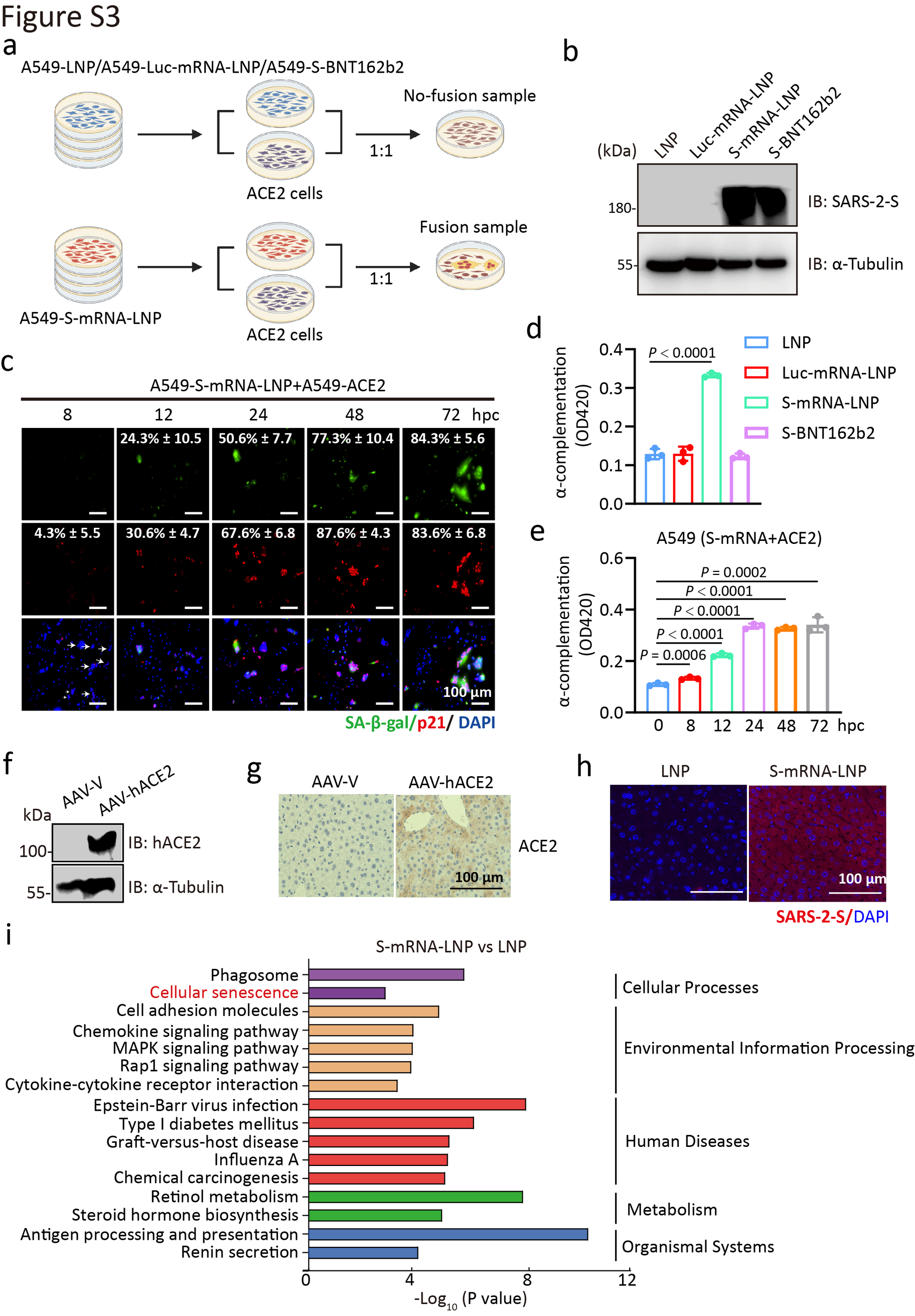

Supplement: S3 Fig — a, Illustration of the cell coculture system mediated by S-mRNAs and S-BNT162b2. Cells were treated with S-mRNA-LNP or S-BNT162b2 for 24 h, followed by coculture with ACE2-expressing cells for the indicated times. A mixture of A549-LNP+A549-ACE2 and A549-Luc-mRNA-LNP+ A549-ACE2 cells were used as a nonfusion control. b, Immunoblot analysis of S expression in A549 cells treated with LNP, Luc-mRNA-LNP, S-mRNA-LNP and S-BNT162b2. α-Tubulin was used as a loading control. c, SA-β-gal and p21 staining of A549-S-EVs and A549-ACE2 cocultured cells for the indicated times. Green, SA-β-gal staining; red, p21 staining; blue, DAPI staining. Scale bars represent 100 μm. d, Quantification of LNP, Luc-mRNA-LNP, S-mRNA-LNP and S-BNT162b2 induced fusion degree based on the β-galactosidase α-complementation assay at 24h. e, Quantification of S-mRNA-LNP induced fusion degree based on the β-galactosidase α-complementation assay for the indicated times. f-h, ACE2 expression or S expression (h) in livers from mice injected with S-mRNA-LNPs or LNPs by immunoblotting (f) or immunohistochemical staining (g). i, Pathways enriched in DEGs identified in the livers of mice injected with LNPs or S-mRNA-LNPs according to KEGG functional pathways at level 2. The bar plot shows significantly dysregulated pathways (P < 0.05), with Fisher’s exact test P values shown on the x-axis. All images are representative of three independent experiments. All quantified data are presented as the mean ± SD of n = 3 independent experiments. Statistical significance was determined with two-tailed Student’s t test (d, e). S3A Fig created with Biorender.com. (TIF) [file ppat.1012291.s003.tif]

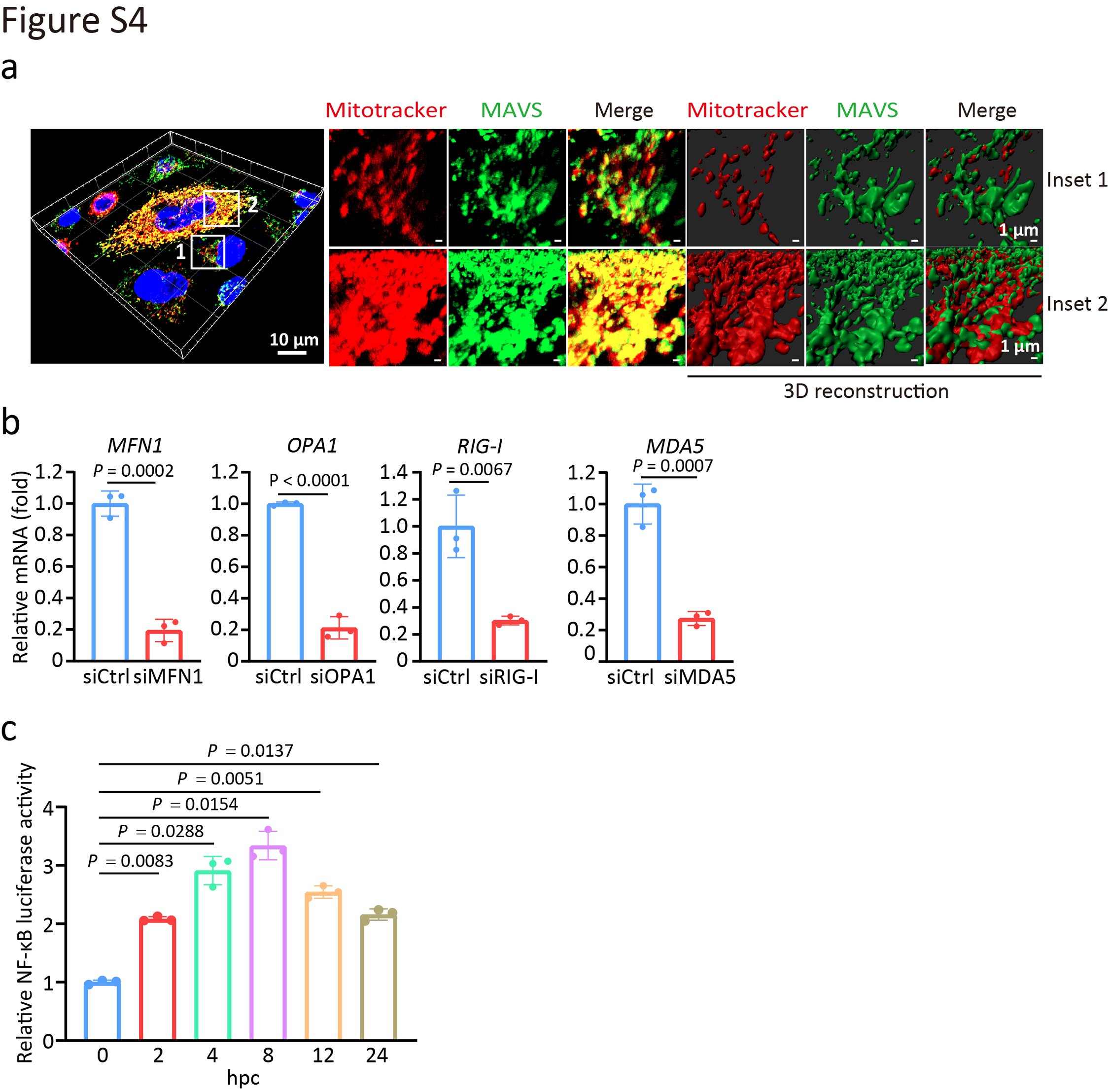

Supplement: S4 Fig — a, Confocal microscopic images of MAVS and mitochondria in cocultured A549 cells at 4 hpc. Red, MitoTracker staining for mitochondria; green, MAVS staining; blue, DAPI staining. A magnified view of the boxed region is shown in the middle panel, and a 3D reconstruction of the Z stacks is shown in the right panel. Scale bars represent 10 μm (whole image) and 1 μm (magnified). b, Knockdown efficiency of siMAVS, siRIG-I, siMFN1, or siOPA1 compared to siCtrl by RT-qPCR. c, Relative luciferase activity of NF-κB in cocultured A549 cells for the indicated times. Luciferase activity was normalized to the values at 0 hpc. All quantified data in this figure are shown as the means ± SDs of n = 3 independent experiments. Statistical significance was determined with two-tailed Student’s t test (b), one-way ANOVA and Bonferroni’s post hoc analysis (c). (TIF) [file ppat.1012291.s004.tif]

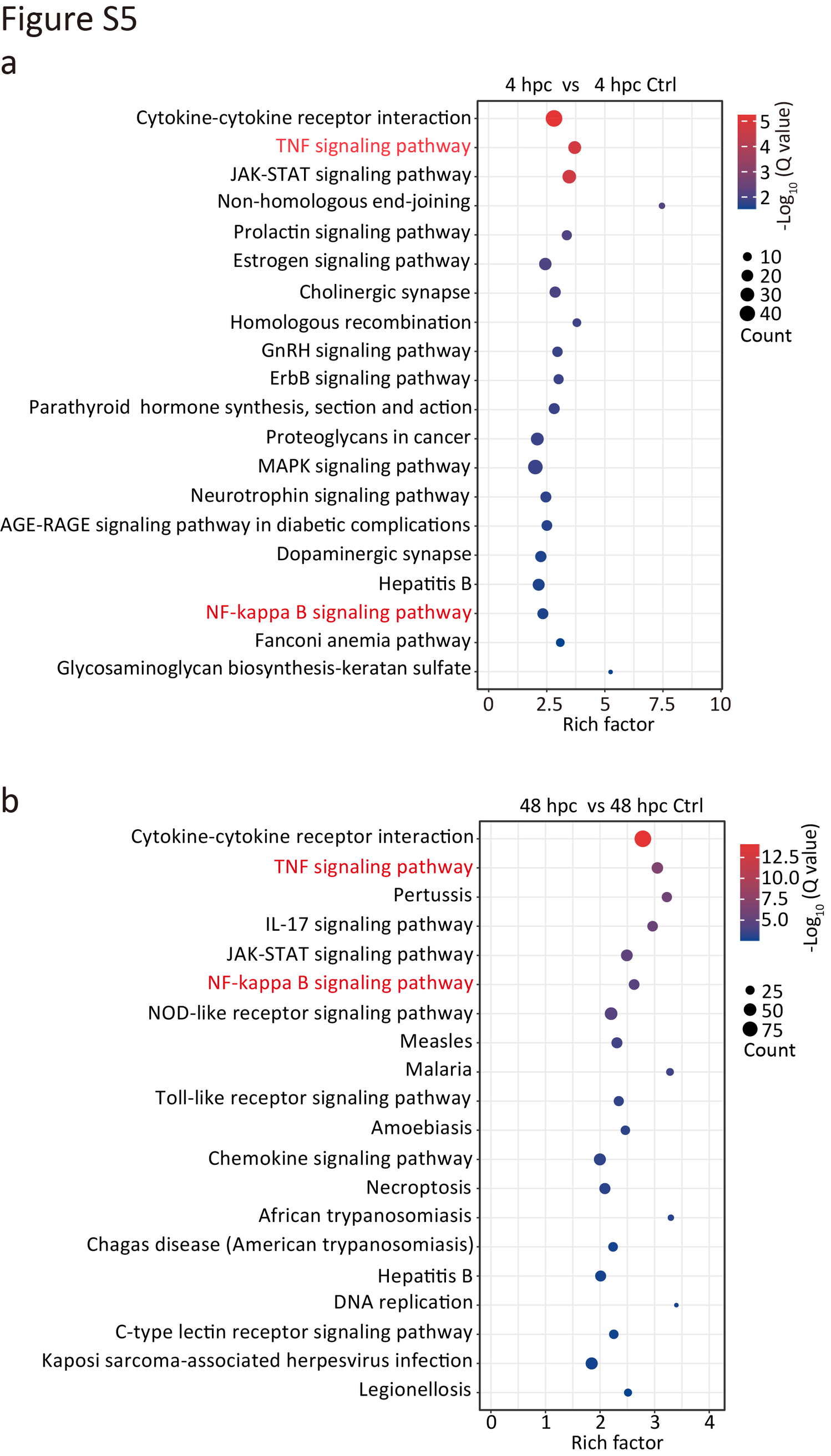

Supplement: S5 Fig — a, b, Twenty most significantly enriched pathways in cocultured A549 cells at 4 hpc (a) and 48 hpc (b) according to KEGG pathway analysis. The enriched terms are shown on the y-axis, and the P values (log transformed) assessing significant enrichment are shown on the x-axis with Fisher’s exact test. The enrichment degree of KEGG was measured by enrichment factors (rich factor), P value and the number of genes enriched in this pathway. (TIF) [file ppat.1012291.s005.tif]

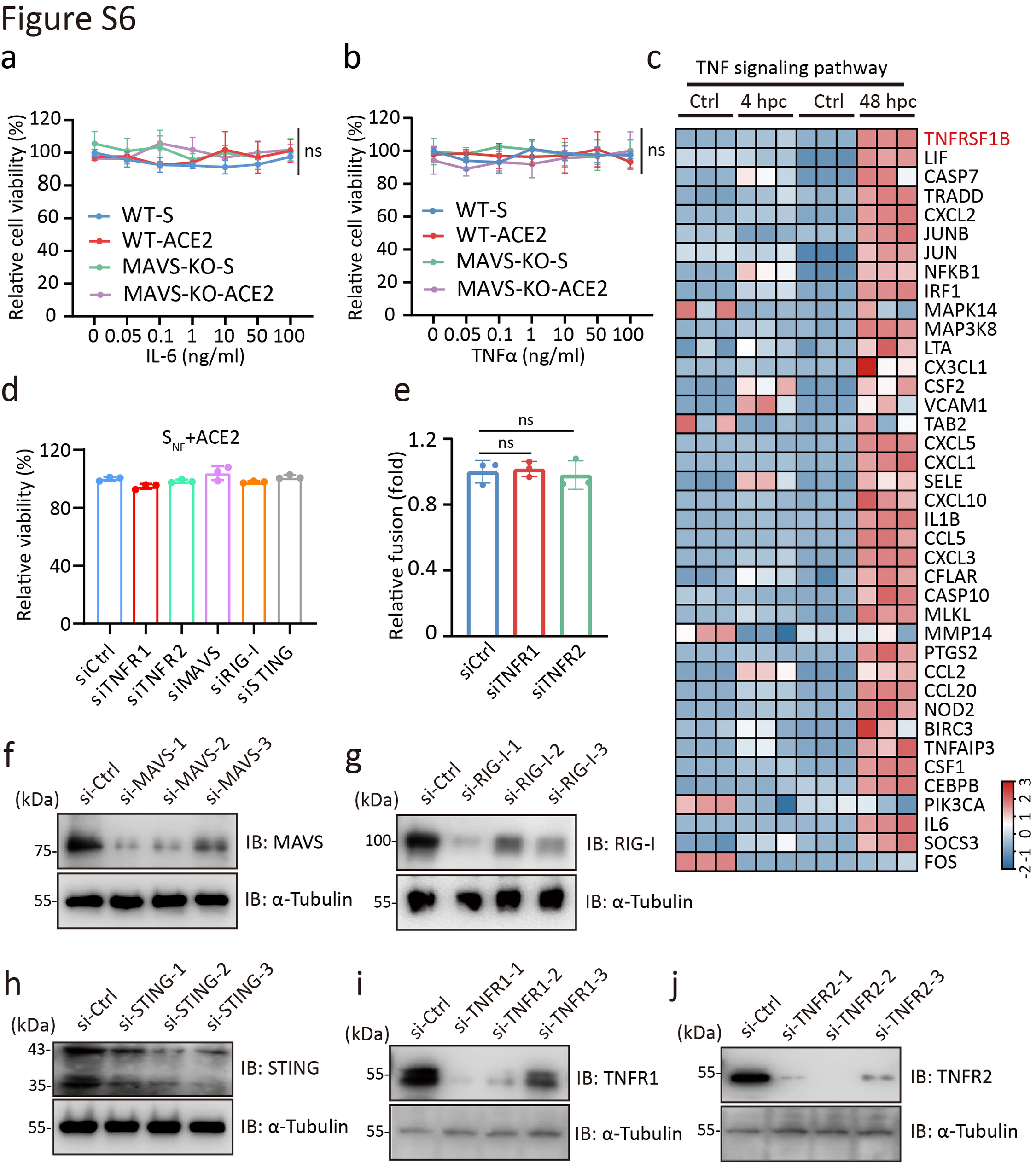

Supplement: S6 Fig — a, b, Relative viability of WT-S, WT-ACE2, MAVS-KO-S, and MAVS-KO-ACE2 cells treated with IL-6 (a) or TNFα (b) at the indicated concentrations. The cell viability of cells treated with PBS was set to 100%. c, Heatmap of the critical DEGs of the TNF signalling pathway in cocultured A549 cells at 4 hpc and 48 hpc. d, Relative viability of A549-SNF and A549-ACE2 cells co-cultured for 24 hours following siRNA-mediated knockdown of TNFRSF1A(TNFR1), TNFRSF1B(TNFR2), MAVS, RIG-I, and STING. The cell viability of cells treated with siCtrl was set to 100%. e, Relative fusion of siTNFR1 and siTNFR2 cocultured A549 cells at 24 hpc by β-galactosidase assay. The fusion level of siCtrl cocultured cells at 24 hpc was set to 1. f-j, Knockdown efficiency of three siRNA sequences targeting MAVS(f), RIG-I(g), STING(h), TNFRSF1A (i), TNFRSF1B (j), as compared to siRNA control (siCtrl) assessed by Western Blot (WB) analysis. All quantified data are shown as the means ± SDs of n = 3 independent experiments. Statistical significance was determined with two-tailed Student’s t test (d), one-way ANOVA and Bonferroni’s post hoc analysis (a, b, d, e). (TIF) [file ppat.1012291.s006.tif]

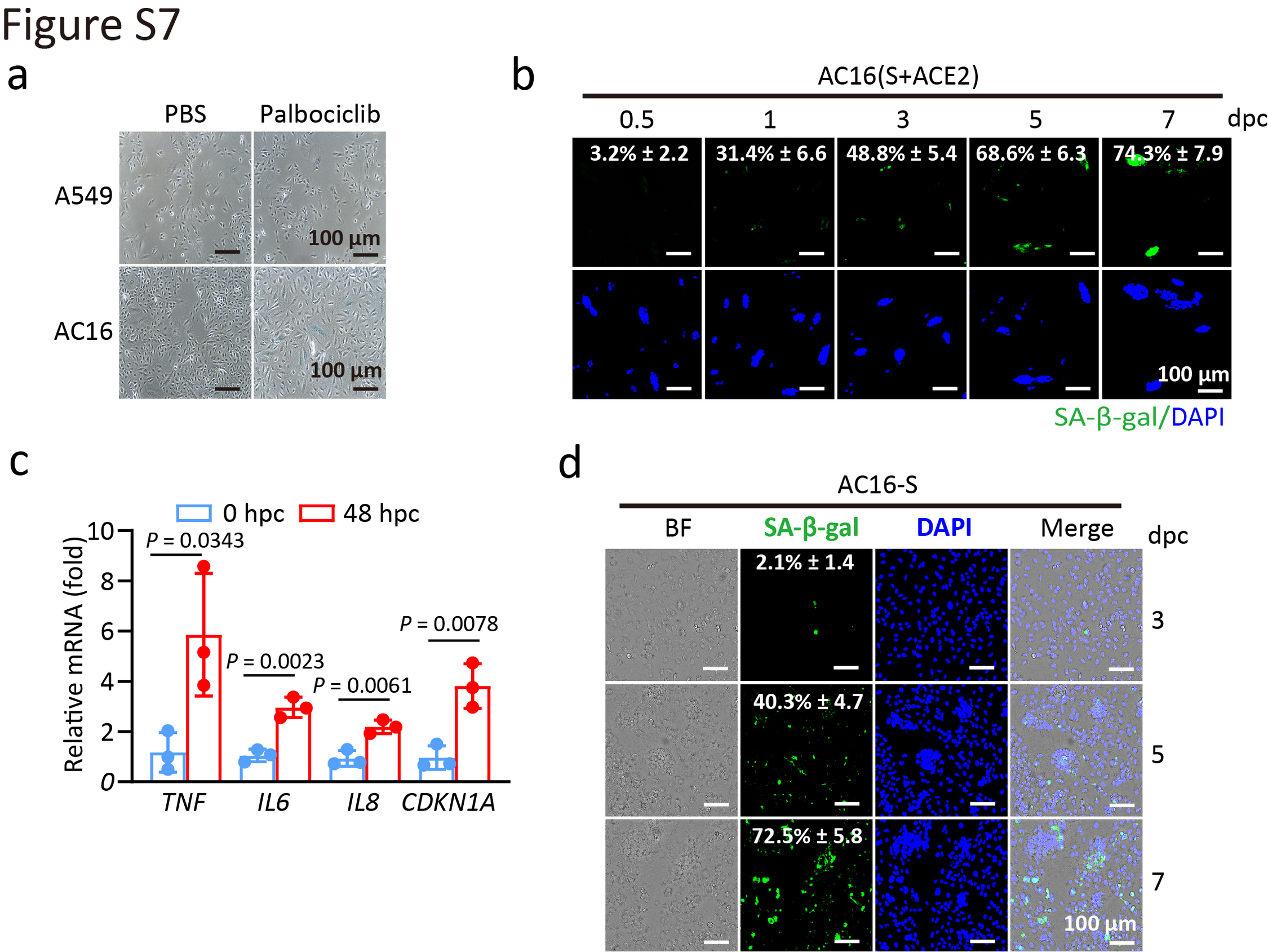

Supplement: S7 Fig — a, SA-β-gal staining of A549 and AC16 cells following treatment with PBS and Palbociclib for 7 days. Scale bars represent 100 μm. b, SA-β-gal staining of AC16-S and AC16-ACE2 cells cocultured for the indicated days (dpc). Scale bars represent 100 μm. Green, SA-β-gal staining; blue, nuclear DAPI staining. c, Normalized expression of TNF, IL6, IL8, and CDKN1A in AC16 cells at 48 hpc from a relative to that at 0 hpc by RT-qPCR. d, SA-β-gal staining of AC16-S and AC16 cells cocultured for the indicated days (dpc). Scale bars represent 100 μm. Green, SA-β-gal staining; blue, nuclear DAPI staining. All quantified data are shown as the mean ± SD of n = 3 independent experiments. Statistical significance was determined using two-tailed Student’s t test (c). (TIF) [file ppat.1012291.s007.tif]

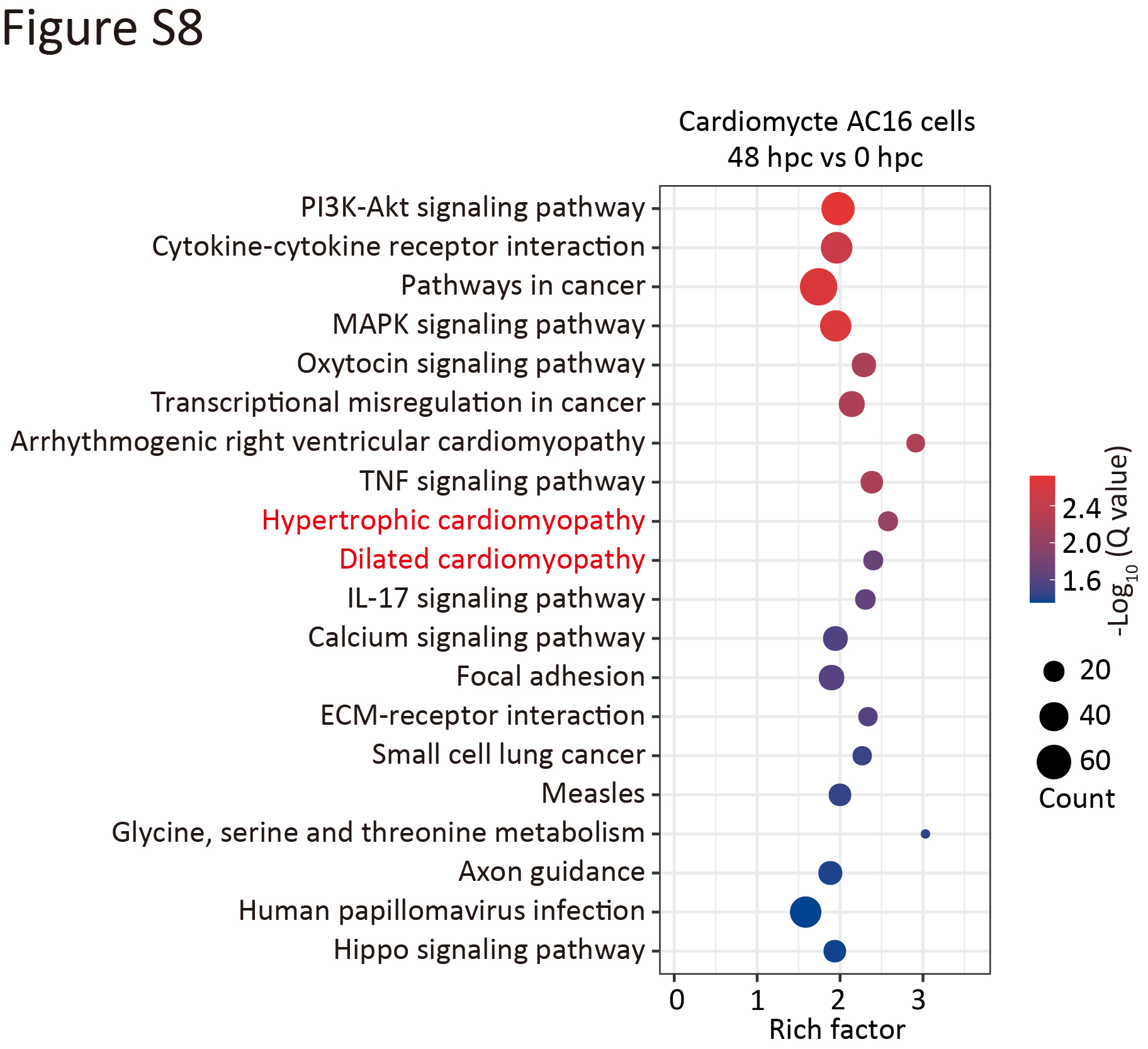

Supplement: S8 Fig — Twenty most significantly enriched pathways in cocultured AC16 cells at 48 hpc versus 0 hpc according to KEGG pathway analysis. The enriched terms are shown on the y-axis, and the P values (log transformed) assessing significant enrichment are shown on the x-axis with Fisher’s exact test. The enrichment degree of KEGG was measured by enrichment factors (rich factor), P value and the number of genes enriched in this pathway. (TIF) [file ppat.1012291.s008.tif]

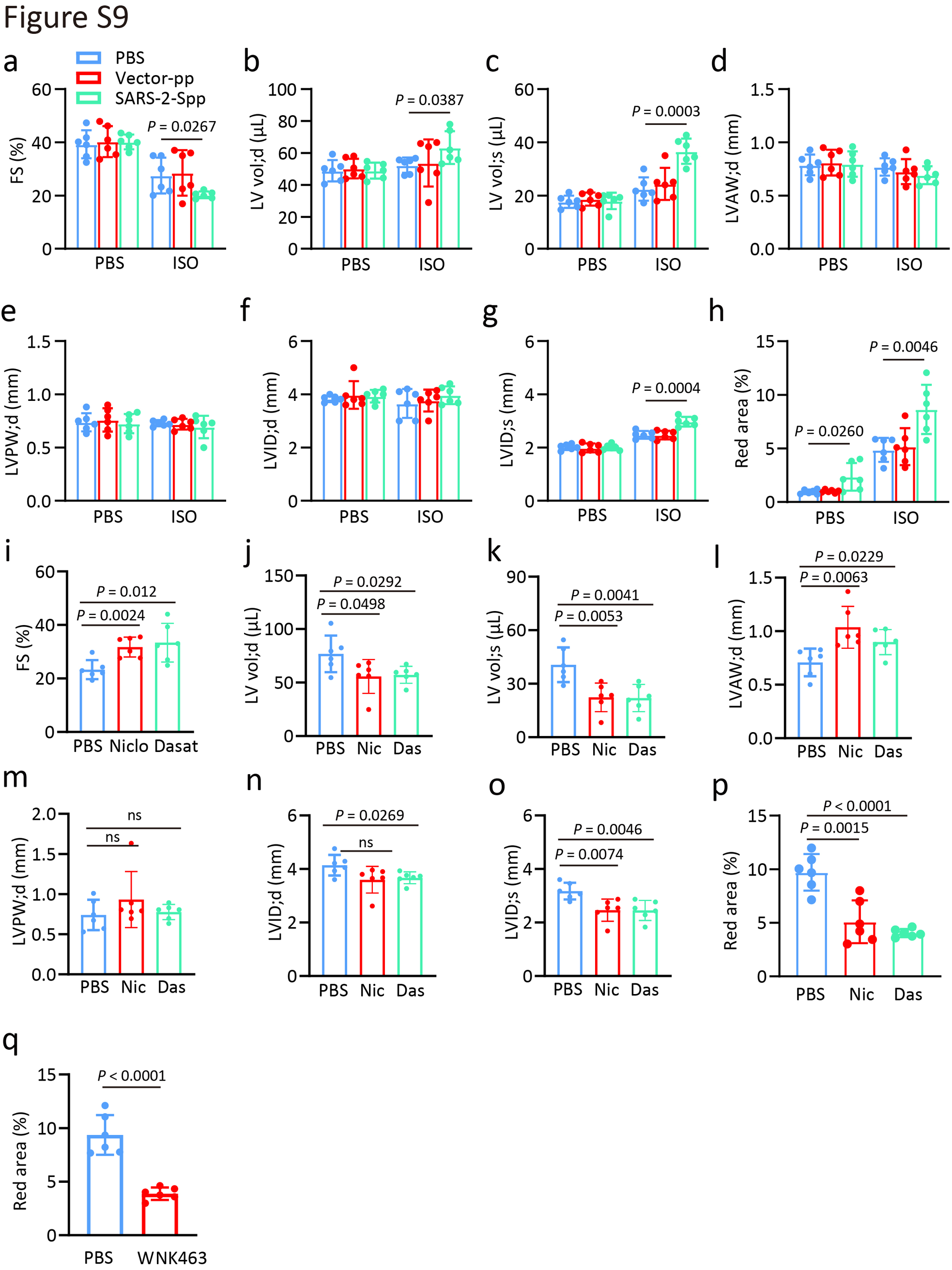

Supplement: S9 Fig — a-g, Fractional shortening (FS) (a), LV end-systolic volumes (LV Vol;d) (b), end-diastolic volumes (LV Vol; s) (c), left ventricular end-diastolic anterior wall thickness (LVAW;d) (d), left ventricular posterior wall thickness (LVPW;d) (e), left ventricular end-diastolic diameter (LVID;d) (f), and left ventricular end-systolic diameter (LVID;s) (g) of PBS, Vector-pp or SARS-2-Spp-infected mice with or without ISO-injection. h, Quantification of the percentage red area in PSR stained cardiac sections from PBS, Vector-pp, or SARS-2-Spp-infected mice, with or without ISO injection, indicating the extent of fibrosis. i, Quantification of the percentage red area in PSR stained cardiac sections from SARS-2-Spp-infected ISO mice treated with PBS or WNK463. j-p, FS (j), LV Vol;d (k), LV Vol; s (l), LVAW;d (m), LVPW;d (n), LVID;d (o), and LVID;s (p) of SARS-2-Spp-infected ISO-mice treated with PBS, niclosamide (Nic), or dasatinib (Das). q, Quantification of the percentage red area in PSR stained cardiac sections from SARS-2-Spp-infected ISO-mice treated with PBS, niclosamide (Nic), or dasatinib (Das). All quantified data are presented as the mean ± SD of n = 6 independent experiments. Statistical significance was determined with one-way ANOVA and Bonferroni’s post hoc analysis (a, b, c, d, e, f, g, h, i, j, k, l, m, n, o, p, q). (TIF) [file ppat.1012291.s009.tif]

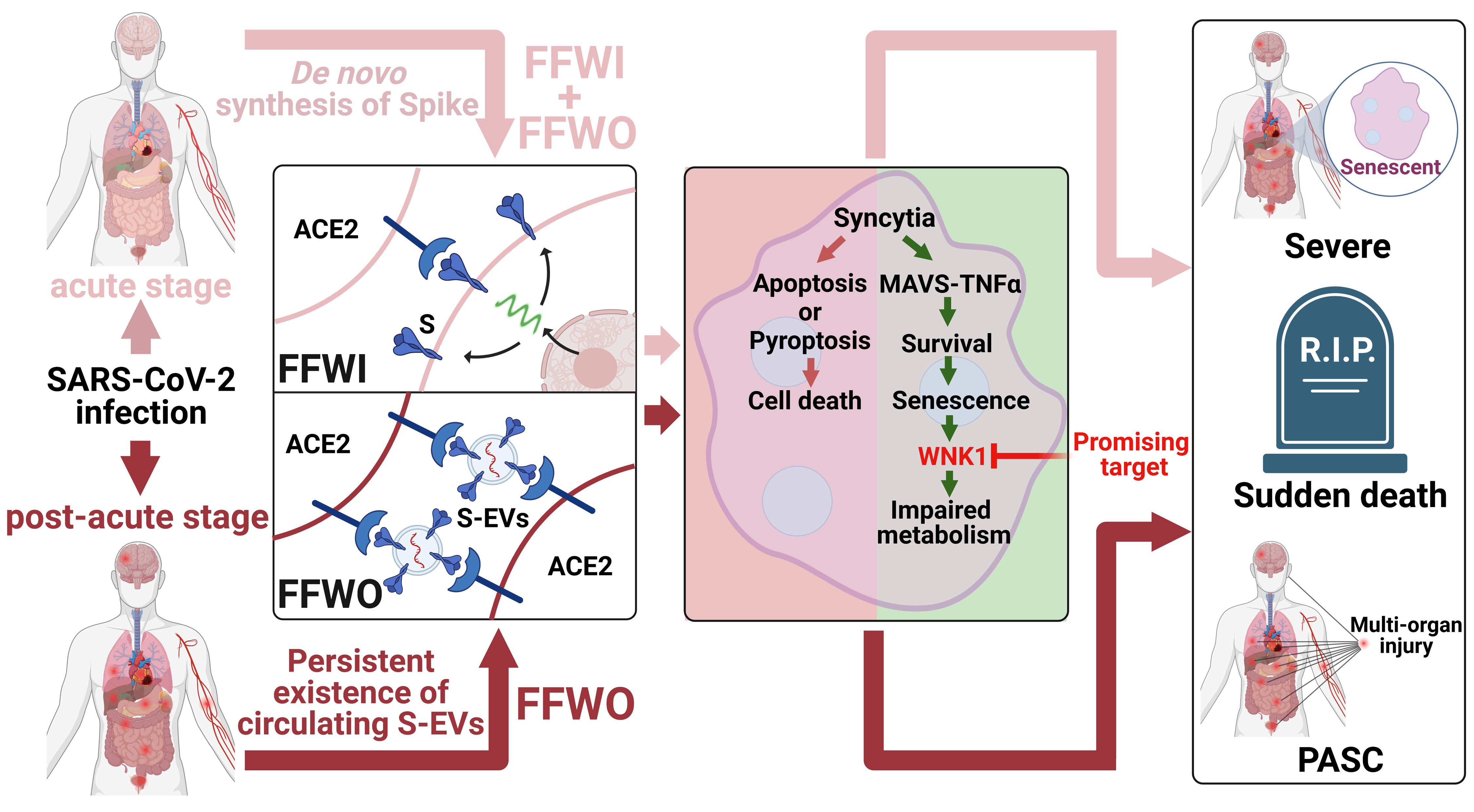

Supplement: S10 Fig — SARS-2-S induced syncytia via FFWI and FFWO manner exhibits a senescence-like phenotype regardless of the cell type or syncytium nucleus number. Mechanistically, RIG-I-MAVS drives the TNFα-dependent survival and senescence fate of SARS-2-S syncytia. The susceptibility of patients with heart failure to more pronounced complications during the acute and post-acute stages of SARS-CoV-2 infection could be related to the senescent outcome of SARS-2-S syncytia, and WNK1 inhibitor may be a druggable target in the management of heart failure associated with SARS-CoV-2 infection. S10 Fig created with Biorender.com. (TIF) [file ppat.1012291.s010.tif]
