# Supplementary material for: SARS-CoV-2 spike-induced syncytia are senescent and contribute to exacerbated heart failure
Source: PLoS Pathog. 2024 Aug 5;20(8):e1012291. doi: 10.1371/journal.ppat.1012291 (PMC11326701; doi:10.1371/journal.ppat.1012291)
Supplement: S1 Table — (PDF) [file ppat.1012291.s011.pdf]

Table.S1 siRNA sequences

Gene	Sense (5'-3')	Antisense (5'-3')
TNF-1 （522）	GCGUGGAGCUGAGAGAUAATT	UUAUCUCUCAGCUCCACGCTT
TNF-2（850）	GGUCUACUUUGGGAUCAUUTT	AAUGAUCCCAAAGUAGACCTT
TNF-3（436）	GCCUGUAGCCCAUGUUGUATT	UACAACAUGGGCUACAGGCTT
TNFRSF1A-1 （866）	GCCUGGAGUGCACGAAGUUTT	AACUUCGUGCACUCCAGGCTT
TNFRSF1A-2 （1016）	GGUGGAAGUCCAAGCUCUATT	UAGAGCUUGGACUUCCACCTT
TNFRSF1A-3 （631）	GGUCAGGUGGAGAUCUCUUTT	AAGAGAUCUCCACCUGACCTT
TNFRSF1B-1 （896）	CCUUGGGUCUACUAAUAAUTT	AUUAUUAGUAGACCCAAGGTT
TNFRSF1B-2 （1360）	CCAAGGAGGAAUGUGCCUUTT	AAGGCACAUUCCUCCUUGGTT
TNFRSF1B-3 （207）	CCGGCUCAGAGAAUACUAUTT	AUAGUAUUCUCUGAGCCGGTT
RIG-I-1 （1969）	CCAGCAAUGAGAAUCCUAATT	UUAGGAUUCUCAUUGCUGGTT
RIG-I-2 （2371）	GCUUCCUUCUGACUAGUAATT	UUACUAGUCAGAAGGAAGCTT
RIG-I-3 （814）	GCCAGAAUCUUAGUGAGAATT	UUCUCACUAAGAUUCUGGCTT
STING-1 （699）	GCAUCAAGGAUCGGGUUUATT	UAAACCCGAUCCUUGAUGCTT
STING-2 （473）	CUGGCAUGGUCAUAUUACATT	UGUAAUAUGACCAUGCCAGTT

STING-3 （529）	CCGGAUUCGAACUUACAAUTT	AUUGUAAGUUCGAAUCCGGTT
MAVS-1 （9951）	GGGACUGUGAAAUGUUAUATT	UAUAACAUUUCACAGUCCCTT
MAVS-1 （6277）	CACCCAACUUCAGUAAUUATT	UAAUUACUGAAGUUGGGUGTT
MAVS-3 （2369）	GGCAGAGGUUUGGAUUUCATT	UGAAAUCCAAACCUCUGCCTT
MDA5-1 （2556）	GCACGAGGAAUAAUCUUUATT	UAAAGAUUAUUCCUCGUGCTT
MDA5-2 （2265）	GCGUAUACUCAUCUUGAAATT	UUUCAAGAUGAGUAUACGCTT
MDA5-3 （746）	CCAACUGCUGAACCUCCUUTT	AAGGAGGUUCAGCAGUUGGTT
MFN1-1 （853）	GCAUCAGAGCCAGAAUAUATT	UAUAUUCUGGCUCUGAUGCTT
MFN1-2 （1156）	GCUAAACAGAUACUAGCUATT	UAGCUAGUAUCUGUUUAGCTT
MFN1-3 （714）	CCUAGAUGCUGAUGUCUUUTT	AAAGACAUCAGCAUCUAGGTT
OPA1-1 （2896）	GCUAUCACCGCAAAUACUUTT	AAGUAUUUGCGGUGAUAGCTT
OPA1-2 （1460）	GCAUGGCUCCUGACACAAATT	UUUGUGUCAGGAGCCAUGCTT
OPA1-3 （642）	GCCUGACAUUGUGUGGGAATT	UUCCCACACAAUGUCAGGCTT
