# Supplementary material for: SARS-CoV-2 spike-induced syncytia are senescent and contribute to exacerbated heart failure
Source: PLoS Pathog. 2024 Aug 5;20(8):e1012291. doi: 10.1371/journal.ppat.1012291 (PMC11326701; doi:10.1371/journal.ppat.1012291)
Supplement: S2 Table — (PDF) [file ppat.1012291.s012.pdf]

Table.S2 Primer sequences

Gene	Forward (5'-3')	Reverse (5'-3')
CDKN1A	ATGAGTTGGGAGGAGGCA	CTGAGCGAGGCACAAGG
MKI67	TATGCCTGTGGAGTGGAAT	GGGTGAGAAAAGGTGCTG
IL6	CCACTCACCTCTTCAGAACG	CATCTTTGGAAGGTTCAGGTTG
IL8	ATACTCCAAACCTTTCCACCC	TCTGCACCCAGTTTTCCTTG
MDA5	GCTGAAGTAGGAGTCAAAGCCC	CCACTGTGGTAGCGATAAGCAG
MFN1	GGTGAATGAGCGGCTTTCCAAG	TCCTCCACCAAGAAATGCAGGC
OPA1	GTGGTTGGAGATCAGAGTGCTG	GAGGACCTTCACTCAGAGTCAC
MMP3	TTTTCTCCTGCCTGTGCT	TTCACGCTCAAGTTCCCT
MMP9	ACGCAGACATCGTCATCC	CCAGGGACCACAACTCG
ACTB	TCTCCCAAGTCCACACAGG	GGCACGAAGGCTCATCA
TNF	ACTTTGGAGTGATCGGCC	GCTTGAGGGTTTGCTACAAC
TNFRSF1A	TGCCAGGAGAAACAGAACAC	TCCTCAGTGCCCTTAACATTC
TNFRSF1B	GTCCACACGATCCCAACAC	TGTCACACCCACAATCAGTC
STING	TCAAGGATCGGGTTTACAGC	GCTTGACTGTATTGTGACATGG
MAVS	ATGGTGCTCACCAAGGTGTCTG	TCTCAGAGCTGCTGTCTAGCCA
RIG-I	CACCTCAGTTGCTGATGAAGGC	GTCAGAAGGAAGCACTTGCTACC
