# Supplementary material for: SARS-CoV-2 spike-induced syncytia are senescent and contribute to exacerbated heart failure
Source: PLoS Pathog. 2024 Aug 5;20(8):e1012291. doi: 10.1371/journal.ppat.1012291 (PMC11326701; doi:10.1371/journal.ppat.1012291)
Supplement: S1 Data — (PDF) [file ppat.1012291.s013.pdf]

Figure 3b

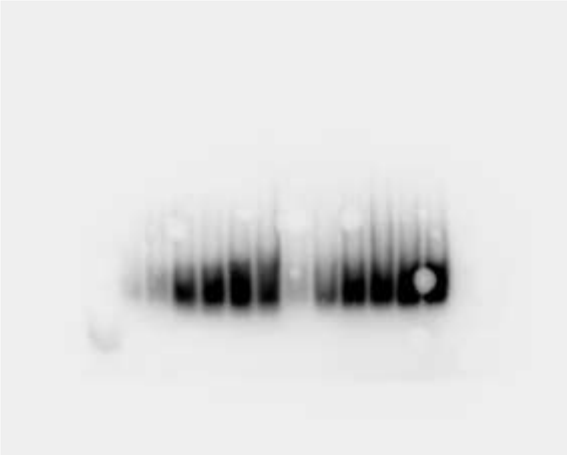

IB: MAVS SDD-AGE

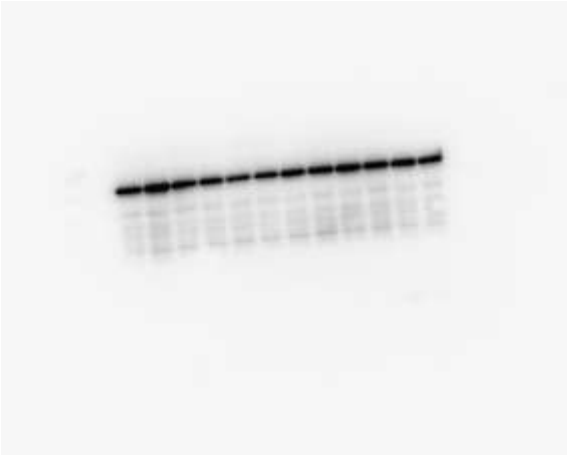

IB: α-Tubulin SDS-PAGE

Figure 3c

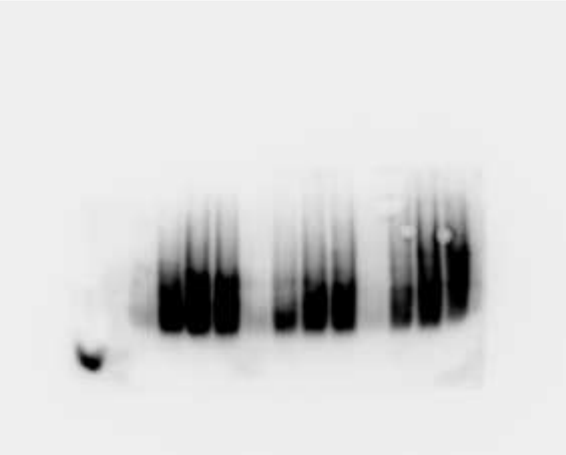

IB: MAVS SDD-AGE

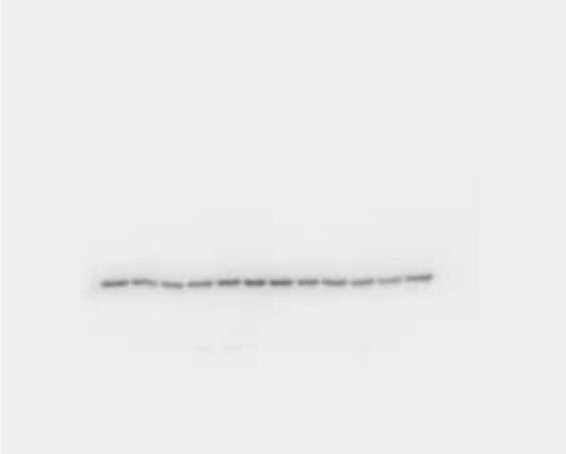

IB: α-Tubulin SDS-PAGE

Figure 3d

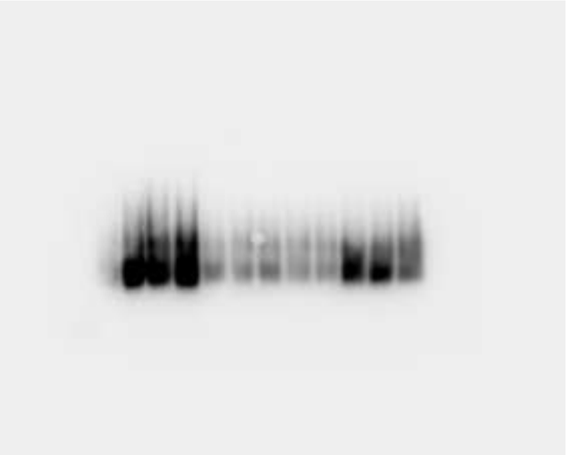

IB: MAVS SDD-AGE

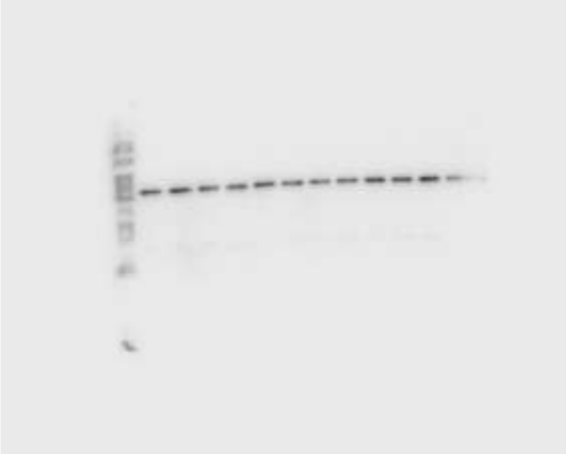

IB: α-Tubulin SDS-PAGE

Figure 3e

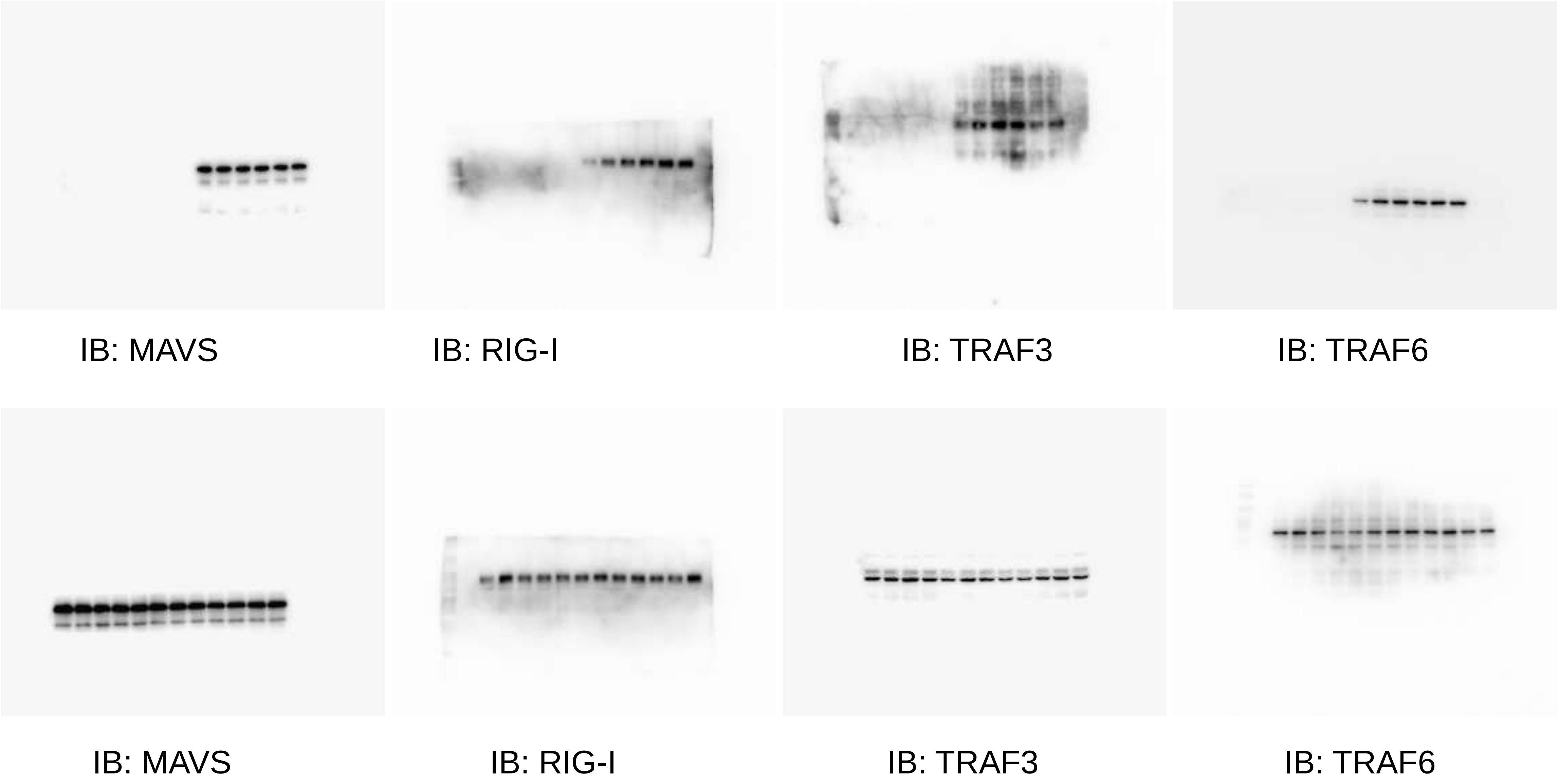
IB: MAVS
IB: RIG-I
IB: TRAF3
IB: TRAF6
IB: MAVS
IB: RIG-I
IB: TRAF3
IB: TRAF6

Figure 3f

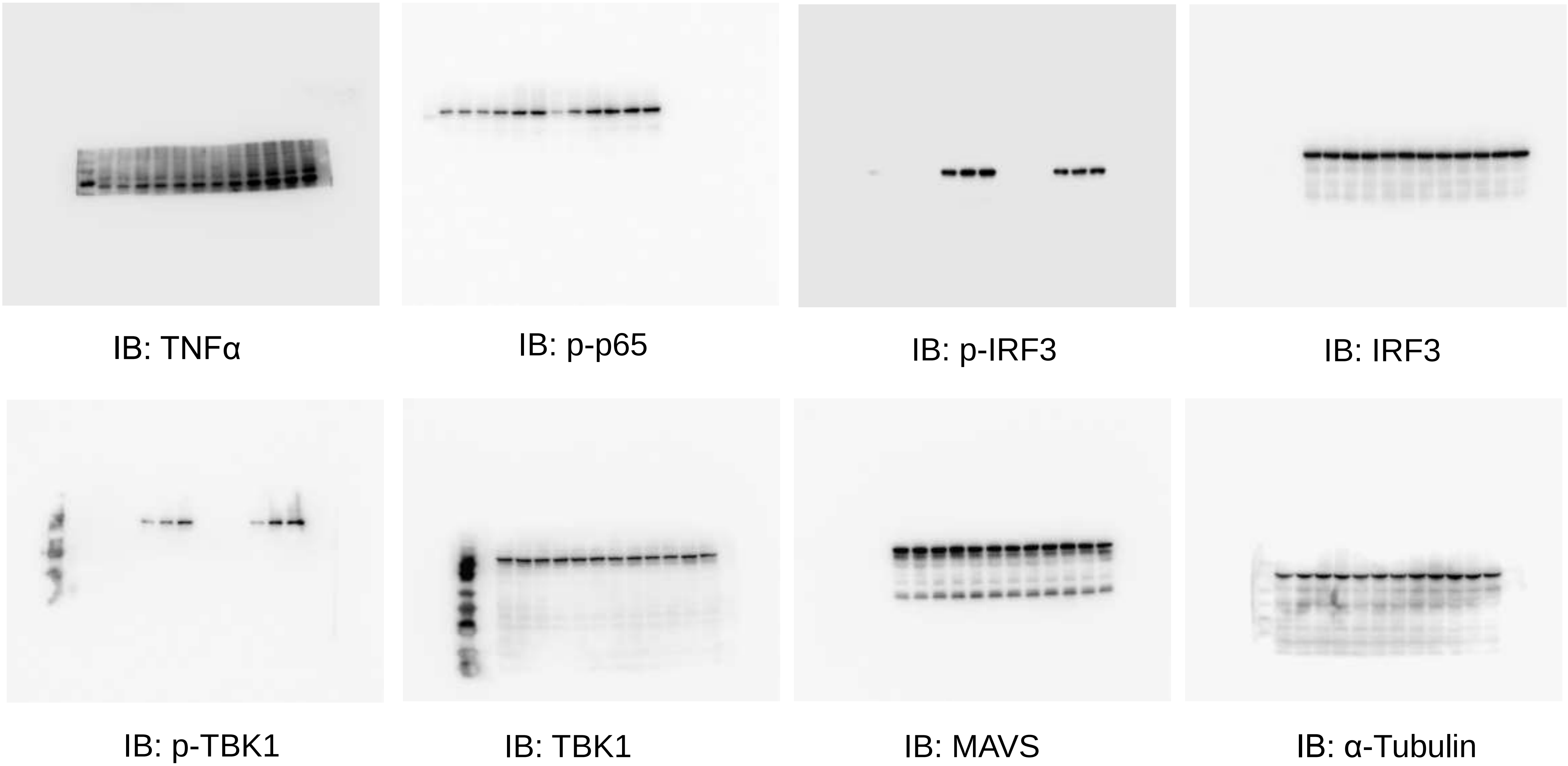
IB: TNFα
IB: p-p65
IB: p-IRF3
IB: IRF3
IB: p-TBK1
IB: TBK1
IB: MAVS
IB: α-Tubulin

Figure 4d

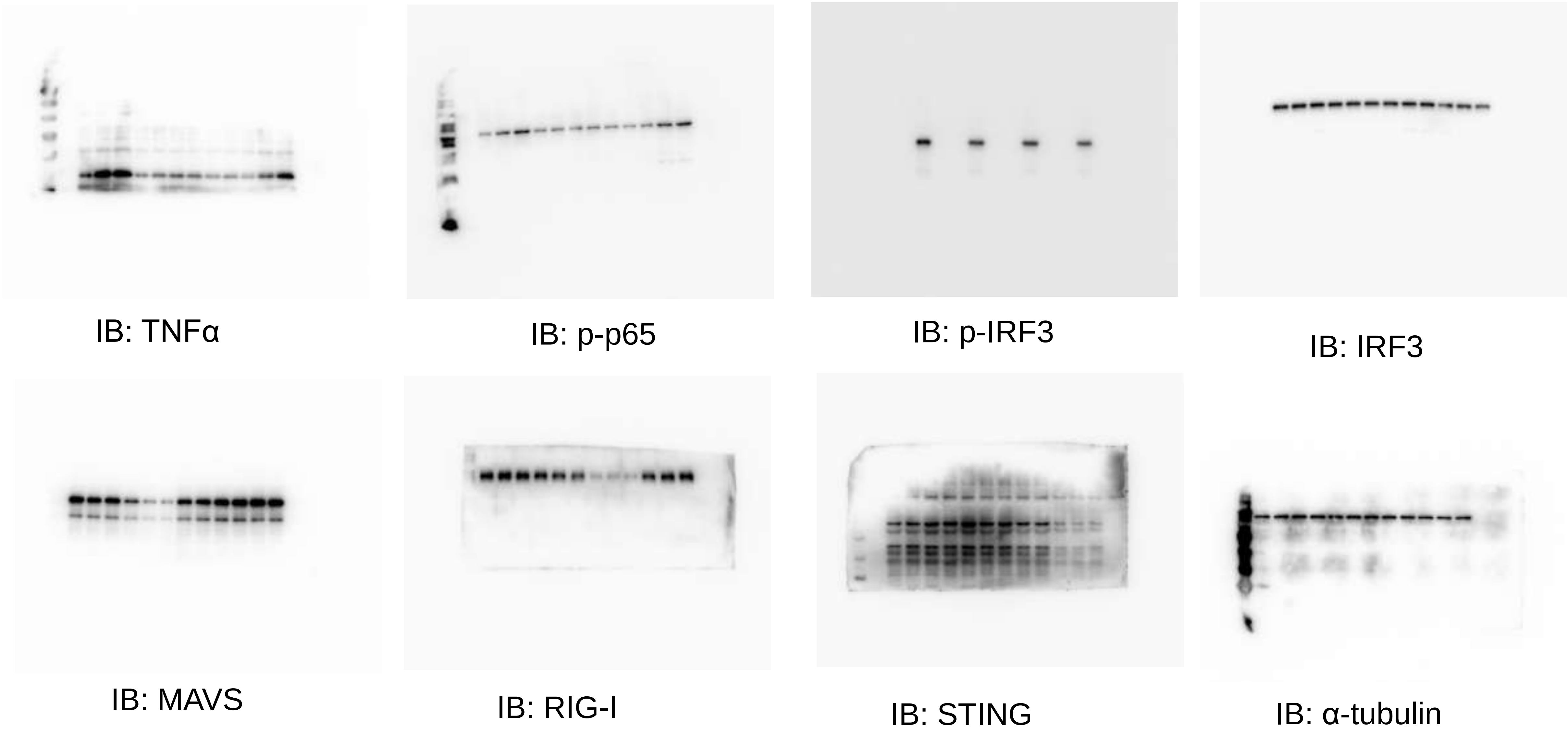
IB: TNFα
IB: p-p65
IB: p-IRF3
IB: IRF3
IB: MAVS
IB: RIG-I
IB: STING
IB: α-tubulin

Figure 5o

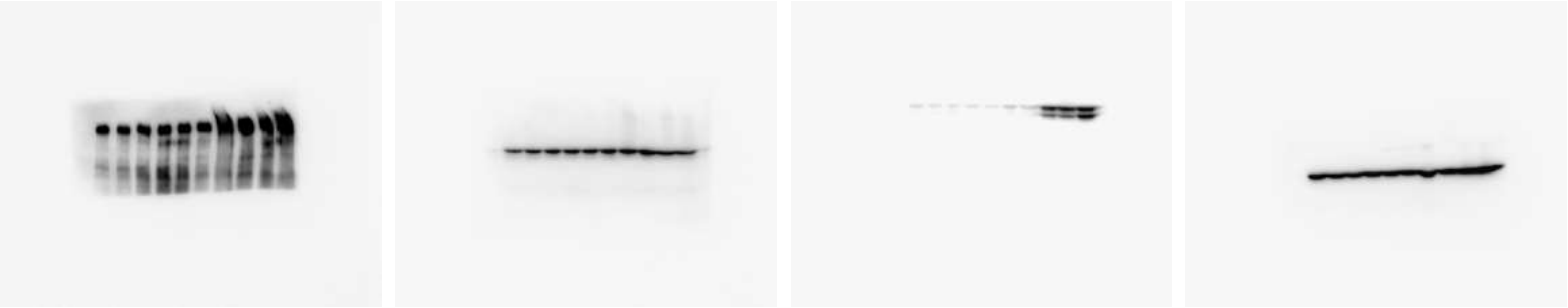

IB: WNK1 IB: SPAK IB: p-SPAK IB: α-Tubulin

Figure 5p

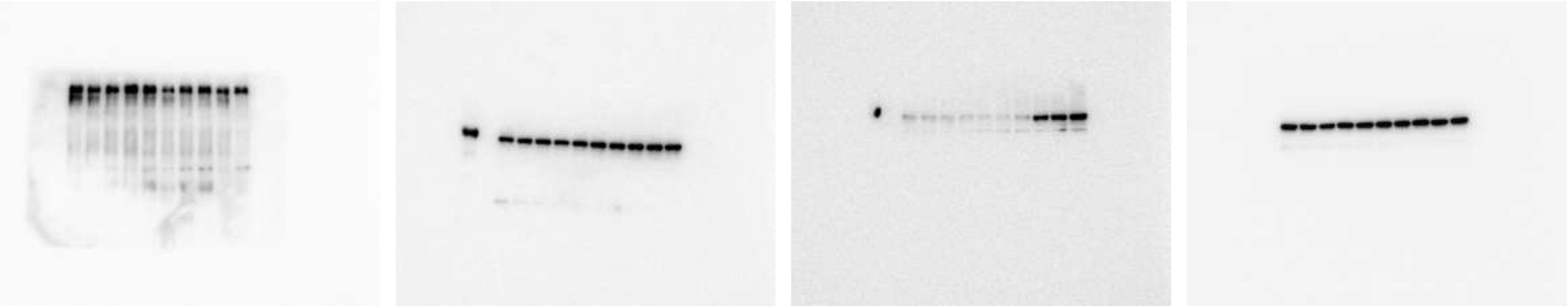

IB: WNK1 IB: SPAK IB: p-SPAK IB: α-Tubulin

Extended Data Fig. 1a

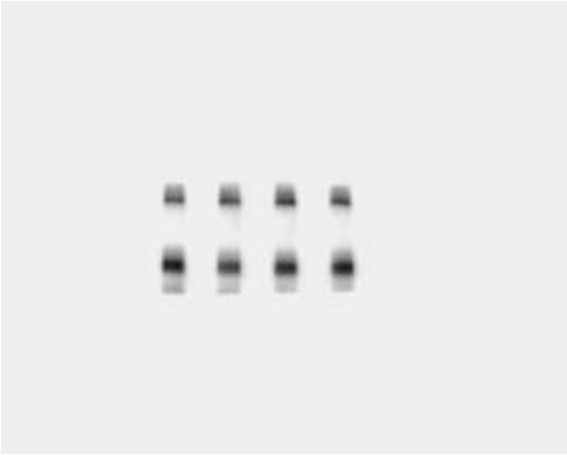

IB: SARS-2-S

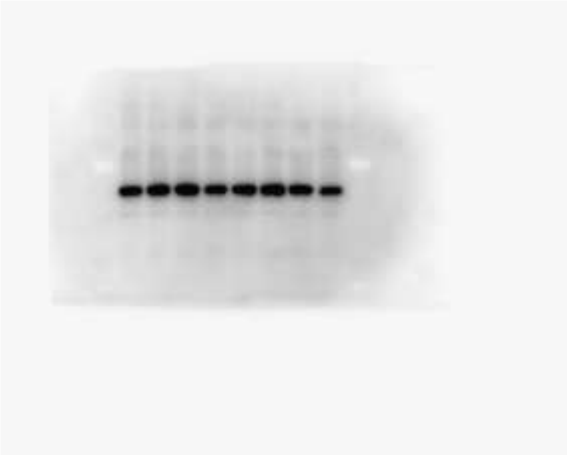

IB: α-Tubulin

Extended Data Fig. 1b

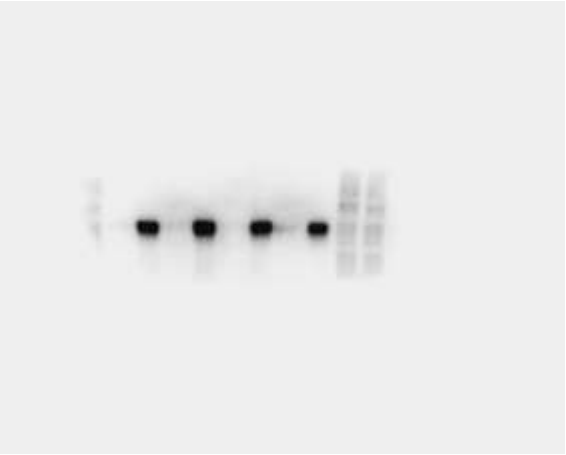

IB: ACE2

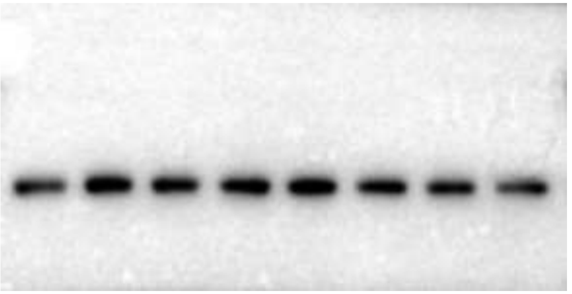

IB: α-Tubulin

Extended Data Fig. 1c

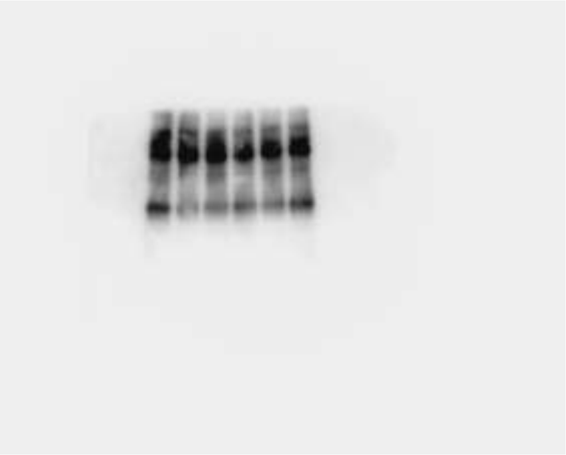

IB: SARS-2-S

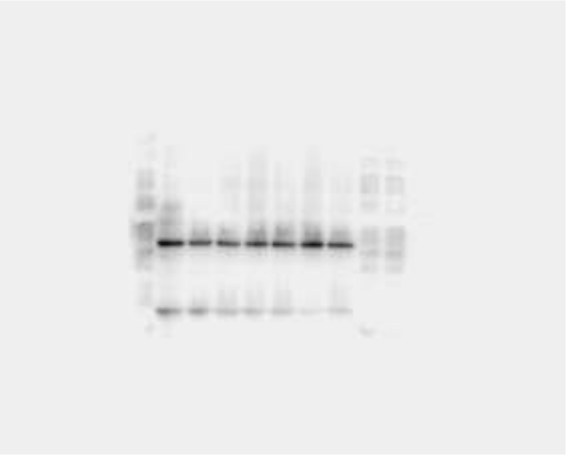

IB: α-Tubulin

Extended Data Fig. 2b

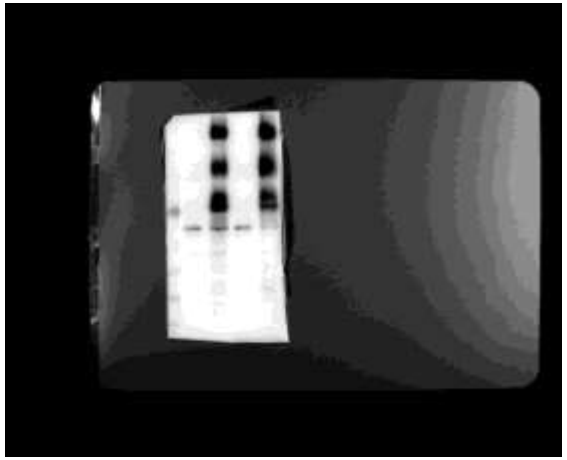

IB: SARS-2-S

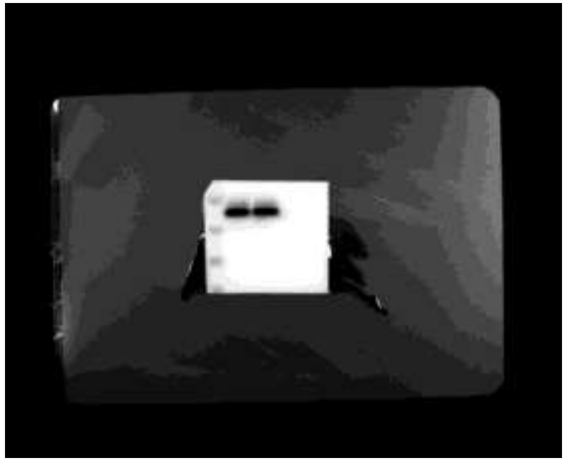

IB: CD9

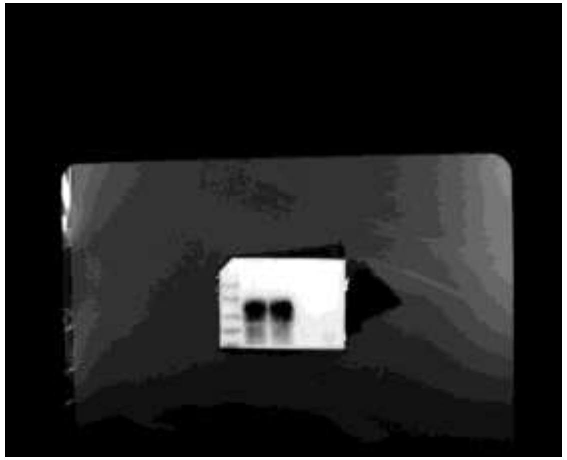

IB: CD63

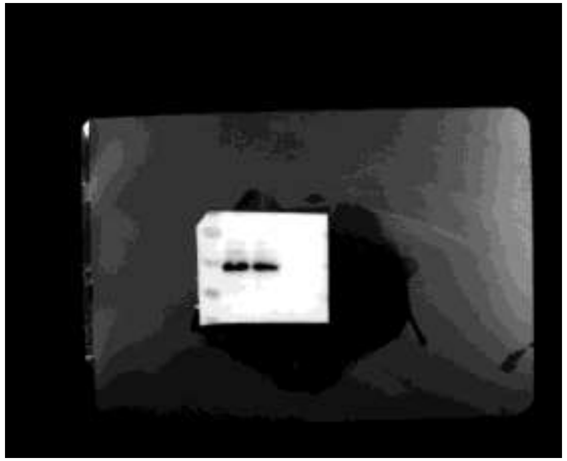

IB: CD81

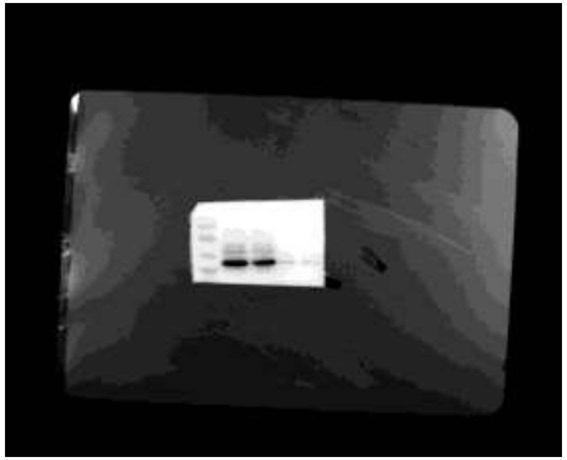

IB: TSG101

Extended Data Fig. 3b

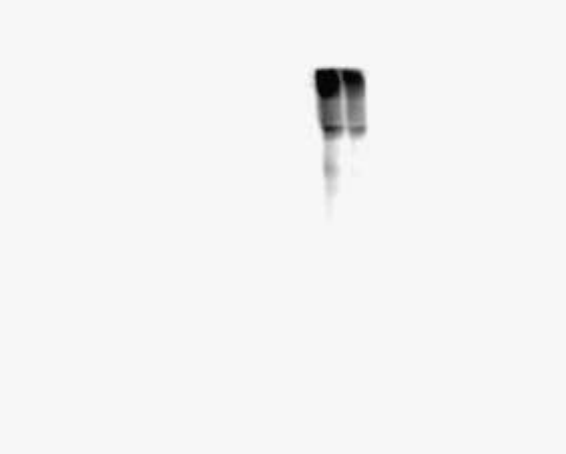

IB: SARS-2-S

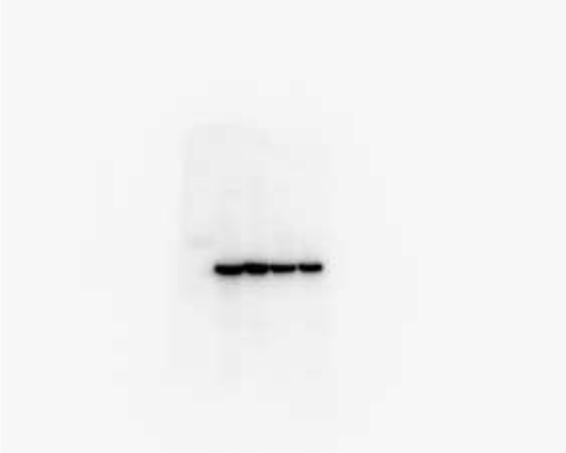

IB: α-Tubulin

Extended Data Fig. 3f

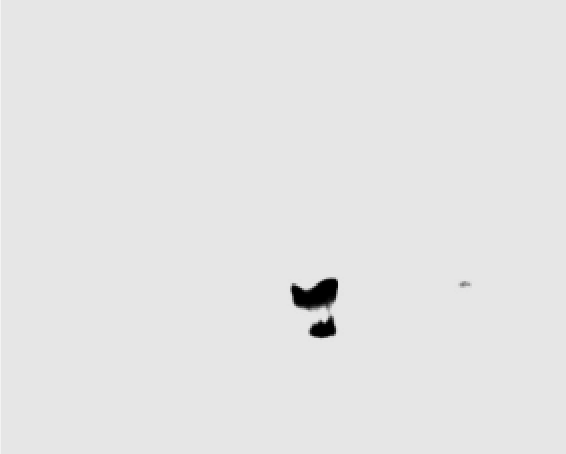

IB: hACE2

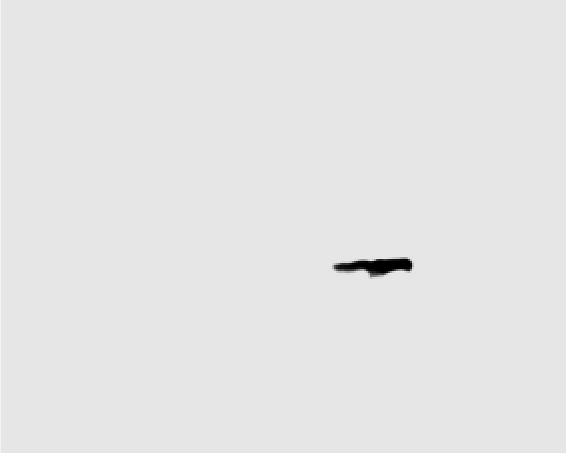

IB: α-Tubulin

Extended Data Fig. 6f

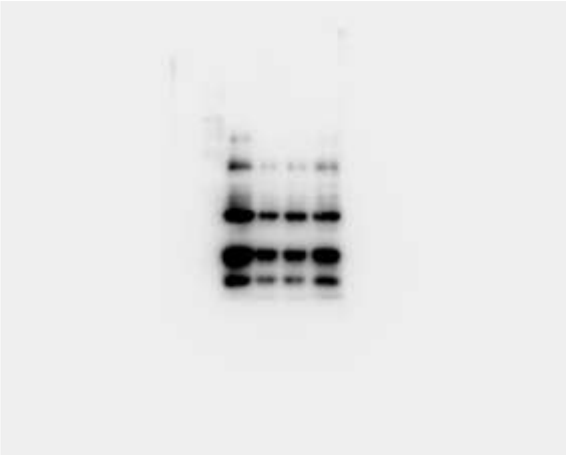

IB: MAVS

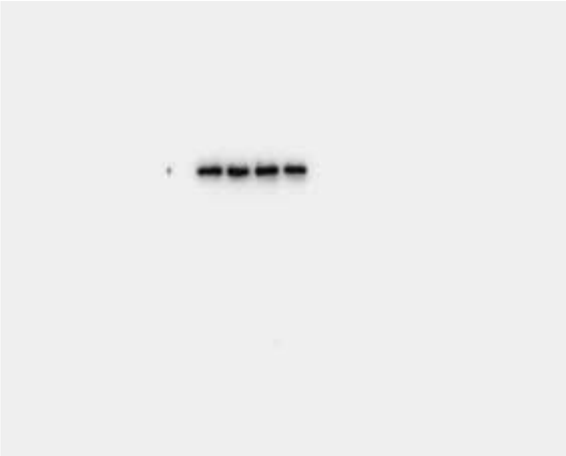

IB: α-Tubulin

Extended Data Fig. 6g

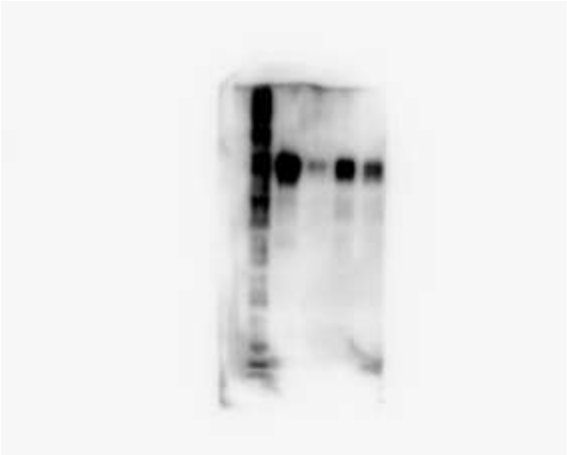

IB: RIG-I

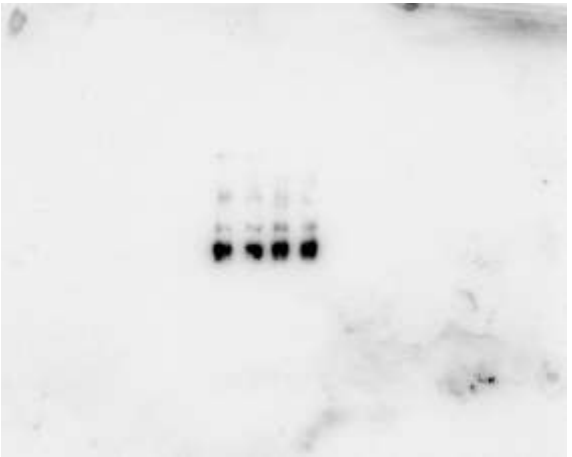

IB: α-Tubulin

Extended Data Fig. 6h

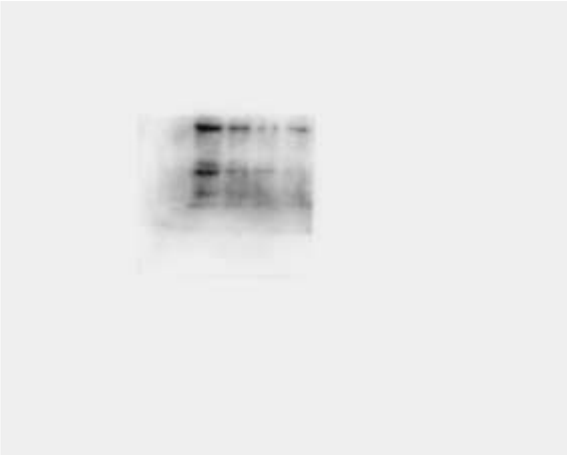

IB: STING

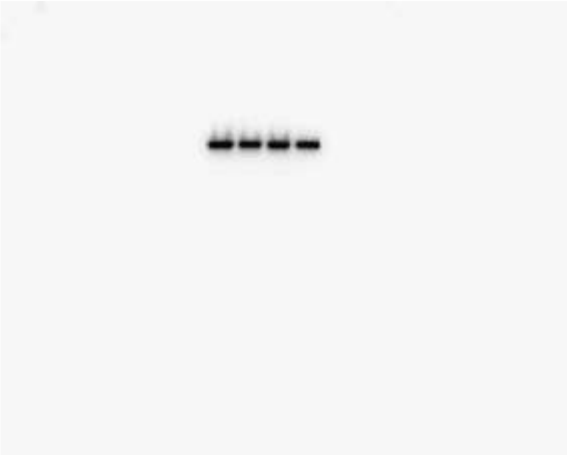

IB: α-Tubulin

Extended Data Fig. 6i

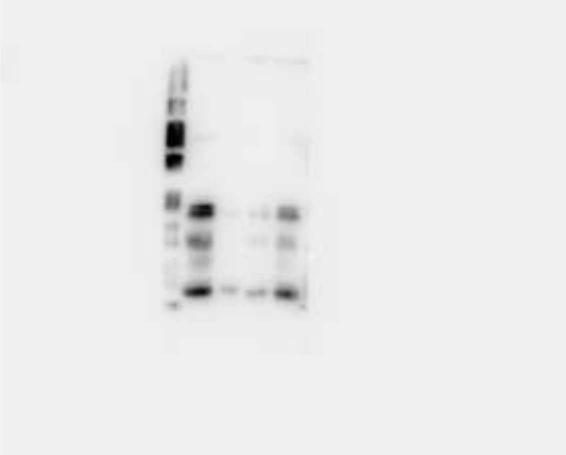

IB: TNFR1

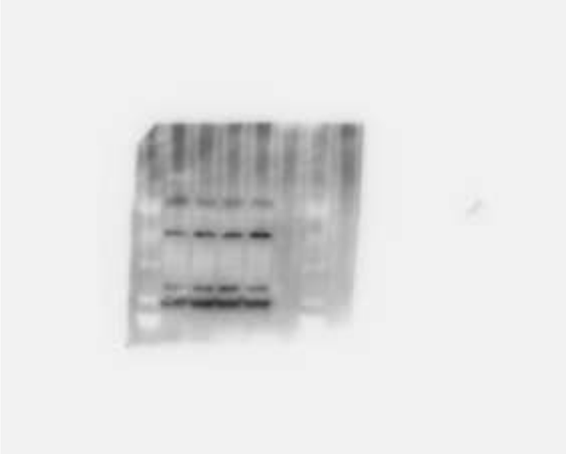

IB: α-Tubulin

Extended Data Fig. 6j

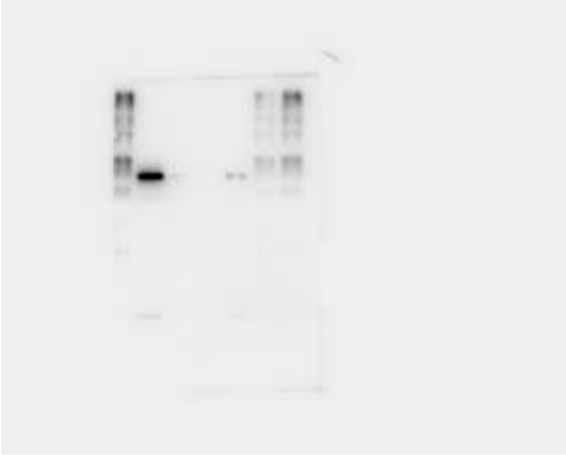

IB: TNFR2

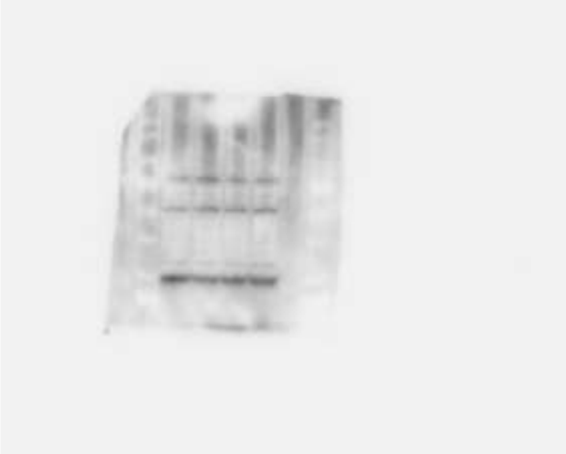

IB: α-Tubulin
